# Exploring Potential Human Health Risks Linked to Heavy Metal(Loid)s in Dietary Fishes: Utilizing Data-Driven and Computational Modelling Approaches

**DOI:** 10.1007/s12011-024-04363-6

**Published:** 2024-09-11

**Authors:** Pritom Bhowmik Akash, Sazal Kumar, Md. Saikoth Jahan, Muhammad Shafiqur Rahman, Md. Assraf Seddiky, Anti Sorker, Rafiquel Islam

**Affiliations:** 1 Department of Civil Engineering, Chittagong University of Engineering & Technology, Chatttogram, 4349 Bangladesh; 2 School of Environmental and Life Sciences, The University of Newcastle (UoN), Callaghan, NSW 2308 Australia; 3 Department of Geography and Environment, Islamic University, Kushtia, 7003 Bangladesh; 4 Materials and Chemical Processing Laboratory, The University of Adelaide, Adelaide, SA 5000 Australia; 5 Department of Public Administration, Shahjalal University of Science & Technology, Sylhet, 3114 Bangladesh; 6 Department of Agricultural Economics, Faculty of Agribusiness Management, Sher-E-Bangla Agricultural University, Sher-E-Bangla Nagar, Dhaka, 1207 Bangladesh; 7 Department of Applied Chemistry and Chemical Engineering, Islamic University, Kushtia, 7003 Bangladesh

**Keywords:** Fish, Heavy metal(loid), Human health risks, Artificial neural network, Dietary safe limit, Bangladesh

## Abstract

There are significant concerns about the risks to human health posed by metal(loid) contamination in dietary fishes in Bangladesh. Therefore, this study aimed to evaluate heavy metal(loid) contamination in fish and their associated health risks using published data from 2000 to 2022. Additionally, the safe limit of fish consumption was estimated using the target hazard quotient (THQ) followed by computational modelling and artificial neural networks (ANN). Results showed that freshwater and herbivorous fishes pose the least non-cancer risks, whereas saltwater and carnivorous fishes pose the highest non-cancer risks to Bangladeshi consumers. However, freshwater and omnivorous fish consumption pose the highest cancer risks compared to all studied metal(loid)s. In particular, among the heavy metal(loid)s, As, Cr, Hg, and Ni pose significant cancer and non-cancer risks to Bangladeshi consumers. On the contrary, the ANN and Decision tree regression (DTR) characterized the dataset, simulation model, or data testing condition, reaching 94.7% accuracy and allowing us to measure the safest fish consumption limit. The herbivorous fishes are less contaminated and allow greater consumption (175.09 g day^−1^). Contrarily, the allowable intake rates of carnivorous and omnivorous fishes are 153.05 and 168.63 g day^−1^, respectively. Besides, the safe consumption rate of saltwater fishes was 156.51 g day^−1^, which was lower than freshwater (180.59 g day^−1^) and euryhaline fishes (182.17 g day^−1^). Therefore, this study will assist fish consumers in selecting less contaminated fish, ensuring safe consumption levels, and ultimately reducing health risks associated with metal(loid) contamination in fish.

**Supplementary Information:**

The online version contains supplementary material available at 10.1007/s12011-024-04363-6.

## Introduction

One of the most concerning contaminants, metals, and metalloids, are pervasive in the environment and enter waterways through both natural processes and anthropogenic activities [1, 2]. Fish contaminated by heavy metal(loid)s can seriously threaten human health via daily dietary intake [2]. Due to fish-specific contamination, lethal toxicity, enduring characteristics, non-biodegradability, and cumulative nature of heavy metal(loid)s, nearly every part of the aquatic environment is currently at risk. Thus, metal(loid)s are constantly contaminating the aquatic habitat, leading to the ongoing destruction of aquatic ecosystems [1, 3, 4].

Heavy metal(loid)s originating from both point sources (i.e., rapid industrial development) and non-point sources (i.e., agricultural runoff) of contaminants can potentially transit to aquatic species if untreated or poorly treated contaminated effluents are released into the aquatic environment [5–7]. The primary causes of metal(loid) contamination in aquatic environments are the excessive use of chemical fertilizers and pesticides, the disposal of agricultural and industrial effluents into nearby watersheds [8]. Metal(loid)s, such as Pb, Cd, Cr, As, and Hg, are considered toxic and can exert a range of harmful effects on biological systems [9], whereas there is a potential necessity of Mn, Fe, Co, Ni, Cu, Zn, and Se for human haemoglobin synthesis, enzyme system efficiency, and vitamin synthesis [2, 10]. Nevertheless, anthropogenic and natural sources discharged metal(loid)s in the water exceeding the WHO and national limits, indicating the possible cause of metabolic disruption in humans as the metal(loid)s enter the food chain [11].

As a riverine country, Bangladesh occupies 118,813 km^2^ of water bodies like rivers, lakes, aqueducts, territorial water, the continental shelf, beaches, and coastal polders [12]. However, the country releases a significant volume of effluents into the lower land and water sources every day, regardless of whether they are treated or not [13]. The untreated or partially treated industrial runoffs from the pharmaceutical, tannery, textile, dyeing, printing, and electroplating manufacturing industries are constantly contaminating surface water sources [3, 14]. A significant amount of metal(loid)s from various milling and processing companies as well as sewage water lines, have been dumped into the rivers in recent years, contaminating most of the surface waterways [15]. In addition, ocean water contamination occurs either by being washed away, blown or dumped via rivers, estuaries, floods, tides, groundwater, or atmospheric deposition, initially to the Bay of Bengal Large Marine Ecosystem (BOBLME) [16]. The shipbreaking operations' pollutants and gases can harm the ocean and shoreline ecosystem as well [16]. For this reason, the sources of seawater pollution near the shoreline are largely consistent across Bangladesh, with the exception that the metal(loid) pollutants from ship-breaking businesses near the Chittagong coast predominantly led to the rise in contamination in that region [16, 17]. Further, sea-based contamination in Bangladesh is mostly brought on by oil spoilage, water bilge, ballast water, the discharge of garbage and pollutants into the ocean from ships, boats, platforms, and artificial structures, as well as seabed operations [16, 18, 19].

Fish, a significant component of aquatic life, cannot escape these contaminants associated with adverse effects [20] since most fishes seek to establish and maintain their territories, which causes them to be exposed to these pollutants. Due to the progressive dilution in water and sedimentation, these pollutants are bioaccumulated in the fish body through eating the benthic and pelagic species in polluted waters [21]. Fish living in the water system are an essential carrier of the transmission, bioaccumulation, and settlement of heavy metal(loid)s [13]. In freshwater fishes, metal(loid)s are bound to the gill surface and gradually bioaccumulates in different body parts. However, in saltwater fishes, metal(loid)s are bioaccumulated by consuming contaminated water as they ingest to maintain osmoregulation [69]. In addition, the fish feed in the environment could be contaminated with metal(loid)s, leading to increased intestinal exposure and offering another pathway to metal(loid) bioaccumulation in fishes [60]. Fish is frequently consumed by humans as a source of protein and other nutritional values [22], for example, at least 265 species of finfish call Bangladesh's waterways home, and they provide over 60% of the nation's entire demand for animal protein [23, 24]. Thus, it creates a significant risk of transferring metal(loid)s to humans through daily fish consumption, which can ultimately affect human health [25].

Although numerous studies have previously evaluated the distribution of heavy metal(loid)s in fishes and their associated risks, the health risk assessment with consuming fish followed by classification based on fish habitat and feeding behaviour by metal(loid)s contamination is completely unknown. Nevertheless, to date, no research has evaluated the potential safe consumption limits of the individual fish species using computation modelling techniques, like Artificial Neural Networks (ANN) and Decision tree regression (DTR). Hence, this study aims to illustrate a comprehensive evaluation of metal(loid) pollution using existing published data and examine the associated health risks along with the recommended daily intake of individual fish or fish categories for Bangladeshi people.

## Materials and Methods

### Data Collection and Homogenization

This study focuses on metal(loid) concentrations in fishes of Bangladesh through a literature review of existing published articles from 2000 to 2022 using a Web of Science topic-based search from all linked databases. The following keywords were used to get most of the published articles: metal* fish* Bangladesh* (number of articles: 251), elements* fish* Bangladesh* (number of articles: 161), elements* crustaceans* Bangladesh* (number of articles: 4), metal* crustaceans* Bangladesh* (number of articles: 10). The articles were downloaded and manually sorted out as per PRISMA flowchart (Supplementary Table S1). The screening of articles for data collection was based on the following major criteria:

(i) the language of the article should be in English (ii) data reported for As, Cd, Cr, Cu, Hg, Mn, Ni, Pb, and Zn, and (iii) metal(loid)s concentration was provided in text, tabular form, in supplementary data as an average concentration of all sampling points or for individual sampling points or individual year and season. Data reported only as range were rejected to ease further analysis. After carefully examining the articles based on the above criteria, 46 relevant research articles were finalized to collect the data (a list of articles is given in supplementary data). Then, as measuring units varied among articles, therefore, to make comparisons, the data set of metal(loid) concentrations in fish were converted into μg g^−1^. In addition, dry weight concentration of metal(loid)s was converted into fresh weight using the following formula as most of the fishes are consumed without drying:

1 $${C}_{w}={C}_{d} [(100-\% of moisture content)/100]$$

where C_w_ and C_d_ are metal(loid) concentrations on a wet/fresh and dry weight basis, accordingly. A moisture content of 80% was considered for fish species [26, 27].

Furthermore, different methodologies and estimation processes were used to quantify metal(loid)s in fishes. Some concentrations were represented as the limit of detection (LOD), the limit of quantification (LOQ), or below the detection limit (BDL), in those cases to calculate the average value, the LOD, LOQ, or BDL was multiplied by 0.5 as per substitution method of left-censored data [28]. Consequently, a considerable variation was identified in the number of samples of each fish species in different studies. For ease of data presentation, the selected fish species were classified into two categories. The first category divides the fish species into omnivorous, carnivorous, and herbivorous according to their feeding behaviors. Secondly, depending on their living adaptation towards salinity, the fish species were divided into freshwater, saltwater, and euryhaline fishes. Finally, every fish species was utilized to calculate the weighted average of the sample numbers and to increase the representativeness of each of the abovementioned fish categories, followed by the following formula [29]:

2 $$Weighted average = \Sigma [({C}_{i}\times {N}_{i})/ \Sigma {N}_{i}]$$

where C_i_ represents the metal(loid)s concentration of each fish species “i”, and “N_i_” is the sample number of the species [29, 30].

Depending on the nature of samples, the number of replications, the methodology employed in metal(loid)s extraction, and instruments used in metal(loid)s quantification, there may exist a significant variation in the final metal(loid)s data. Thus, we acknowledge many biases in the data set and the data should be viewed cautiously.

### Health Risk Assessment

Human health risks were estimated in terms of chronic daily intake (CDI), target hazard quotient (THQ), and carcinogenic risk (CR) from fish metal(oid)s consumption. CDI denotes vulnerability to a quantity of matter per unit of body weight over a considerable amount of time [31, 32]. CDI is calculated using the following formula:

3 $$CDI=[(EF\times ED\times FIR\times C)/ (BW\times AT)]\times {10}^{-3}$$

where CDI is the chronic daily intake, EF is the exposure frequency (365 days year^−1^), ED is the exposure duration (70 years), FIR is the food ingestion rate (g day^−1^), C is the metal concentration in foods (µg g^−1^ fresh weight), and AT is the averaging time for non-carcinogens (365-day year^−1^ × number of exposure years). BW is the body weight (60 kg for adults in Bangladesh) [33]. FIR, the Fish intake rate of Bangladeshi people for fish is 62.6 g person^−1^ day^−1^ [34]. Although these factors vary among communities and various age groups, no specific data is available for each group, particularly on individual age groups, body weight, pregnancy, food consumption rate, and health conditions, which significantly affect potential health risks with metal(loid)s. However, the health risk estimation using this CDI and FIR gives a general overview of potential metal(loid) intake and the probability of cancer and non-cancer health risks for Bangladeshi adults. Consequently, the daily metal(loid) intake rate and health risks of metal(loid)s should be interpreted cautiously.

The THQ was used to estimate non-carcinogenic risks that were provided in the U.S. Environmental Protection Agency (USEPA) Region III’s Risk-based Concentration Table [35]. The non-carcinogenic risks for metal(loid)s through fish consumption were assessed by THQ [36]. The formula used for calculating the THQ is as follows:

4 $$THQ=\left[\left(EF\times ED\times FIR\times C\right)/\left(BW\times AT\times {R}_{f}Do\right)\right]\times {10}^{-3}$$

where THQ is the target hazard quotient, R_f_Do is the oral reference dose (As = 0.0003, Cd = 0.001, Cr = 1.5, Cu = 0.04, Hg = 0.0003, Mn = 0.14, Ni = 0.02, Pb = 0.0035, Zn = 0.3 µg g^−1^ day^−1^) [13, 35]. THQ > 1 indicates a chance of non-carcinogenic health risk, while THQ ≤ 1 indicates no possible health risk to the consumers [37].

The CR (lifetime carcinogenic risk) can be calculated using the equation provided in the USEPA region III risk-based concentration table as follows [38]:

5 $$CR=[(EF\times ED\times FIR\times C\times SF)/(BW\times AT\times {R}_{f}Do)]\times {10}^{-3}$$

where SF is the oral carcinogenic slope factor from the Integrated Risk Information System database (As = 1.5, Cd = 0.38, Cr = 0.5, Ni = 1.7, Pb = 0.0085) [29, 35]. There are no absolute criteria for the acceptable number of additional cancers over a lifetime period. However, the USEPA generally adopts 1(one) additional case of cancer in 1(one) million (i.e., 10^–6^) as a management goal for the government to suggest the point at which management decisions should be considered. The cancer risk surpassing 10^–4^ (1 case of cancer in 10,000) is considered unacceptable [39].

### Numerical Model Establishment and Method Validation

Machine learning has been established as a dependable tool for analyzing and understanding the behavior of a dataset using the interdependent nature of data and understanding how it will behave on the change of the independent variables. In such a manner, it can simulate a special condition to understand and predict any future event. Linear regression and Artificial neural networks (ANN) were employed to discover our data's hidden nature and proposed intricate data clustering and making neural networks (Supplementary Fig. S1). The following is a set of methods intended for regression in which the target value is expected to be a linear combination of the features [40, 41].

6 $$y\left(w,x\right)={w}_{0}+{w}_{1}{x}_{1}+{w}_{2}{x}_{2}\dots {\dots} +{w}_{p}{x}_{p}$$

It designates the vector, *w* = (*w*_1_, *w*_*2*_………,*w*_*p*_) across the module, as coefficient and as intercept. Linear regression constructs a linear model with coefficients that aim to minimize the sum of squared differences between the actual target values in the dataset and the target values predicted by the linear approximation.

In contrast, the ANN utilizes the procedure for calculating the gradient of the loss function which is expressed as follows [42, 43]:

7 $$\stackrel{`}{\omega }=\omega -\eta [\alpha \{\delta R(\omega )/\delta R(\omega )\}+\{\delta Loss/\delta \omega \}]$$

where “*η”* is the learning rate that controls the step size in the parameter space search. *“Loss”* is the loss function used for the network. To feed the data to the model, the textual data, such as fish type, fish name, etc., was encoded into categorized numerical data. A flag variable for every metal(loid), that contains binary values (0 or 1) was created. Where “0” will be the concentration under the safe limit on the other hand “1” denotes exceeding the safe limit. Required information for decoding textual data is provided in the supplementary data. For validation, we split data into train and testing sets 90% and 10%, respectively of the total data. After the model was fed with the training dataset (90% of data), we utilized 10% of the testing dataset for the model's validity. The model succeeded in simulating the condition with 94.7% accuracy. For the interpretation of the model, we have used the Shapley value, which allows us to decode the numerical output and its driving forces (Supplementary Fig. S2). It can be noted that the classification of various fish species was performed by open searching on Google, and different search results were found for the same fish species, therefore, this may minimally affect the data curation and presentation, and finally, the safe consumption limits of every single species may vary which should be dealt with caution.

The computer model-based approach and possible guidelines have been evaluated based on the implemented model, it uses possible daily intake based on the metal(loid) contamination status of individual fish and accepts no human health risks. A possible safe consumption limit can be calculated assuming the THQ = 1 and the following equation:

8 $$FIR=[(BW\times RfDo\times AT)/(EF\times ED\times C)]\times {10}^{3}$$

where EF is the exposure frequency (365 days year^−1^), ED is the exposure duration (70 years), FIR is the food ingestion rate (g day^−1^), C is the metal(oid)s concentration in foods (µg g^−1^ fresh weight), and AT is the averaging time for non-carcinogens (365 days year^−1^ × number of exposure years). BW is the average body weight (60 kg for adults in Bangladesh) [33]. Supplementary Fig. S1 provides a simplified flow diagram of Artificial Neural Network (ANN) processes and data clustering using the driven data of fish types on habitat and feeding behavior, along with consideration of THQ values defining the internal and hidden nature of the data.

### Statistical Analysis

Principal Components Analysis (PCA) is a multivariate ordination technique in which samples of metal(loid)s found in various fish species are grouped based on their habitats—namely, freshwater, seawater, and euryhaline. Furthermore, the concentration of heavy metal(loid)s in these fishes is also clustered according to their feeding habits, classifying them as omnivorous, carnivorous, or herbivorous based on Google searches with individual fish species.

The crux of PCA lies in representing these samples on principal components (PCs), which serve as reduced-dimensional representations of correlated independent variables, e.g., metal(loid)s. The eigenvectors, also known as coefficients, accompany these components and elucidate the strength of the connection between individual metal(loid)s and the respective PC. Notably, eigenvectors boasting loadings exceeding 0.7 are deemed to exhibit a robust correlation with the PC. Assessing the accuracy of the PCs in portraying the solid relationship between samples in the high-dimensional space is encapsulated by the concept of percentages of variance explained as a percentage gleaned from eigenvalues. To facilitate effective analysis, the data undertook a logarithmic transformation of the form log (x + 1) and normalization before their incorporation into the PCA process, utilizing JMP Pro. 16.

## Results

### Metal(Loid)s in Fishes

The descriptive statistics of the concentration of metal(loid)s in fishes of Bangladesh are provided in Tables 1 and 2. There are differences in the content of metal(loid)s among the fish types and the specificity of metal(loid)s. The mean concentration of metal(loid)s represented in Table 1, highlights that the content of Cr, Cu, Mn, Ni, and Zn was higher in freshwater fishes than in seawater and euryhaline fishes. On the contrary, the mean concentrations of As, Cd, Hg, and Pb were higher in seawater fishes compared to fish from other habitats. Due to the ability of fishes to adapt over a wide range of salinity, euryhaline fishes managed to yield a status between freshwater and seawater fishes in terms of the mean concentration of all studied metal(loid)s except for the lower concentration of Cu, Ni, and Pb. In Bangladesh, the high content of Cr, Pb, and Zn in omnivores and Cd and Hg in carnivores, which are in line with the results found in fish from Taihu Lake, China [29]. Bangladeshi omnivores showed higher content of Cr, Ni, Pb, and Zn than carnivores and herbivores (Table 2). However, the carnivores showed maximum content of As, Cd, and Hg and minimum content of Cr, Cu, Mn, and Zn. Minimum concentrations of As, Cd, Hg, Ni, and Pb and maximum concentrations of Cu and Mn were estimated in herbivores. Therefore, carnivorous seawater fishes of Bangladesh contain higher concentrations of As and Cd, whereas omnivores living in freshwater have higher content of Cr, Ni, and Zn. The Cu and Mn concentrations are higher in herbivorous freshwater fish than in others. However, the concentration of Pb is mostly in omnivores that reside in seawater.

**Table 1:** Heavy metal(loid)s concentration (µg g^−1^ fresh weight) in Bangladeshi fishes based on habitat, such as freshwater, seawater, and euryhaline fishes

Metal(oid)s	As	Cd	Cr	Cu	Hg	Mn	Ni	Pb	Zn
Fish Type	Freshwater								
No. of data point	62	57	62	57	47	45	47	64	50
Minimum	0.0006	0.0002	0.0001	0.0013	0.0001	0.003	0.0006	0.0005	0.0096
Maximum	1.08	0.80	8.78	18.6	0.75	22.8	9.89	7.16	182.40
Mean	0.17	0.09	1.25	2.60	0.09	3.84	0.89	0.95	27.90
Standard deviation	0.23	0.16	1.87	3.14	0.15	6.02	1.61	1.27	38.50
Median	0.10	0.03	0.54	1.30	0.03	1.18	0.43	0.59	14.20
Fish Type	Saltwater								
No. of data point	28	25	29	27	15	22	17	29	26
Minimum	0.002	0.001	0.01	0.055	0.00004	0.007	0.011	0.0002	0.898
Maximum	3.53	14.10	3.61	6.24	8.13	11.50	6.13	8.92	31.34
Mean	0.50	1.00	0.63	1.39	0.90	1.05	0.45	1.14	7.40
Standard deviation	0.87	3.01	0.83	1.76	2.37	2.33	1.37	2.10	7.12
Median	0.05	0.07	0.34	0.61	0.07	0.29	0.03	0.07	6.42
Fish Type	Euryhaline								
No. of data point	9	9	10	9	7	8	9	10	10
Minimum	0.008	0.0005	0.002	0.02	0.003	0.017	0.003	0.0009	0.26
Maximum	1.10	0.43	4.17	4.30	0.78	9.54	1.47	3.95	67.54
Mean	0.35	0.11	0.98	1.29	0.14	1.62	0.46	1.19	12.26
Standard deviation	0.39	0.14	1.29	1.43	0.29	3.26	0.46	1.46	20.44
Median	0.20	0.05	0.59	0.45	0.02	0.23	0.48	0.63	5.03
Maximum Permissible Limit [59]	1	0.05	1	30	0.1	1	80	0.3	30

**Table 2:** Heavy metal(loid)s concentration (µg g^−1^ fresh weight) in Bangladeshi fishes based on feeding behaviour, namely, omnivorous, carnivorous, and herbivorous fishes

Metal(oid)s		As	Cd	Cr	Cu	Hg	Mn	Ni	Pb	Zn
Fish Type	Omnivorous									
No. of data point		42	40	43	39	31	31	30	44	37
Minimum		0.005	0.001	0.006	0.06	0.0002	0.03	0.006	0.01	0.55
Maximum		2.07	1.52	7.91	18.62	0.75	20.95	9.90	7.16	136.87
Mean		0.24	0.14	1.28	2.55	0.11	2.96	1.10	1.01	24.32
Standard deviation		0.39	0.30	1.86	3.37	0.18	4.71	1.96	1.42	30.24
Median		0.07	0.02	0.60	1.24	0.05	1.09	0.40	0.63	12.70
Fish Type	Carnivorous									
No. of data point		42	38	43	38	26	30	31	44	35
Minimum		0.0006	0.0002	0.01	0.06	0.00001	0.02	0.01	0.0002	0.40
Maximum		2.04	14.10	8.78	6.43	8.13	16.97	1.54	8.92	71.79
Mean		0.29	0.64	0.83	1.47	0.80	1.61	0.42	1.04	11.02
Standard deviation		0.39	2.46	1.46	1.64	2.18	3.14	0.50	1.60	13.82
Median		0.12	0.07	0.38	0.98	0.06	0.70	0.12	0.62	7.55
Fish Type	Herbivorous									
No. of data point		16	14	16	15	11	13	13	16	14
Minimum		0.0006	0.0005	0.0001	0.001	0.0001	0.003	0.0006	0.0005	0.01
Maximum		0.90	0.21	6.01	9.91	0.29	22.82	1.33	3.90	182.35
Mean		0.18	0.05	1.00	2.86	0.07	5.13	0.39	0.68	32.40
Standard deviation		0.25	0.06	1.53	2.64	0.10	7.94	0.44	1.07	55.39
Median		0.10	0.03	0.44	1.91	0.01	1.12	0.44	0.41	12.37
Maximum Permissible Limit (MPL) [59]		1	0.05	1	30	0.1	1	80	0.3	30

In contrast, comparing the mean concentrations of As, Cu, Ni, and Zn in all the classifications with the maximum permissible limits (MPLs), their concentrations were lower than the respective MPL values. Whilst Mn and Pb content in the fish types was found to be higher than the MPL value of 1 and 0.3 μg g^−1^, respectively, the average content of three potent elements (Cd, Cr, and Hg) in herbivorous fishes and Hg content in omnivores were close to their respective allowable concentrations. Conversely, Cd, Cr, and Hg concentrations in the carnivores and the seawater fishes were lower than in the respective MPLs. This result also continues for Hg content in the freshwater fishes of Bangladesh. The rest of the fish types showed higher values of Cd, Cr, and Hg than the respective MPLs (Tables 1 and 2).

### PCA Results

**Fig. 1:**
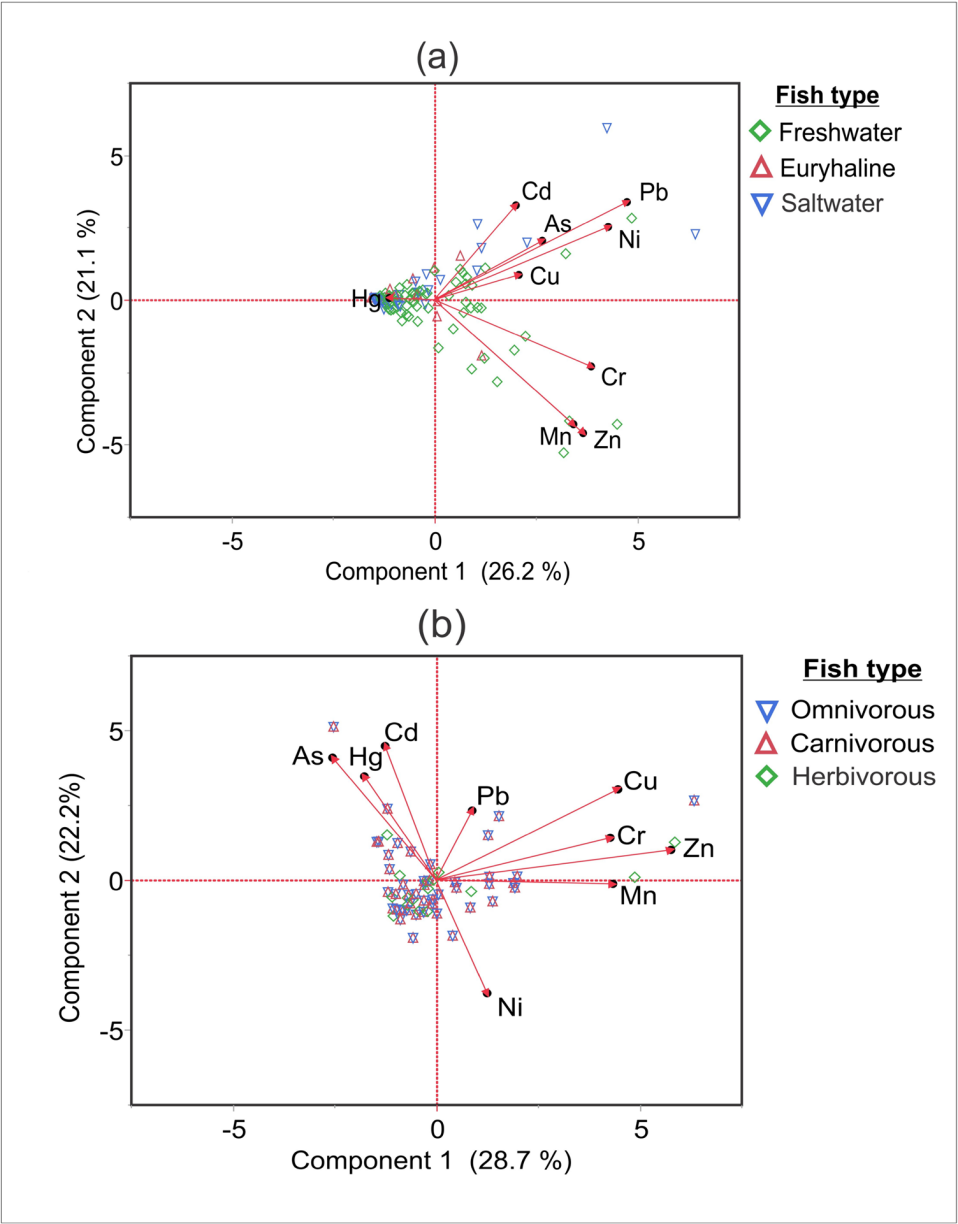
Principal Component Analysis (PCA) explains heavy metal(loid)s for different types of categorised fishes available in Bangladesh; (a) Fish from various sources (Freshwater, Euryhaline, and Seawater), and (b) the fish types (Omnivorous, Carnivorous, and Herbivorous)

In the context of fish populations categorized by habitat (freshwater, seawater, and euryhaline), the initial factor accounted for 26.2% of data variance, while the subsequent factor explained 21.1% of the data variability. The first factor exhibited a strong positive correlation with metal(loid)s such as Cd, Pb, Ni, As, and Cu within cluster 1. Conversely, cluster 2 displayed notable resemblances in the relationship of Cr, Mn, and Zn according to PCA ordination diagrams (Fig. 1a). Besides, fish populations were categorized by food habits (Carnivorous, Herbivorous, and Omnivorous), the primary factor accounted for 28.7% of data variance, while the subsequent factor elucidated 22.2% of data variability. Cluster 1 exhibited a strong positive correlation with metal(loid)s such as, As, Hg, and Cd. Notably, cluster 2 represented remarkable resemblances in the relationship among Pb, Cu, Cr, Zn, and Mn, as indicated by PCA ordination diagrams (Fig. 1b).

### Human Risk from Fish Consumption

Human health risks from metal(loid) exposure through fish consumption were estimated using the US EPA model. Both cancer and non-cancer health risks were estimated in terms of carcinogenic risk (CR), and target hazard quotient (THQ), respectively.

**Fig. 2:**
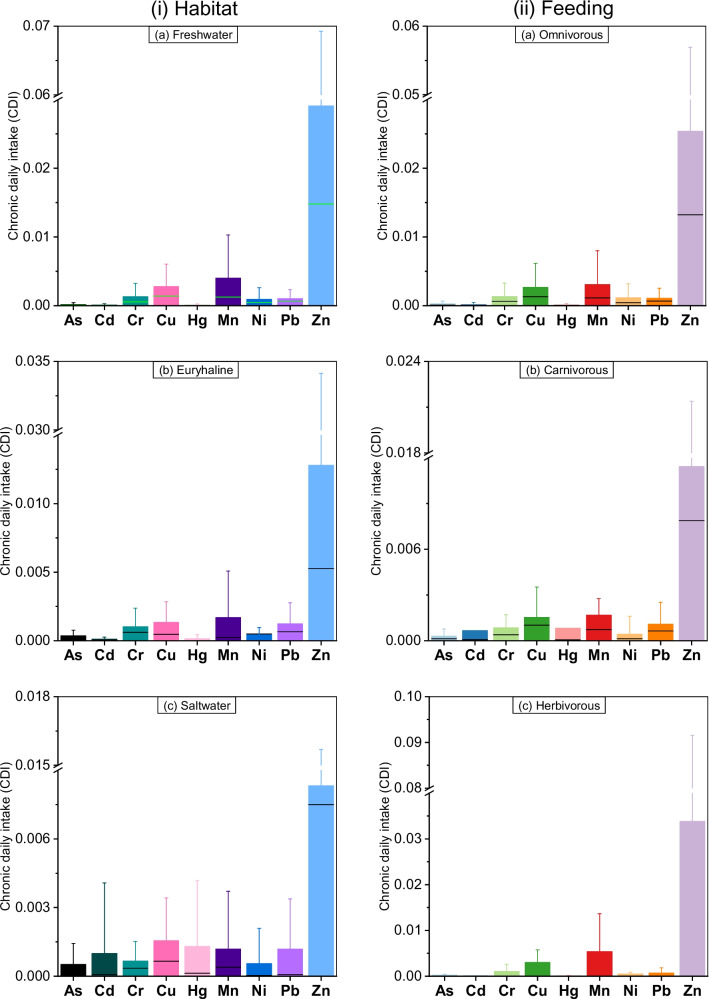
Chronic daily intake (CDI) of heavy metals from consumption of different types of fish (left and right panels indicate fish types based on habitat and feeding behaviour, respectively)

**Fig. 3:**
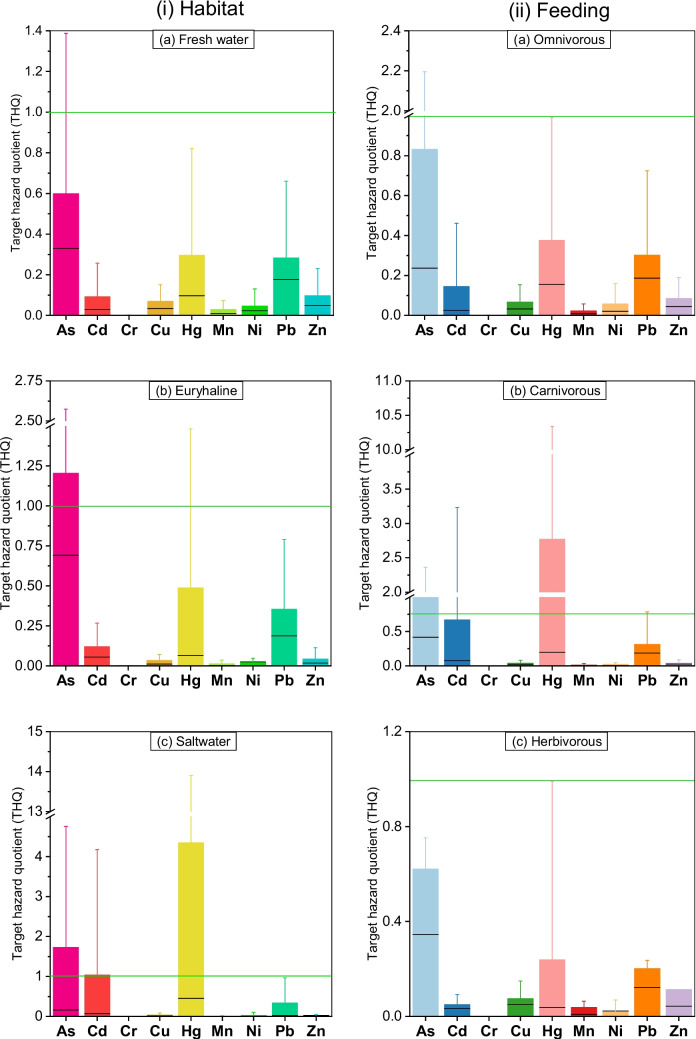
Non-cancer risks based on target hazard quotient (THQ) of heavy metals from consumption of different types of fish (left and right panels indicate fish types based on habitat and feeding behaviour, respectively)

The non-carcinogenic heavy metal(loid) risk factors for humans from different types of fish ingestion in Bangladesh are displayed in Fig. 2 and Fig. 3. The CDI values for the freshwater fish and the omnivores shown in Fig. 2 and Table S2 followed the same order. For freshwater and omnivores, the decreasing order of CDI was as follows: Zn > Mn > Cu > Cr > Pb > Ni > As > Cd > Hg. For saltwater fishes, the order was Zn > Cu > Hg > Mn > Pb > Cd > Cr > Ni > As where the euryhaline water, the order was as follows: Zn > Mn > Cu > Pb > Cr > Ni > As > Hg > Cd. Also, for carnivores, the order was Zn > Mn > Cu > Pb > Cr > Hg > Cd > Ni > As. Finally, for herbivores, the CDI order was as follows: Zn > Mn > Cu > Cr > Pb > Ni > As > Hg > Cd. The daily intake of Zn was significantly higher than other metal(loid)s across different fish types.

Figure 3 and Table S3 show the THQ of different types of fish considered in this study. The THQ values for the freshwater and the omnivorous fish followed the order of As > Hg > Zn > Cd > Cu > Ni > Mn > Pb > Cr, and As > Hg > Cd > Zn > Cu > Ni > Pb > Mn > Cr, respectively. Whereas, the seawater and the carnivorous fish portrayed the order of Hg > As > Cd > Cu > Pb > Zn > Ni > Mn > Cr, and Hg > As > Cd > Cu > Zn > Pb > Ni > Mn > Cr, accordingly. THQ values of heavy metal(oid)s are rated in the following order of As > Hg > Cd > Zn > Pb > Cu > Ni > Mn > Cr for euryhaline fish; As > Hg > Zn > Cu > Cd > Mn > Ni > Pb > Cr for herbivores. THQ > 1 indicates a chance of non-carcinogenic health risk, while THQ ≤ 1 indicates no possible health risk [37]. Among metal(loid)s, Hg showed the highest THQ value of 4.35 for seawater fish, which is almost four times higher than the threshold value of 1 (Fig. 3). The second highest THQ was also observed for Hg in carnivores (THQ = 2.77), while As in seawater, euryhaline, and carnivorous fish showed THQ values of 1.73, 1.20, and 0.995, respectively. Cd in seawater fishes also showed a THQ value over the threshold limit of 1 (Fig. 3i). Consequently, consumption of As, Hg, and Cd through seawater, euryhaline, and carnivorous fishes is likely to pose non-carcinogenic health risks to Bangladeshi inhabitants. However, consumption of fish for other metal(loid)s does not pose any non-cancer health risks to Bangladeshi residents as they showed a THQ value of less than 1. The cumulative THQ value of all metal(oid)s indicated the following order seawater (7.58) > euryhaline (2.28) > freshwater (1.51) (Fig. 3i) and carnivorous (4.86) > omnivorous (1.89) > herbivorous (1.36) (Fig. 3ii).

**Fig. 4:**
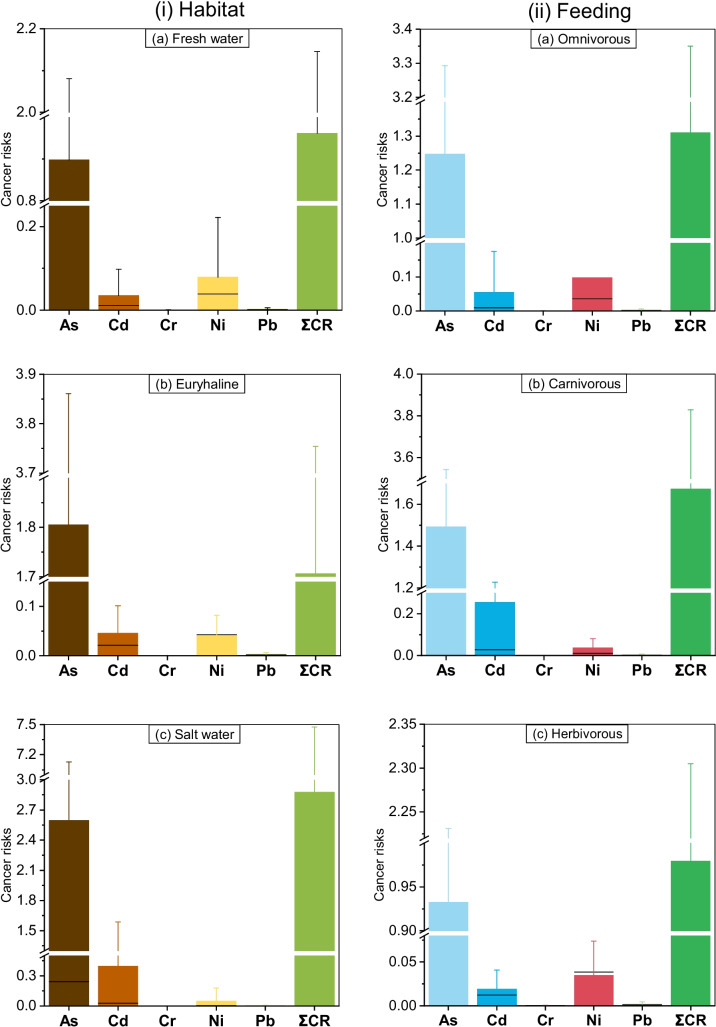
Carcinogenic risks (CR) of heavy metals from consumption of different types of fish (left and right panels indicate fish types based on habitat and feeding behavior, respectively)

Lifetime cancer risks were also estimated, and the cancer risks of As, Cd, Cr, Ni, and Pb in various fish types are provided in Fig. 4 and Table S4. As, Cd, and Ni showed a higher value for all the fish categories than the highest threshold limit of 10^–4^. In addition, Pb portrayed a higher value of CR than the threshold limit of 10^–4^. In contrast, CR of Cd and Ni were also above the permissible limit except for measurements taken from seawater and the herbivorous fish. This result indicates that fish consumption poses a cancer risk to the population. However, a maximum CR value of 1.96 × 10^–3^ was delineated by Ni in omnivores, which is about 20 times higher than the safe limit of 10^–4^. The cumulative CR value of all metal(loid)s indicated the following order seawater (2.88 × 10^0^) > euryhaline (1.71 × 10^0^) > freshwater (9.62 × 10^–1^) (Fig. 4i) and carnivorous (1.67 × 10^0^) > omnivorous (1.31 × 10^0^) > herbivorous (9.79 × 10^–1^) (Fig. 4ii). Most of the metal(oid)s showed a higher level of CR values, indicating a higher possibility of cancer risks to the Bangladeshi residents who consume various types of fish.

### Computer Model-Based Approach and Possible Guidelines

**Fig. 5:**
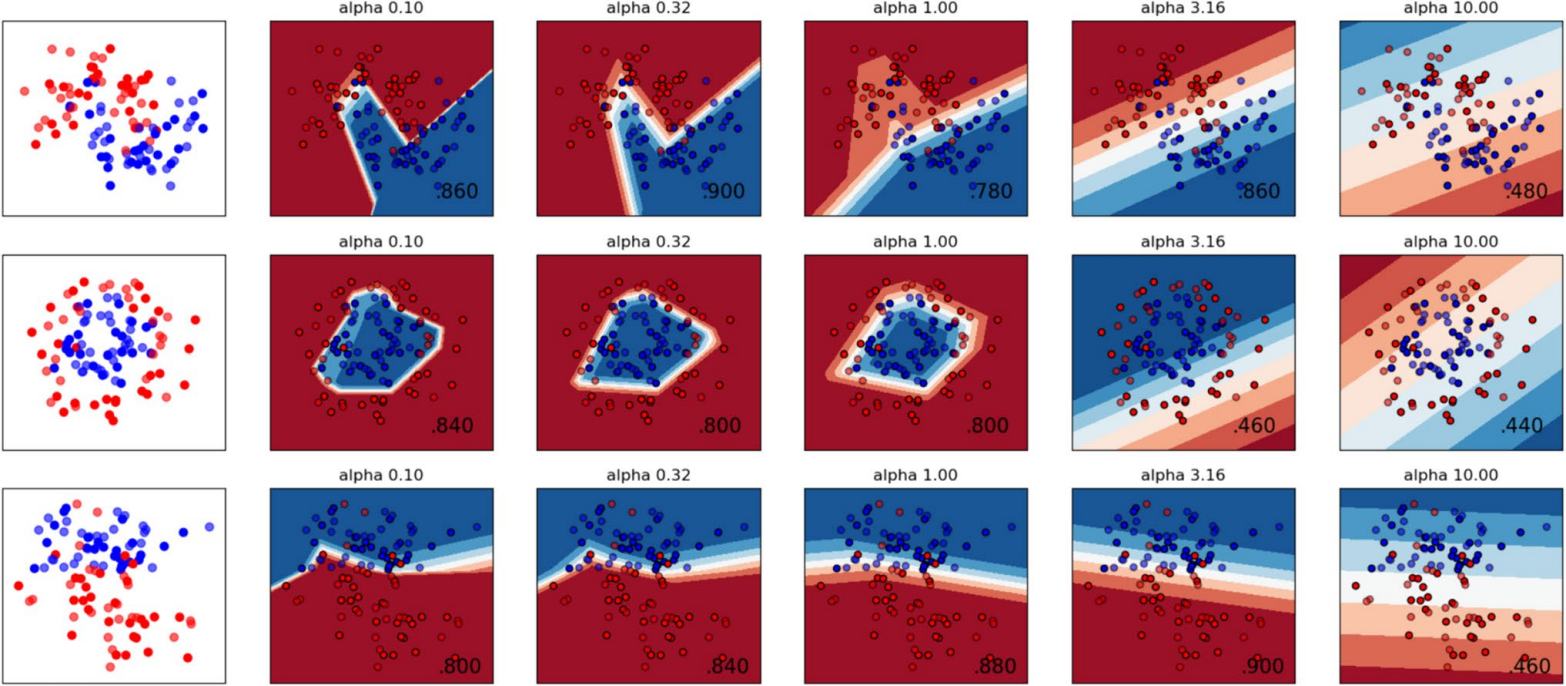
Expression of the neural model with evaluating α-values and data distribution and clustering defining test conditions and significance level

The model results display potential daily consumption levels, considering the contamination status of metal(loid)s and the accepted concentration of carcinogens with no associated risks. The THQ = 1 value can be employed to compute a potential safety threshold for consumption. Necessary information for the categorical data and numeric values using Noncomparative Numerical Notation to decode fish species are provided in Tables S5 and S6. The independent variables were utilised to synthesise three different synthetic variables employed for testing relationships among the variables and ɑ distribution value later in the process. The ɑ value shows the cluster nature of the classified data. It ensures the accuracy of the clustering process and expresses a clearly distinguished hidden class among the datasets. Among 3 individual sub-groups, a 90% confidence interval (CI) was observed for α 0.32 in cluster 1 and α 0.316 in cluster 3, and even 88% CI was observed at α 1.0. Based on ANN and every independent variable and constructed 3 individual sub-groups, the data was used for further calculations (Fig. 5). The SHAP values and their influence over the output have been categorized and tested for sample size separately with 90% and 10%, respectively. SHAP values exert a positive influence on predictions when they are positive values, however, a negative influence when they are negative values. The magnitude of these values serves as an indicator of the strength of their impact. 90% of tested samples and 10% of tested samples did not show significant changes in the contribution of a feature (Fig. S2). This indicates that SHAP values offer a dependable explanation of the model's actions or performance which was applied to evaluate the safe consumption limit (Fig.S3).

**Fig. 6:**
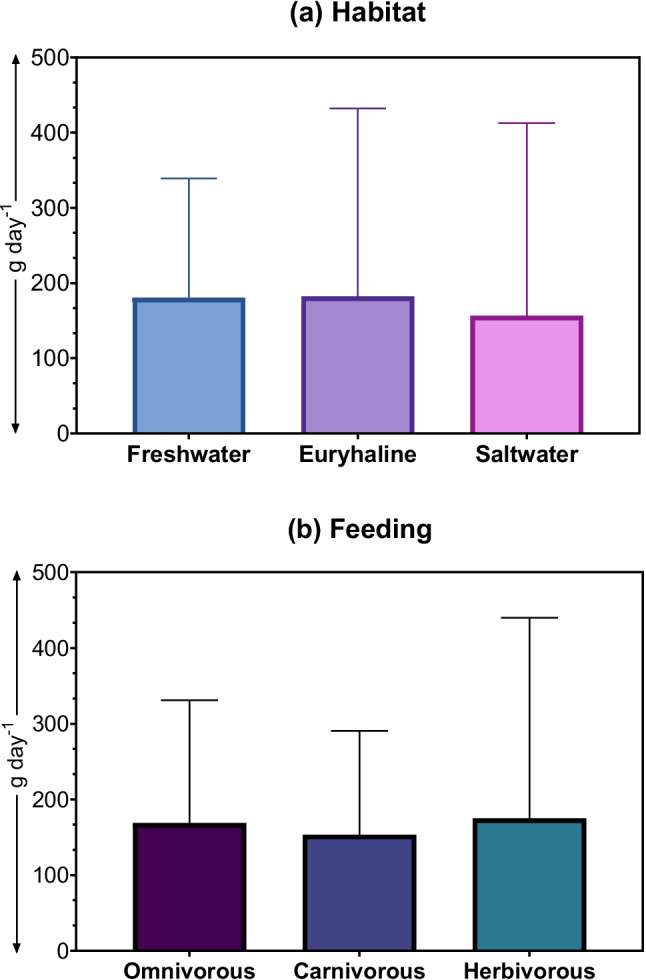
Status of the safest fish consumption (**a**) Fish habitat and (**b**) Feeding behavior based on collected data and analysis of the model, ANN

The major force driven to the safe consumption limit was metal(loid)s based, As > Zn > Hg > Pb > Cd > Cu > Cr > Mn > Ni (Table S6). The higher THQ value and frequency both were considered when determining their contribution to identifying the safe consumption limit. Safe consumption limits for every single species are provided in Table S7 and Table S8. The average safe consumption of the freshwater fishes was 180.59 g day^−1^, while 182.17 g day^−1^ and 156.51 g day^−1^ were the average safe limits for the euryhaline and saltwater species, respectively (Fig. 6; Table S7). On the other hand, the average safe limit for omnivores was 168.63 g day^−1^, whereas the 153.05 g day^−1^ and 175.09 g day^−1^ were for carnivorous and herbivorous, respectively (Fig. 6; Table S8).

## Discussion

### Heavy Metal(Oid)s and their Bioaccumulation in Fish Categories

Heavy metal(loid)s such as Chromium (Cr), Copper (Cu), Manganese (Mn), Nickel (Ni), Arsenic (As), Lead (Pb) and Zinc (Zn) were found in freshwater fishes. Among these metal(loid)s, Cr content exceeded the reference values in approximately one-third of the freshwater and omnivorous fishes. The higher concentration of Cr in omnivores, like *Heteropneustes fossilis* (Stinging catfish), was attributed to the discharge of chromium tanning liquor from nearby tannery industries situated along the banks of Buriganga and Turag, two urban rivers in the country. This discharge of industrial waste into the water bodies was suggested as the possible reason for increased Cr in these fishes [13]. Similarly, another study found a considerable level of Cu and Zn in the freshwater fish *Mystus vittatus* (Striped dwarf catfish). It was revealed that ingesting food with metal(loid)s in the water of the Buriganga river during the winter season [45]. The presence of these metal(loid)s in the fish was attributed to water pollution. Further studies were conducted on the Buriganga River and Turag River fishes, where a higher concentration of Mn was found than other metals in the Buriganga River. This higher concentration of Mn was attributed to both natural and anthropogenic sources, including the release of agrochemicals into the water bodies through runoff or leaching. However, the highest mean concentration of Ni was found in a freshwater fish called *Heteropneustes fossilis* (Stinging catfish) in the Turag River [2, 13].

The euryhaline fish can live in both freshwater and seawater. Cu, Ni, and Pb concentrations in the euryhaline fishes were more similar to fish living in seawater. These heavy metals showed similarities between euryhaline and seawater fishes. Due to their exposure to pollution levels from both sources, their metal concentrations were found to be intermediate between freshwater and seawater fishes. Euryhaline fish are more tolerant of a wide range of salinity naturally derived from seawater [46]. Regarding Pb concentration, the highest level was found at 202 times higher than the permissible limit in a euryhaline and herbivorous fish called *Liza parsia* (Goldspot mullet) from the Halda River [47, 48]. It was noted that more than half of the collected data from omnivores also showed higher Pb content. Another study found that the omnivore, *Puntius ticto* (Ticto barb), had the highest accumulation capacities for Pb and Cd [49]. Omnivorous fish, which consume a diverse diet, including other fish, zooplankton, and plant-based foods, were found to have higher concentrations of Mn, Zn, and Cr compared to other fish types. However, except for Mn, Pb, and Zn, the mean concentration of heavy metals in all fish types remained below permissible values. In another study conducted by Ahmed et al. [2] it was observed that the herbivorous fish species, *Labeo Rohita* (Rohu), had Mn and Zn concentrations exceeding acceptable limits by 125-fold and 8-fold, respectively. This implies a potential link between agricultural activities and the presence of effluents in rivers.

On the other hand, the carnivorous fish species showed higher levels of Cd and Hg, however, the mean concentration of “As” was within safe limits in all fish types. Nonetheless, around two-thirds of the data collected from carnivores exceeded the safe limit for Pb. Notably, the carnivore, *Channa striata* (Striped snakehead) fish exhibited the highest Pb content, surpassing the safe limit, as reported in a study on the Turag River by Hossain et al. [13]. The “As” concentration was higher in seawater fishes and more than twice the permissible level in a carnivorous marine fish, *Sillaginopsis panijus* (Flathead sillago) [50]. Another study showed that saltwater and carnivorous fishes like *Harpadon nehereus* (Bombay duck), *Trichiurus lepturus* (Largehead hairtail), and *Panna microdon* (Panna croaker) surpassed the acceptable levels of Hg by more than 60, 101, 81, and 78-fold, respectively [51]. The Cd concentration in marine and omnivorous fish, *Rastrelliger kanagurta* (Indian mackerel), was 30 times higher than the acceptable limit [14]. However, the carnivorous *Halichoeres nigrescens* (Bubble fin wrasse), exhibited the highest Cd content of more than 250 times than the safe limit in his study. Therefore, “Cd” occurs naturally at low levels in the environment, so the concentration of “Cd” in the aquatic environment may increase by industrial processes, such as smelting or electroplating and fertilizers [14]. However, the enrichment of anthropogenically fueled heavy metals in seawater fishes of Bangladesh might be due to the downstream location of sampling points of the study areas, as the seawater fishes of Bangladesh are mostly caught in Chittagong, the industrial capital with the country’s biggest seaport. Overall, the safe consumption of fish depends on pollution sources, habitat differences, feeding behavior, fish and metal specificity, and bioconcentration factors. Further studies should focus on analyzing the spatial variability of heavy metal(loid)s contamination among different fish types available in Bangladesh.

### Relationship Among Heavy Metal(Loid)s

Examining fish populations grouped by habitat (freshwater, seawater, and euryhaline), the initial factor explained 26.2% of the data's variability, with the subsequent factor clarifying 21.1% of the variance. The first factor displayed a robust positive connection with metal(loid)s like Cd, Pb, Ni, As, and Cu within cluster 1. In contrast, cluster 2 exhibited distinct parallels in the correlation between Cr, Mn, and Zn, as highlighted by PCA ordination diagrams. This assessment revealed two principal clusters that showcased analogous patterns of metal(loid)s and essential metals in fish (Fig. 1a). When examining the grouping of metal(loid)s in fish based on feeding habits (omnivorous, carnivorous, and herbivorous), two noteworthy clusters emerged, revealing shared tendencies in metal(loid)s and essential metal accumulation among fish, except for Ni, which was assigned to distinct components. The primary factor accounted for 28.7% of data variability, while the ensuing factor illuminated 22.2% of the variability. Cluster 1 displayed a robust positive correlation with metal(loid)s such as As, Hg, and Cd. Importantly, cluster 2 demonstrated striking resemblances in the interrelationship of Pb, Cu, Cr, Zn, and Mn, as depicted by PCA ordination diagrams (Fig. 1b).

### Lifetime Health Risk

The human health risk estimations encompassed both non-cancer and cancer risks, quantified through target hazard quotient (THQ) and carcinogenic risk (CR), respectively. Additionally, to determine these indices, chronic daily intake (CDI) of metal(loid)s was calculated. The CDI of metals from fish consumption indicated a relatively higher intake of Zn, Mn and Cu, regardless of fish type. Contrarily, a lower intake of As and Cd from fish was observed. This could be attributed to the variability of the concentration of metal(loid)s in fishes, given that other parameters, such as body weight, fish intake rate, etc., were kept constant in CDI calculations. The CDI provides valuable information on the intake rate of different metal(loid)s present in the fish and facilitates THQ and CR calculations. Besides, CDI indicated the highest metal(loid)s intake rate was from freshwater and herbivorous fishes in Bangladesh. It was found that feeding habits and living habitats significantly influence metal(loid)s bioaccumulation in fish [52]. For freshwater fishes, the higher metal(loid)s contamination might be due to the metal pollution in aquaculture as most of the freshwater fishes are cultivated in ponds/lagoons in Bangladesh. Possibly, the use of groundwater, pesticides, insecticides, fertilizer, and application of synthetic foods contributed to higher metal(loid)s accumulation in freshwater fishes. It was reported that fish feed in Bangladesh contains higher levels of Pb, Cu, Zn and Mn [60]. Thus a significant amount of heavy metal(oid) is bioaccumulated in fish muscle from the fish feed [63]. However, the bioaccumulation of metal(loid)s depends on the salinity, ecological and metabolic activities, the gradients of contamination source, and temperature [53, 54]. For example, there were significant differences in Hg concentration in freshwater and saltwater fishes due to the difference in salinity and bioavailability of Hg connected to higher bacterial activity in brackish water [55]. Again, omnivorous fishes consume a wide variety of foods, which could explain their higher metal contamination and subsequently elevated CDI values. It was speculated that omnivorous fishes may bioaccumulate more heavy metal(loid)s than carnivorous fish in natural habitats [56].

In general, a THQ value exceeding 1 indicates a potential for non-carcinogenic health risk, whereas THQ ≤ 1 suggests no significant health risk [57]. Among all metal(loid)s, Hg exhibited the highest THQ value of 4.35 for seawater fish consumption, exceeding the threshold by more than fourfold. The second-highest THQ was observed for Hg in carnivores (THQ = 2.77), while As in seawater, euryhaline, and carnivorous fish displayed THQ values of 1.73, 1.20, and 0.995, respectively. The Cd in seawater fish also surpassed the threshold limit of 1. Consequently, the consumption of seawater, euryhaline, and carnivorous fish potentially appears to pose non-carcinogenic health risks to the Bangladeshi population arising from As, Hg, and Cd. Across all fish categories (e.g., freshwater, euryhaline, and saltwater) that are contaminated with As, Cd, Cr, and Pb, posed non-cancer risks to Bangladeshi inhabitants [62]. Interestingly, the intake of As, Hg, and Cd was lower, however, the non-cancer risks associated with them are higher as these elements are toxic at even very low concentrations. Also, the net non-cancer risks are higher for seawater and carnivorous fishes in Bangladesh, though the metal(oid)s consumption was higher for freshwater and herbivorous fishes. However, the consumption of other individual metal(oid)s through fish consumption seemingly does not entail any non-cancer health risks to Bangladeshi residents, as their THQ values remain below 1.

Across all classifications of fishes, As, Cr, and Ni exhibited CR values surpassing the upper threshold limit of 10^–4^. The cancer risk of Cd was within permissible limits for most of the fish types, except for the seawater and carnivorous fishes. The highest CR value was 1.96 × 10^–4^ was found for Ni due to omnivore fish consumption. The cancer risk was approximately 20 times compared to the maximum safe limit of 10^–4^. The cumulative cancer risks indicated lifetime cancer probabilities from the consumption of all types of fishes contaminated with As, Cd, Cr, Ni, and Pb, where the highest risks are posed by seawater and carnivorous fishes. Possibly, higher toxicity (as speculated from low RfDo and high slope factor) of these elements contributed to higher non-cancer and cancer risks from the consumption of seawater and carnivorous fishes in Bangladesh. A recent study also found that consumption of freshwater, euryhaline, and seawater fishes contaminated with As, Cd, Cr and Pb pose cancer risks for Bangladeshi inhabitants [62].

### Computer Model-Based Outcomes and Possible Guidelines for Safety Consumption

According to the evaluation by computational modeling, in the habitat group, the safest fish to consume is freshwater fish, and the most harmful is saltwater fish. Literature also suggests that farmed freshwater fishes were safe to consume as the THQ and CR values for both adults and children were within acceptable bounds [60]. The reason behind this difference in risks could be the contamination of seawater with metal(loid)s through either continuous contamination of shoreline, as rivers flush into the sea during the rainy season, daily reinforced contamination through nearby urban and industrial activities, or by fish-specific bioaccumulation of individual metal(oid)s or salinity influences heavy metal(loid)s bioaccumulation, or metal-specific bioaccumulation by fishes.

It is well established that a larger portion of Bangladeshi coastal areas are contaminated with metal(loid)s. In coastal areas, it was reported that concentrations of Co, Cd, Pb, Cu, Cr, Mn, Fe, and Ni in water, sediment, and fish were relatively higher than their respective permissible limits, especially in the ship-breaking areas, where the Cr levels in water and sediment were 86.93 mg L^−1^ and 55 mg kg^−1^, respectively [61]. The localized contamination in coastal areas might be the major contributing factor to metal(loid) contamination in saltwater fish.

Different fishes have different ion regulatory processes in their physiology. Freshwater fishes actively take dissolved ions (i.e., metal ions) through gills, whereas saltwater fishes regulate ion balance through gills and drink water to maintain osmoregulatory processes [65]. The additional metal exposure pathway through the intestine might have resulted in higher bioaccumulation of metal(loid)s in saltwater than in freshwater fishes. So, there were greater health risks and stricter safe consumption limits for saltwater fish.

The present research evaluated that saltwater fish are recommended to lower average consumption at 156.51 g day^−1^ while freshwater and euryhaline fishes are recommended to 180.59 and 182.17 g day^−1^, respectively, although fish-specific allowable limits are different (Table S7). It expressed that the salinity difference may play a crucial role in metal(loid) bioaccumulation, which alters the safe consumption limit of fish. For example, *Glossogobius giuris* (Tank goby) is found in both freshwater and euryhaline areas. The safe consumption of *G. giuris* for euryhaline was 137.4 g day^−1^ for an adult with 60 kg of body weight, while the same freshwater fish was recommended for 327.2 g day^−1^. Further, a study found that Cd accumulation in the liver of Tilapia fish, *Oreochromis mossambicus* was significantly increased after 10 days of exposure at both 0.5 mg L^−1^ Cd and 1.5 mg L^−1^ [58]. Following the Cd exposure period, Cd accumulation levels in the liver ranged from 121.03 to 128.83 mg kg^−1^ at 1.5 mg L^−1^ across various salinity levels, marking a three to four-fold increase compared to exposure at 0.5 mg L^−1^ of Cd. It demonstrated that the water salinity significantly impacts the bioaccumulation of metal(loid)s in fish.

The feeding behaviour of fish also influences heavy metal(oid) bioaccumulation in the body. This study revealed that herbivores are the safest fish for human consumption, while omnivorous fish carry the highest risk. The increased safest limit of fish consumption for the herbivorous fish, which are less contaminated and allowed to consume 175.09 g day^-1^; however, the carnivorous and omnivorous fish allowable intake is 153.05 and 168.63 g day^-1^, respectively. For example, *Labeo bata* (Bata) is a herbivore, and the safe consumption of this species was predicted to be 1136.53 g day^−1^ for an adult with 60 kg of body weight. Conversely, *Clarias batrachus* (Walking catfish) and *Mystus gulio* (Long whiskers catfish) are omnivorous fish that showed daily safe consumption of 87.8 and 63.33 g day^−1^, respectively, for a 60 kg body-weighted adult person because the omnivorous fish species consume smaller fish with other organisms such as plankton and aquatic plants which have already contaminated with heavy metal(loid)s, leading to higher metal(loid)s bioaccumulation.

Differences in heavy metal(loid)s and their specific bioaccumulation nature can influence the health risk and daily safe intake. In Gangetic hairfin anchovy (*Setipinna phasa*), As (0.017 mg kg^−1^ fresh weight) and Hg (0.011 mg kg^−1^ fresh weight) concentrations were almost the same, but “As” posed less health risk than Hg [64] because the redox potential and valence state of individual metal(loid)s as well as the pH, organic matter, hardness, and ionic strength of the aquatic medium affect metal(loid) bioaccumulation in fishes [66]. For instance, methylmercury (MeHg) is more bioavailable and toxic than inorganic (Hg^0^) and divalent mercury (Hg^2+^) [67]. It was found that the higher organic content significantly increased Hg bioaccumulation, the low pH increased Cd bioavailability, and the low salinity increased Pb bioaccumulation in fishes [68].

## Conclusion

This study offers a comprehensive insight into the pollution stemming from metal(loid)s and the potential health hazards linked to their presence in fish consumed by Bangladeshi inhabitants. Significant variations in metal(loid) concentrations were observed among fish types, attributable to disparities in their internal biochemistry and the extent of human-induced pressures. Additionally, seawater and carnivorous fishes exhibited the highest non-cancer risk, while freshwater and herbivorous fishes presented the lowest. Despite Hg and As displaying increased THQ values across various fish types, the calculated THQ values for all heavy metal(loid)s in freshwater, omnivore, and carnivore fishes remained below 1, suggesting no to minimal health risks to the consumers. For each fish type, there is a high potential for cancer risk from fish consumption, mostly contributed by As, Cr, and Ni.

Computational modeling through the ANN & DTR approach was employed to measure safe consumption levels of various fish types considering human health concerns. Based on habitat, the safest fishes to consume are freshwater communities, while the most potentially harmful fishes are those from marine water. Also, based on feeding behavior, it was observed that herbivorous fish are generally safer for consumption, whereas omnivorous and carnivorous fish tend to be more harmful.

Overall, it was determined that the significant input of heavy metal(loid)s pollutants into rivers from nearby urban and industrial zones, as well as pollution originating from shipbreaking industries and other human activities in marine ecosystems near the shoreline, has had a crucial impact on the health risks linked to the consumption of fish. Besides the habitat and feeding behaviour of fishes, differences in salinity, organic matter, pH of the aquatic environment, and metal(loid) biochemistry affect bioaccumulation. Hence, human health risks and safe consumption limits varied among fishes. Therefore, it is essential to promptly implement rigorous monitoring and act on waste purification before discharging, which could reduce the contamination of Bangladeshi fish with metal(loid)s. Finally, this research strongly recommends further monitoring while considering all relevant co-factors (e.g., location, species, habitat, feeding behaviour, age, and size) related to metal(loid) bioaccumulation in fishes.

## Supplementary Information

Below is the link to the electronic supplementary material.

Supplementary file1 (DOCX 499 KB)

Supplementary file2 (DOCX 15141 KB)

## Data Availability

Data are available in the manuscript and in the supplementary materials.
