# Supplementary material for: Exploring Potential Human Health Risks Linked to Heavy Metal(Loid)s in Dietary Fishes: Utilizing Data-Driven and Computational Modelling Approaches
Source: Biol Trace Elem Res. 2024 Sep 11;203(5):2830–47. doi: 10.1007/s12011-024-04363-6 (PMC12125157; doi:10.1007/s12011-024-04363-6)
Supplement: Supplementary file 1 — Supplementary file1 (DOCX 499 KB) [file 12011_2024_4363_MOESM1_ESM.docx]

*Supplementary information for*

**Exploring potential human health risks linked to heavy metal(loid)s in dietary fishes: Utilizing data-driven and computational modelling approaches**

Pritom Bhowmik Akash^1^, Sazal Kumar^2^, Md. Saikoth Jahan^3^, Muhammad Shafiqur Rahman^4^, Md. Assraf Seddiky^5^, Anti Sorker^6^, and Rafiquel Islam^2,7*^

*^1^Department of Civil Engineering, Chittagong University of Engineering & Technology, Chatttogram, 4349, Bangladesh*

*^2^School of Environmental and Life Sciences, The University of Newcastle (UoN), Callaghan, NSW 2308, Australia*

*^3^Department of Geography and Environment, Islamic University, Kushtia 7003, Bangladesh*

*^4^Materials and Chemical Processing Laboratory, The University of Adelaide, Adelaide SA 5000, Australia*

*^5^Department of Public Administration, Shahjalal University of Science & Technology, Sylhet 3114, Bangladesh*

*^6^Department of Agricultural Economics, Faculty of Agribusiness Management, Sher-e-Bangla Agricultural University, Sher-e-Bangla Nagar, Dhaka 1207, Bangladesh*

*^7^Department of Applied Chemistry and Chemical Engineering, Islamic University, Kushtia 7003, Bangladesh*

*^*^****Corresponding Author:*** Dr. Rafiquel Islam; Email: [rafiquel.islam@newcastle.edu.au](mailto:rafiquel.islam@newcastle.edu.au)

**Supplementary Table S1**: A PRISMA flow diagram for selecting articles for final review after obtaining articles from searching databases.

| **Steps** | **Domain and approach** | **Total articles** | **Included/Excluded** |
| --- | --- | --- | --- |
| Search | Domain: All field  Approach: Thematic | Total = 426 | N = 426 |
| Screening: | Domain: Type of article, duplicates and language  Approach: Only research articles that are written in English | N = 426 | Included: 214  Excluded: 212 |
| Selection  and  Exclusion | Domain: Article title, abstract, and keywords  Approach: Exclude articles that do not report metals (e.g., As, Cd, Cr, Cu, Hg, Mn, Ni, Pb, and Zn) in fish | N = 214 | Included: 76  Excluded: 138 |
|  | Domain: whole manuscript  Approach: Include those articles that reported As, Cd, Cr, Cu, Hg, Mn, Ni, Pb, and Zn in fish in the table, text, and supplementary data | N = 76 | Included: 46  Excluded: 30 |
| Included for data collection | Articles included for final data curation | N = 46 |  |

**Supplementary Table S2**: Chronic daily intake (CDI) [µg g^-1^ fresh weight person^-1^ day^-1^] of heavy metal(oid)s from different types of fish intake by the Bangladeshi population.

|  | **N total** | **Mean** | **Standard Deviation** | **Median** | **Minimum** | **Maximum** |
| --- | --- | --- | --- | --- | --- | --- |
| **Freshwater** | | | | | | |
| As | 62 | 1.80E-04 | 2.37E-04 | 9.91E-05 | 5.80E-07 | 1.13E-03 |
| Cd | 57 | 9.23E-05 | 1.65E-04 | 2.89E-05 | 1.74E-07 | 8.31E-04 |
| Cr | 62 | 1.31E-03 | 1.95E-03 | 5.61E-04 | 1.04E-07 | 9.16E-03 |
| Cu | 56 | 2.76E-03 | 3.29E-03 | 1.36E-03 | 1.36E-06 | 1.94E-02 |
| Hg | 47 | 8.87E-05 | 1.58E-04 | 2.92E-05 | 1.04E-07 | 7.79E-04 |
| Mn | 45 | 4.00E-03 | 6.29E-03 | 1.23E-03 | 3.13E-06 | 2.38E-02 |
| Ni | 47 | 9.25E-04 | 1.68E-03 | 4.53E-04 | 6.26E-07 | 1.03E-02 |
| Pb | 64 | 9.90E-04 | 1.32E-03 | 6.18E-04 | 5.22E-07 | 7.47E-03 |
| Zn | 50 | 2.91E-02 | 4.02E-02 | 1.48E-02 | 1.00E-05 | 1.90E-01 |
| **Euryhaline** | | | | | | |
| As | 9 | 3.61E-04 | 4.11E-04 | 2.07E-04 | 8.39E-06 | 1.15E-03 |
| Cd | 9 | 1.19E-04 | 1.47E-04 | 5.51E-05 | 4.78E-07 | 4.49E-04 |
| Cr | 10 | 1.02E-03 | 1.35E-03 | 6.10E-04 | 1.74E-06 | 4.35E-03 |
| Cu | 9 | 1.34E-03 | 1.49E-03 | 4.66E-04 | 2.12E-05 | 4.48E-03 |
| Hg | 7 | 1.46E-04 | 2.98E-04 | 1.95E-05 | 3.21E-06 | 8.17E-04 |
| Mn | 8 | 1.69E-03 | 3.40E-03 | 2.44E-04 | 1.80E-05 | 9.95E-03 |
| Ni | 9 | 4.81E-04 | 4.78E-04 | 5.01E-04 | 3.39E-06 | 1.53E-03 |
| Pb | 10 | 1.24E-03 | 1.53E-03 | 6.55E-04 | 9.13E-07 | 4.12E-03 |
| Zn | 10 | 1.28E-02 | 2.13E-02 | 5.25E-03 | 2.69E-04 | 7.05E-02 |
| **Seawater** | | | | | | |
| As | 28 | 5.19E-04 | 9.07E-04 | 4.83E-05 | 2.28E-06 | 3.68E-03 |
| Cd | 26 | 9.99E-04 | 3.08E-03 | 6.52E-05 | 0.00E+00 | 1.47E-02 |
| Cr | 29 | 6.62E-04 | 8.61E-04 | 3.55E-04 | 1.04E-05 | 3.77E-03 |
| Cu | 27 | 1.56E-03 | 1.86E-03 | 6.59E-04 | 5.74E-05 | 6.51E-03 |
| Hg | 15 | 1.30E-03 | 2.87E-03 | 1.36E-04 | 8.03E-08 | 8.48E-03 |
| Mn | 22 | 1.19E-03 | 2.52E-03 | 3.92E-04 | 6.78E-06 | 1.20E-02 |
| Ni | 17 | 5.50E-04 | 1.54E-03 | 3.55E-05 | 1.15E-05 | 6.39E-03 |
| Pb | 29 | 1.18E-03 | 2.19E-03 | 7.30E-05 | 2.19E-07 | 9.31E-03 |
| Zn | 26 | 8.32E-03 | 7.37E-03 | 7.50E-03 | 9.37E-04 | 3.27E-02 |
| **Omnivorous** | | | | | | |
| As | 42 | 2.49E-04 | 4.09E-04 | 7.10E-05 | 5.22E-06 | 2.15E-03 |
| Cd | 40 | 1.44E-04 | 3.17E-04 | 2.53E-05 | 1.04E-06 | 1.59E-03 |
| Cr | 43 | 1.34E-03 | 1.94E-03 | 6.23E-04 | 5.78E-06 | 8.25E-03 |
| Cu | 39 | 2.66E-03 | 3.51E-03 | 1.30E-03 | 5.87E-05 | 1.94E-02 |
| Hg | 31 | 1.13E-04 | 1.84E-04 | 4.68E-05 | 2.44E-07 | 7.79E-04 |
| Mn | 31 | 3.09E-03 | 4.91E-03 | 1.14E-03 | 3.34E-05 | 2.19E-02 |
| Ni | 30 | 1.15E-03 | 2.05E-03 | 4.21E-04 | 6.26E-06 | 1.03E-02 |
| Pb | 44 | 1.06E-03 | 1.48E-03 | 6.55E-04 | 1.36E-05 | 7.47E-03 |
| Zn | 37 | 2.54E-02 | 3.16E-02 | 1.33E-02 | 5.73E-04 | 1.43E-01 |
| **Carnivorous** | | | | | | |
| As | 42 | 2.99E-04 | 4.10E-04 | 1.25E-04 | 5.80E-07 | 2.13E-03 |
| Cd | 38 | 6.69E-04 | 2.56E-03 | 7.60E-05 | 1.74E-07 | 1.47E-02 |
| Cr | 43 | 8.62E-04 | 1.53E-03 | 3.95E-04 | 1.04E-05 | 9.16E-03 |
| Cu | 38 | 1.53E-03 | 1.71E-03 | 1.02E-03 | 5.74E-05 | 6.71E-03 |
| Hg | 26 | 8.31E-04 | 2.27E-03 | 5.95E-05 | 2.05E-08 | 8.48E-03 |
| Mn | 30 | 1.68E-03 | 3.28E-03 | 7.27E-04 | 1.81E-05 | 1.77E-02 |
| Ni | 31 | 4.34E-04 | 5.22E-04 | 1.25E-04 | 1.15E-05 | 1.60E-03 |
| Pb | 44 | 1.09E-03 | 1.67E-03 | 6.46E-04 | 2.19E-07 | 9.31E-03 |
| Zn | 35 | 1.14E-02 | 1.44E-02 | 7.88E-03 | 4.10E-04 | 7.49E-02 |
| **Herbivorous** | | | | | | |
| As | 16 | 1.87E-04 | 2.60E-04 | 1.03E-04 | 6.26E-07 | 9.38E-04 |
| Cd | 14 | 4.97E-05 | 5.77E-05 | 3.26E-05 | 5.22E-07 | 2.19E-04 |
| Cr | 16 | 1.04E-03 | 1.60E-03 | 4.62E-04 | 1.04E-07 | 6.27E-03 |
| Cu | 15 | 2.99E-03 | 2.75E-03 | 1.99E-03 | 1.36E-06 | 1.04E-02 |
| Hg | 11 | 7.16E-05 | 1.09E-04 | 1.14E-05 | 1.04E-07 | 2.98E-04 |
| Mn | 13 | 5.35E-03 | 8.28E-03 | 1.17E-03 | 3.13E-06 | 2.38E-02 |
| Ni | 13 | 4.08E-04 | 4.60E-04 | 4.53E-04 | 6.26E-07 | 1.39E-03 |
| Pb | 16 | 7.06E-04 | 1.12E-03 | 4.27E-04 | 5.22E-07 | 4.07E-03 |
| Zn | 14 | 3.38E-02 | 5.78E-02 | 1.29E-02 | 1.00E-05 | 1.90E-01 |

**Supplementary Table S3**: Target hazard quotient (THQ) [unitless] of heavy metal(loid)s due to consumption of various types of fishes in Bangladesh.

|  | **N total** | **Mean** | **Standard Deviation** | **Median** | **Minimum** | **Maximum** |
| --- | --- | --- | --- | --- | --- | --- |
| **Freshwater** | | | | | | |
| As | 62 | 0.599 | 0.789 | 0.330 | 0.0019 | 3.754 |
| Cd | 57 | 0.092 | 0.165 | 0.029 | 0.0002 | 0.831 |
| Cr | 62 | 0.001 | 0.001 | 0.000 | 0.0000001 | 0.006 |
| Cu | 56 | 0.069 | 0.082 | 0.034 | 0.00003 | 0.486 |
| Hg | 47 | 0.296 | 0.526 | 0.097 | 0.0003 | 2.595 |
| Mn | 45 | 0.029 | 0.045 | 0.009 | 0.00002 | 0.170 |
| Ni | 47 | 0.046 | 0.084 | 0.023 | 0.00003 | 0.516 |
| Pb | 64 | 0.283 | 0.377 | 0.177 | 0.0001 | 2.135 |
| Zn | 50 | 0.097 | 0.134 | 0.049 | 0.00003 | 0.634 |
| **Euryhaline** | | | | | | |
| As | 9 | 1.204 | 1.370 | 0.691 | 0.0280 | 3.827 |
| Cd | 9 | 0.119 | 0.147 | 0.055 | 0.0005 | 0.449 |
| Cr | 10 | 0.001 | 0.001 | 0.000 | 0.000001 | 0.003 |
| Cu | 9 | 0.034 | 0.037 | 0.012 | 0.0005 | 0.112 |
| Hg | 7 | 0.487 | 0.994 | 0.065 | 0.0107 | 2.724 |
| Mn | 8 | 0.012 | 0.024 | 0.002 | 0.0001 | 0.071 |
| Ni | 9 | 0.024 | 0.024 | 0.025 | 0.0002 | 0.076 |
| Pb | 10 | 0.354 | 0.436 | 0.187 | 0.0003 | 1.177 |
| Zn | 10 | 0.043 | 0.071 | 0.018 | 0.0009 | 0.235 |
| **Seawater** | | | | | | |
| As | 28 | 1.730 | 3.022 | 0.161 | 0.0076 | 12.261 |
| Cd | 25 | 1.039 | 3.135 | 0.068 | 0.0010 | 14.711 |
| Cr | 29 | 0.000 | 0.001 | 0.000 | 0.00001 | 0.003 |
| Cu | 27 | 0.039 | 0.047 | 0.016 | 0.0014 | 0.163 |
| Hg | 15 | 4.346 | 9.562 | 0.452 | 0.0003 | 28.264 |
| Mn | 22 | 0.009 | 0.018 | 0.003 | 0.00005 | 0.086 |
| Ni | 17 | 0.028 | 0.077 | 0.002 | 0.0006 | 0.320 |
| Pb | 29 | 0.339 | 0.627 | 0.021 | 0.0001 | 2.659 |
| Zn | 26 | 0.028 | 0.025 | 0.025 | 0.0031 | 0.109 |
| **Omnivorous** | | | | | | |
| As | 42 | 0.831 | 1.364 | 0.237 | 0.0174 | 7.182 |
| Cd | 40 | 0.144 | 0.317 | 0.025 | 0.0010 | 1.586 |
| Cr | 43 | 0.001 | 0.001 | 0.000 | 0.0000 | 0.006 |
| Cu | 39 | 0.066 | 0.088 | 0.032 | 0.0015 | 0.486 |
| Hg | 31 | 0.376 | 0.615 | 0.156 | 0.0008 | 2.595 |
| Mn | 31 | 0.022 | 0.035 | 0.008 | 0.0002 | 0.156 |
| Ni | 30 | 0.058 | 0.102 | 0.021 | 0.0003 | 0.516 |
| Pb | 44 | 0.302 | 0.422 | 0.187 | 0.0039 | 2.135 |
| Zn | 37 | 0.085 | 0.105 | 0.044 | 0.0019 | 0.476 |
| **Carnivorous** | | | | | | |
| As | 42 | 0.995 | 1.367 | 0.416 | 0.0019 | 7.095 |
| Cd | 38 | 0.669 | 2.563 | 0.076 | 0.0002 | 14.711 |
| Cr | 43 | 0.001 | 0.001 | 0.000 | 0.0000 | 0.006 |
| Cu | 38 | 0.038 | 0.043 | 0.025 | 0.0014 | 0.168 |
| Hg | 26 | 2.772 | 7.567 | 0.198 | 0.0001 | 28.264 |
| Mn | 30 | 0.012 | 0.023 | 0.005 | 0.0001 | 0.126 |
| Ni | 31 | 0.022 | 0.026 | 0.006 | 0.0006 | 0.080 |
| Pb | 44 | 0.311 | 0.477 | 0.185 | 0.0001 | 2.659 |
| Zn | 35 | 0.038 | 0.048 | 0.026 | 0.0014 | 0.250 |
| **Herbivorous** | | | | | | |
| As | 16 | 0.622 | 0.866 | 0.345 | 0.0021 | 3.128 |
| Cd | 14 | 0.050 | 0.058 | 0.033 | 0.0005 | 0.219 |
| Cr | 16 | 0.001 | 0.001 | 0.000 | 0.0000001 | 0.004 |
| Cu | 15 | 0.075 | 0.069 | 0.050 | 0.00003 | 0.259 |
| Hg | 11 | 0.239 | 0.362 | 0.038 | 0.00035 | 0.992 |
| Mn | 13 | 0.038 | 0.059 | 0.008 | 0.00002 | 0.170 |
| Ni | 13 | 0.020 | 0.023 | 0.023 | 0.00003 | 0.070 |
| Pb | 16 | 0.202 | 0.320 | 0.122 | 0.00015 | 1.162 |
| Zn | 14 | 0.113 | 0.193 | 0.043 | 0.00003 | 0.634 |

**Supplementary Table S4**: Cancer risks (CRs) [unitless] of heavy metal(loid)s due to consumption of various types of fishes in Bangladesh.

|  | **N total** | **Mean** | **Standard Deviation** | **Median** | **Minimum** | **Maximum** |
| --- | --- | --- | --- | --- | --- | --- |
| **Freshwater** | | | | | | |
| As | 62 | 8.98E-01 | 1.18E+00 | 4.96E-01 | 2.90E-03 | 5.63E+00 |
| Cd | 57 | 3.51E-02 | 6.28E-02 | 1.10E-02 | 6.61E-05 | 3.16E-01 |
| Cr | 62 | 4.36E-04 | 6.52E-04 | 1.87E-04 | 3.48E-08 | 3.05E-03 |
| Ni | 47 | 7.87E-02 | 1.43E-01 | 3.85E-02 | 5.32E-05 | 8.78E-01 |
| Pb | 64 | 2.40E-03 | 3.21E-03 | 1.50E-03 | 1.27E-06 | 1.82E-02 |
| Total CR | 64 | 9.62E-01 | 1.18E+00 | 5.74E-01 | 3.38E-03 | 5.67E+00 |
| **Euryhaline** | | | | | | |
| As | 9 | 1.81E+00 | 2.06E+00 | 1.04E+00 | 4.20E-02 | 5.74E+00 |
| Cd | 9 | 4.54E-02 | 5.60E-02 | 2.09E-02 | 1.82E-04 | 1.70E-01 |
| Cr | 10 | 3.40E-04 | 4.50E-04 | 2.03E-04 | 5.80E-07 | 1.45E-03 |
| Ni | 9 | 4.09E-02 | 4.06E-02 | 4.26E-02 | 2.88E-04 | 1.30E-01 |
| Pb | 10 | 3.01E-03 | 3.71E-03 | 1.59E-03 | 2.22E-06 | 1.00E-02 |
| Total CR | 10 | 1.71E+00 | 2.05E+00 | 9.07E-01 | 4.24E-02 | 5.91E+00 |
| **Seawater** | | | | | | |
| As | 28 | 2.59E+00 | 4.53E+00 | 2.41E-01 | 1.14E-02 | 1.84E+01 |
| Cd | 25 | 3.95E-01 | 1.19E+00 | 2.58E-02 | 3.96E-04 | 5.59E+00 |
| Cr | 29 | 2.21E-04 | 2.87E-04 | 1.18E-04 | 3.48E-06 | 1.26E-03 |
| Ni | 17 | 4.68E-02 | 1.31E-01 | 3.02E-03 | 9.76E-04 | 5.43E-01 |
| Pb | 29 | 2.88E-03 | 5.33E-03 | 1.77E-04 | 5.32E-07 | 2.26E-02 |
| Total CR | 29 | 2.88E+00 | 4.60E+00 | 7.17E-01 | 8.87E-04 | 1.91E+01 |
| **Omnivorous** | | | | | | |
| As | 42 | 1.25E+00 | 2.05E+00 | 3.55E-01 | 2.61E-02 | 1.08E+01 |
| Cd | 40 | 5.47E-02 | 1.21E-01 | 9.63E-03 | 3.96E-04 | 6.03E-01 |
| Cr | 43 | 4.45E-04 | 6.46E-04 | 2.08E-04 | 1.93E-06 | 2.75E-03 |
| Ni | 30 | 9.78E-02 | 1.74E-01 | 3.58E-02 | 5.32E-04 | 8.78E-01 |
| Pb | 44 | 2.57E-03 | 3.59E-03 | 1.59E-03 | 3.29E-05 | 1.82E-02 |
| Total CR | 44 | 1.31E+00 | 2.04E+00 | 5.37E-01 | 2.80E-02 | 1.12E+01 |
| **Carnivorous** | | | | | | |
| As | 42 | 1.49E+00 | 2.05E+00 | 6.24E-01 | 2.90E-03 | 1.06E+01 |
| Cd | 38 | 2.54E-01 | 9.74E-01 | 2.89E-02 | 6.61E-05 | 5.59E+00 |
| Cr | 43 | 2.87E-04 | 5.09E-04 | 1.32E-04 | 3.48E-06 | 3.05E-03 |
| Ni | 31 | 3.69E-02 | 4.43E-02 | 1.06E-02 | 9.76E-04 | 1.36E-01 |
| Pb | 44 | 2.64E-03 | 4.05E-03 | 1.57E-03 | 5.32E-07 | 2.26E-02 |
| Total CR | 44 | 1.67E+00 | 2.16E+00 | 7.45E-01 | 8.87E-04 | 1.10E+01 |
| **Herbivorous** | | | | | | |
| As | 16 | 9.33E-01 | 1.30E+00 | 5.17E-01 | 3.13E-03 | 4.69E+00 |
| Cd | 14 | 1.89E-02 | 2.19E-02 | 1.24E-02 | 1.98E-04 | 8.33E-02 |
| Cr | 16 | 3.48E-04 | 5.32E-04 | 1.54E-04 | 3.48E-08 | 2.09E-03 |
| Ni | 13 | 3.47E-02 | 3.91E-02 | 3.85E-02 | 5.32E-05 | 1.18E-01 |
| Pb | 16 | 1.72E-03 | 2.72E-03 | 1.04E-03 | 1.27E-06 | 9.88E-03 |
| Total CR | 16 | 9.79E-01 | 1.33E+00 | 5.72E-01 | 3.38E-03 | 4.78E+00 |

**Supplementary Table S5**: Representation of the categorical data and numeric values using Noncomparative Numerical Notation) followed by data encoding based on fish species.

| **Fish species** | **Assigned value** | **Fish species** | **Assigned value** |
| --- | --- | --- | --- |
| *Gudusia chapra* | 0 | *Gagata youssoufi* | 34 |
| *Setipinna phasa* | 1 | *Mastacembelus pancalus* | 35 |
| *Notropis atherinoides* | 2 | *Pangasius pangasius* | 36 |
| *Notropis atherinoides* | 3 | *Clarias batrachus* | 37 |
| *Rhinomugil corsula* | 4 | *Rita rita* | 38 |
| *Channa punctatus* | 5 | *Puntius chola* | 39 |
| *Mystus vittatus* | 6 | *Puntius sophore* | 40 |
| *Awaous guamensis* | 7 | *Barilius barila* | 41 |
| *Awaous guamensis* | 8 | *Salmostoma acinaces* | 42 |
| *Puntius ticto* | 9 | *Labeo bata* | 43 |
| *Labeo calbasu* | 10 | *Sperata aor* | 44 |
| *Clupisoma garua* | 11 | *Ailiichthys punctata* | 45 |
| *Labeo rohita* | 12 | *Cirrhinus cirrhosus* | 46 |
| *Anabus testudineus* | 13 | *Eutropiichthys vacha* | 47 |
| *Channa striata* | 14 | *Mystus bleekeri* | 48 |
| *Notopterus notopterus* | 15 | *Rohtee cotio* | 49 |
| *Silonia silondia* | 16 | *Clarias gariepinus* | 50 |
| *Planiliza subviridis* | 17 | *Hypophthalmichthys molitrix* | 51 |
| *Ailia coila* | 18 | *Cyprinus carpio* | 52 |
| *Hyporhamphus limbatus* | 19 | *Puntius sarana* | 53 |
| *Glossogobius Giuris* | 20 | *Mystus tengara* | 54 |
| *Cirrhinus reba* | 21 | *Ompok pabda* | 55 |
| *Mastacembelus armatus* | 22 | *Xenentodon cancila* | 56 |
| *Corica soborna* | 23 | *Salmophasia phulo* | 57 |
| *Amblypharyngodon mola* | 24 | *Parambassis ranga* | 58 |
| *Heteropneustes fossilis* | 25 | *Nandus nandus* | 59 |
| *Pseudeutropius atherinoides* | 26 | *Acanthocobitis botia* | 60 |
| *Oreochromis mossambicus* | 27 | *Chanda nama* | 61 |
| *Oreochromis niloticus* | 28 | *Bagarius bagarius* | 62 |
| *Catla catla* | 29 | *Leporinus fasciatus* | 63 |
| *Wallago attu* | 30 | *Tenualosa ilisha* | 64 |
| *Pangasianodon hypophthalmus* | 31 | *Tenualosa toli* | 65 |
| *Batasio batasio* | 32 | *Ilisha megaloptera* | 66 |
| *Trichogaster fasciata* | 33 | *Polynemus paradiseus* | 67 |
| ***Table S5*** *(Cont.)* |  |  |  |
| **Fish species** | **Assigned value** | **Fish species** | **Assigned value** |
| *Pampus chinensis* | 68 | *Lutjanus johnii* | 86 |
| *Pampus argenteus* | 69 | *Eubleekeria splendens* | 87 |
| *Liza parsia* | 70 | *Cynoglossus cynoglossus* | 88 |
| *Lates calcarifer* | 71 | *Setipinna taty* | 89 |
| *Mystus gulio* | 72 | *Sardinella longiceps* | 90 |
| *Otolithoides pama* | 73 | *Panna microdon* | 91 |
| *Harpadon nehereus* | 74 | *Coilia dussumieri* | 92 |
| *Sillaginopsis panijus* | 75 | *Trichiurus lepturus* | 93 |
| *Cynoglossus arel* | 76 | *Pomatoschistus microps* | 94 |
| *Mugil cephalus* | 77 | *Rastrelliger kanagurta* | 95 |
| *Polynemus indicus* | 78 | *Halichoeres nigrescens* | 96 |
| *Scomberomorus guttatus* | 79 | *Pomacentrus cuneatus* | 97 |
| *Arius arius* | 80 | *Pomacanthus annularis* | 98 |
| *Johnius coitor* | 81 | *Sargocentron rubrum* | 99 |
| *Otolithes ruber* | 82 | *Lethrinus atkinsoni* | 100 |
| *Scomber australasicus* | 83 | *Terapon jarbua* | 101 |
| *Etrumeus acuminatus* | 84 | *Pseudapocryptes elongatus* | 102 |
| *Arius maculatus* | 85 |  |  |

**Supplementary Table S6**: Representation of the Categorical data and numeric values using Noncomparative Numerical Notation) followed by data encoding based on fish habitat and categories.

| **Categorical Data** | **Encoded Data** | **Categorical Data** | **Encoded Data** |
| --- | --- | --- | --- |
| Freshwater | 0 | Omnivorous | 3 |
| Euryhaline | 1 | Carnivorous | 4 |
| Saltwater | 2 | Herbivorous | 5 |

**Supplementary Table S7:** Individual fish safe intake limit by Habitat type

| Habitat | Species | Safe Consumption | Average | Habitat | | Species | Safe Consumption | Average |
| --- | --- | --- | --- | --- | --- | --- | --- | --- |
| Freshwater | *Hyporhamphus limbatus* | 16.68 | **180.59** | Saltwater | | *Arius arius* | 5.11 | **156.51** |
| Freshwater | *Oreochromis mossambicus* | 20.93 |  | Saltwater | | *Sillaginopsis panijus* | 6.97 |  |
| Freshwater | *Notropis atherinoides* | 24.72 |  | Saltwater | | *Otolithoides pama* | 9.69 |  |
| Freshwater | *Cirrhinus reba* | 27.12 |  | Saltwater | | *Cynoglossus arel* | 13.82 |  |
| Freshwater | *Setipinna phasa* | 28.23 |  | Saltwater | | *Mugil cephalus* | 17.53 |  |
| Freshwater | *Gudusia chapra* | 27.2 |  | Saltwater | | *Harpadon nehereus* | 17.76 |  |
| Freshwater | *Rita rita* | 36.05 |  | Saltwater | | *Scomberomorus guttatus* | 24.9 |  |
| Freshwater | *Mystus vittatus* | 45.64 |  | Saltwater | | *Polynemus indicus* | 32.16 |  |
| Freshwater | *Pangasius pangasius* | 51.03 |  | Saltwater | | *Otolithes ruber* | 360 |  |
| Freshwater | *Batasio batasio* | 51.05 |  | Saltwater | | *Scomber australasicus* | 145.16 |  |
| Freshwater | *Silonia silondia* | 59.94 |  | Saltwater | | *Etrumeus acuminatus* | 121.62 |  |
| Freshwater | *Rhinomugil corsula* | 103.71 |  | Saltwater | | *Arius maculatus* | 131.39 |  |
| Freshwater | *Awaous guamensis* | 188.03 |  | Saltwater | | *Lutjanus johnii* | 63.33 |  |
| Freshwater | *Planiliza subviridis* | 87.46 |  | Saltwater | | *Eubleekeria splendens* | 63.65 |  |
| Freshwater | *Glossogobius Giuris* | 327.15 |  | Saltwater | | *Cynoglossus cynoglossus* | 666.67 |  |
| Freshwater | *Pangasianodon hypophthalmus* | 259.59 |  | Saltwater | | *Setipinna taty* | 1210.55 |  |
| Freshwater | *Trichogaster fasciata* | 102.57 |  | Saltwater | | *Panna microdon* | 2.21 |  |
| Freshwater | *Gagata youssoufi* | 98.71 |  | Saltwater | | *Halichoeres nigrescens* | 4.26 |  |
| Freshwater | *Mystus tengara* | 186.34 |  | Saltwater | | *Sargocentron rubrum* | 9.68 |  |
| Freshwater | *Chanda nama* | 372.74 |  | Saltwater | | *Rastrelliger kanagurta* | 39.47 |  |
| Freshwater | *Bagarius bagarius* | 753.45 |  | Saltwater | | *Coilia dussumieri* | 2.3 |  |
| Freshwater | *Puntius ticto* | 75.36 |  | Saltwater | | *Trichiurus lepturus* | 18.64 |  |
| Freshwater | *Notropis atherinoides* | 121.25 |  | Saltwater | | *Pomatoschistus microps* | 103.27 |  |
| Freshwater | *Wallago attu* | 135.41 |  | Saltwater | | *Johnius coitor* | 112.5 |  |
| Freshwater | *Amblypharyngodon mola* | 24.12 |  | Saltwater | | *Pomacentrus cuneatus* | 138.46 |  |
| Freshwater | *Catla catla* | 63.12 |  | Saltwater | | *Lethrinus atkinsoni* | 225 |  |
| Freshwater | *Puntius sophore* | 68.38 |  | Saltwater | | *Terapon jarbua* | 257.14 |  |
| Freshwater | *Ailiichthys punctata* | 75 |  | Saltwater | | *Sardinella longiceps* | 285.71 |  |
| Freshwater | *Clarias batrachus* | 87.8 |  | Saltwater | | *Pomacanthus annularis* | 450 |  |
| Freshwater | *Eutropiichthys vacha* | 112.5 |  | Euryhaline | | *Polynemus paradiseus* | 16.36 | **182,17** |
| Freshwater | *Cirrhinus cirrhosus* | 128.57 |  | Euryhaline | | *Liza parsia* | 20.01 |  |
| Freshwater | *Rohtee cotio* | 128.57 |  | Euryhaline | | *Lates calcarifer* | 49.13 |  |
| Freshwater | *Clarias gariepinus* | 70.04 |  | Euryhaline | | *Ilisha megaloptera* | 55.49 |  |
| Freshwater | *Parambassis ranga* | 178.22 |  | Euryhaline | | *Tenualosa toli* | 63.33 |  |
| Freshwater | *Acanthocobitis botia* | 180 |  | Euryhaline | | *Mystus gulio* | 819.25 |  |
| Freshwater | *Mystus bleekeri* | 220.59 |  | Euryhaline | | *Pampus argenteus* | 178.35 |  |
| Freshwater | *Salmostoma acinaces* | 315.78 |  | Euryhaline | | *Glossogobius Giuris* | 137.4 |  |
| Freshwater | *Anabus testudineus* | 303.23 |  | Euryhaline | | *Tenualosa ilisha* | 391.85 |  |
| Freshwater | *Sperata aor* | 409.08 |  | Euryhaline | | *Pampus chinensis* | 90.53 |  |
| Freshwater | *Puntius sarana* | 450 |  |  | |  |  |  |
| Freshwater | *Salmophasia phulo* | 356.01 |  |  |  |  |  |  |
| Freshwater | *Ompok pabda* | 473.46 |  |  |  |  |  |  |
| Freshwater | *Xenentodon cancila* | 311.03 |  |  |  |  |  |  |
| Freshwater | *Labeo rohita* | 173.17 |  |  |  |  |  |  |
| Freshwater | *Nandus nandus* | 642.86 |  |  |  |  |  |  |
| Freshwater | *Awaous guamensis* | 419.58 |  |  |  |  |  |  |
| Freshwater | *Channa punctatus* | 178.7 |  |  |  |  |  |  |
| Freshwater | *Pseudeutropius atherinoides* | 190.94 |  |  |  |  |  |  |
| Freshwater | *Puntius chola* | 140.75 |  |  |  |  |  |  |
| Freshwater | *Clupisoma garua* | 97.91 |  |  |  |  |  |  |
| Freshwater | *Corica soborna* | 83.15 |  |  |  |  |  |  |
| Freshwater | *Notopterus notopterus* | 132.8 |  |  |  |  |  |  |
| Freshwater | *Mastacembelus armatus* | 124.31 |  |  |  |  |  |  |
| Freshwater | *Hypophthalmichthys molitrix* | 150.63 |  |  |  |  |  |  |
| Freshwater | *Leporinus fasciatus* | 450 |  |  |  |  |  |  |
| Freshwater | *Barilius barila* | 216.86 |  |  |  |  |  |  |
| Freshwater | *Channa striata* | 156.78 |  |  |  |  |  |  |
| Freshwater | *Ailia coila* | 160.12 |  |  |  |  |  |  |
|  |  |  |  |  |  |  |  |  |

**Supplementary Table S8:** Individual fish safe intake limit by feeding behaviour.

| **Food Type** | **Species** | **Safe Consumption** | **Average** | **Food Type** | **Species** | **Safe Consumption** | **Average** |
| --- | --- | --- | --- | --- | --- | --- | --- |
| Omnivorous | *Sillaginopsis panijus* | 8.72 | **168.63** | Carnivorous | *Sillaginopsis panijus* | 8.72 | **153.05** |
| Omnivorous | *Mugil cephalus* | 17.53 |  | Carnivorous | *Mugil cephalus* | 17.53 |  |
| Omnivorous | *Tenualosa toli* | 19.73 |  | Carnivorous | *Tenualosa toli* | 19.73 |  |
| Omnivorous | *Oreochromis mossambicus* | 20.93 |  | Carnivorous | *Oreochromis mossambicus* | 20.93 |  |
| Omnivorous | *Setipinna phasa* | 28.23 |  | Carnivorous | *Ilisha megaloptera* | 28.42 |  |
| Omnivorous | *Ilisha megaloptera* | 28.42 |  | Carnivorous | *Gudusia chapra* | 27.2 |  |
| Omnivorous | *Gudusia chapra* | 27.2 |  | Carnivorous | *Pseudapocryptes elongatus* | 76.37 |  |
| Omnivorous | *Mystus vittatus* | 45.64 |  | Carnivorous | *Glossogobius Giuris* | 137.4 |  |
| Omnivorous | *Pangasius pangasius* | 51.03 |  | Carnivorous | *Heteropneustes fossilis* | 187.24 |  |
| Omnivorous | *Rhinomugil corsula* | 103.71 |  | Carnivorous | *Notropis atherinoides* | 121.25 |  |
| Omnivorous | *Awaous guamensis* | 188.03 |  | Carnivorous | *Tenualosa ilisha* | 378.15 |  |
| Omnivorous | *Glossogobius Giuris* | 327.15 |  | Carnivorous | *Puntius ticto* | 75.36 |  |
| Omnivorous | *Pangasianodon hypophthalmus* | 259.59 |  | Carnivorous | *Oreochromis niloticus* | 141.8 |  |
| Omnivorous | *Trichogaster fasciata* | 102.57 |  | Carnivorous | *Pangasianodon hypophthalmus* | 259.59 |  |
| Omnivorous | *Mystus gulio* | 63.33 |  | Carnivorous | *Mystus gulio* | 70 |  |
| Omnivorous | *Eubleekeria splendens* | 529.41 |  | Carnivorous | *Rhinomugil corsula* | 103.71 |  |
| Omnivorous | *Setipinna taty* | 63.8 |  | Carnivorous | *Awaous guamensis* | 188.03 |  |
| Omnivorous | *Pseudapocryptes elongatus* | 76.37 |  | Carnivorous | *Glossogobius Giuris* | 327.15 |  |
| Omnivorous | *Rastrelliger kanagurta* | 39.47 |  | Carnivorous | *Trichogaster fasciata* | 102.57 |  |
| Omnivorous | *Puntius ticto* | 75.36 |  | Carnivorous | *Eubleekeria splendens* | 63.33 |  |
| Omnivorous | *Notropis atherinoides* | 121.25 |  | Carnivorous | *Setipinna taty* | 63.33 |  |
| Omnivorous | *Amblypharyngodon mola* | 24.12 |  | Carnivorous | *Pomacentrus cuneatus* | 78.98 |  |
| Omnivorous | *Puntius sophore* | 68.38 |  | Carnivorous | *Pomacanthus annularis* | 175.24 |  |
| Omnivorous | *Clarias batrachus* | 87.8 |  | Carnivorous | *Setipinna phasa* | 28.23 |  |
| Omnivorous | *Rohtee cotio* | 128.57 |  | Carnivorous | *Amblypharyngodon mola* | 24.12 |  |
| Omnivorous | *Clarias gariepinus* | 70.04 |  | Carnivorous | *Puntius sophore* | 68.38 |  |
| Omnivorous | *Pomacentrus cuneatus* | 138.46 |  | Carnivorous | *Clarias batrachus* | 87.8 |  |
| Omnivorous | *Parambassis ranga* | 178.22 |  | Carnivorous | *Rohtee cotio* | 128.57 |  |
| Omnivorous | *Acanthocobitis botia* | 180 |  | Carnivorous | *Clarias gariepinus* | 70.04 |  |
| Omnivorous | *Mystus bleekeri* | 220.59 |  | Carnivorous | *Parambassis ranga* | 178.22 |  |
| Omnivorous | *Terapon jarbua* | 257.14 |  | Carnivorous | *Acanthocobitis botia* | 180 |  |
| Omnivorous | *Salmostoma acinaces* | 315.78 |  | Carnivorous | *Mystus bleekeri* | 220.59 |  |
| Omnivorous | *Anabus testudineus* | 63.33 |  | Carnivorous | *Rastrelliger kanagurta* | 39.47 |  |
| Omnivorous | *Puntius sarana* | 450 |  | Carnivorous | *Terapon jarbua* | 257.14 |  |
| Omnivorous | *Salmophasia phulo* | 356.01 |  | Carnivorous | *Salmostoma acinaces* | 315.78 |  |
| Omnivorous | *Awaous guamensis* | 718.79 |  | Carnivorous | *Anabus testudineus* | 303.23 |  |
| Omnivorous | *Glossogobius Giuris* | 137.4 |  | Carnivorous | *Puntius sarana* | 450 |  |
| Omnivorous | *Puntius chola* | 140.75 |  | Carnivorous | *Salmophasia phulo* | 356.01 |  |
| Omnivorous | *Leporinus fasciatus* | 450 |  | Carnivorous | *Awaous guamensis* | 587.54 |  |
| Omnivorous | *Pomacanthus annularis* | 450 |  | Carnivorous | *Mystus vittatus* | 45.64 |  |
| Omnivorous | *Tenualosa ilisha* | 378.15 |  | Carnivorous | *Puntius chola* | 140.75 |  |
| Omnivorous | *Oreochromis niloticus* | 141.8 |  | Carnivorous | *Leporinus fasciatus* | 450 |  |
| Omnivorous | *Cyprinus carpio* | 79.75 |  | Carnivorous | *Pangasius pangasius* | 51.03 |  |
| Omnivorous | *Heteropneustes fossilis* | 187.24 |  | Carnivorous | *Cyprinus carpio* | 79.75 |  |
| Herbivorous | *Liza parsia* | 20.01 | **175.09** |  |  |  |  |
| Herbivorous | *Cirrhinus reba* | 27.12 |  |  |  |  |  |
| Herbivorous | *Planiliza subviridis* | 87.46 |  |  |  |  |  |
| Herbivorous | *Gagata youssoufi* | 98.71 |  |  |  |  |  |
| Herbivorous | *Etrumeus acuminatus* | 121.62 |  |  |  |  |  |
| Herbivorous | *Catla catla* | 63.12 |  |  |  |  |  |
| Herbivorous | *Ailiichthys punctata* | 75 |  |  |  |  |  |
| Herbivorous | *Cirrhinus cirrhosus* | 128.57 |  |  |  |  |  |
| Herbivorous | *Labeo rohita* | 173.17 |  |  |  |  |  |
| Herbivorous | *Pseudeutropius atherinoides* | 190.94 |  |  |  |  |  |
| Herbivorous | *Corica soborna* | 83.15 |  |  |  |  |  |
| Herbivorous | *Hypophthalmichthys molitrix* | 150.63 |  |  |  |  |  |
| Herbivorous | *Sardinella longiceps* | 285.71 |  |  |  |  |  |
| Herbivorous | *Mastacembelus pancalus* | 97.74 |  |  |  |  |  |
| Herbivorous | *Labeo bata* | 1136.53 |  |  |  |  |  |
| Herbivorous | *Labeo calbasu* | 62 |  |  |  |  |  |

**
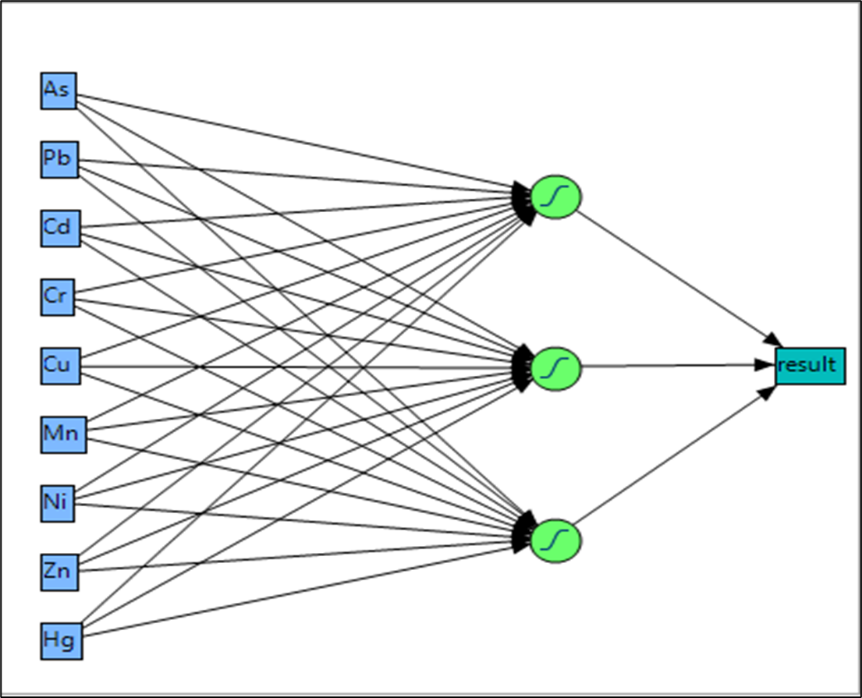
**

**Supplementary Fig. S1.** Flow-diagram of Artificial Neural Network (ANN) processes and data clustering using the driven data of fish types on habitat and feeding behaviour along with the consideration of THQ values, defining the internal and hidden nature of the data.

**
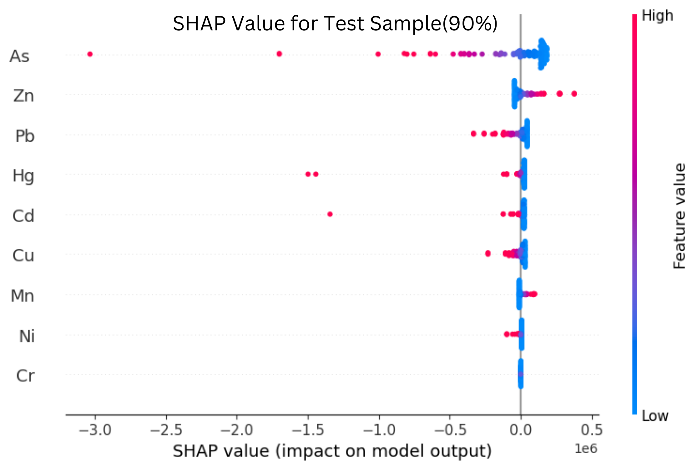

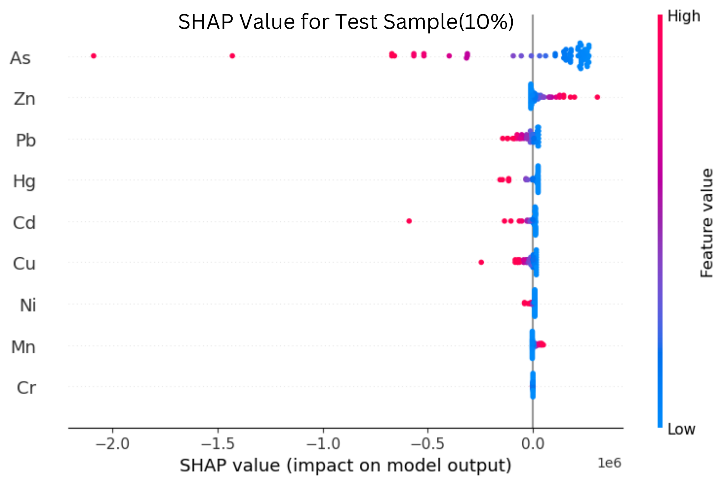
**

**Supplementary Fig. S2:** The SHAP values and influence over the output **(a)** Sample size = 90% (left); **(b)** Sample size=10% (right). SHAP values exert a positive influence on predictions when they are positive values and a negative influence when they are negative values. The magnitude of these values serves as an indicator of the strength of their impact.


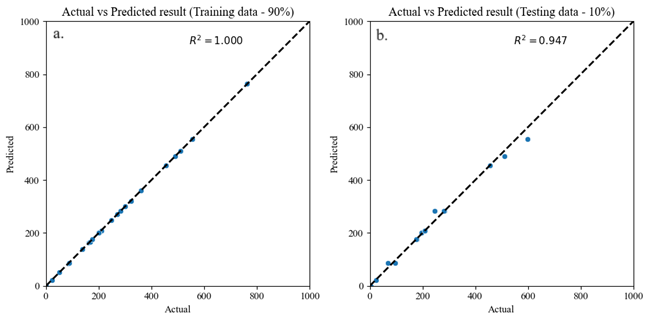


**Supplementary Fig. S3:** The magnitude of the model's actions or performance while the SHAP values and influence over the output from training and testing data; Table shows the performance R^2^ = 94.7% for testing data size, and Fig. (a) shows actual vs predicted training data (90% data size) values, and Fig. (b) actual vs predicted testing data (10% data size) values.

**Articles used for data compilation on heavy metal(oid)s in fish in Bangladesh.**

1. Ahmad, M.K., Islam, S., Rahman, S., Haque, M. and Islam, M.M., 2010. Heavy metals in water, sediment and some fishes of Buriganga River, Bangladesh. International Journal of Environmental Research, 4(2):321-332.
2. Ahmed, M.K., Baki, M.A., Islam, M.S., Kundu, G.K., Habibullah-Al-Mamun, M., Sarkar, S.K. and Hossain, M.M., 2015. Human health risk assessment of heavy metals in tropical fish and shellfish collected from the river Buriganga, Bangladesh. Environmental Science and Pollution Research, 22, pp.15880-15890.
3. Ahmed, M.K., Shaheen, N., Islam, M.S., Habibullah-al-Mamun, M., Islam, S., Mohiduzzaman, M. and Bhattacharjee, L., 2015. Dietary intake of trace elements from highly consumed cultured fish (Labeo rohita, Pangasius pangasius and Oreochromis mossambicus) and human health risk implications in Bangladesh. Chemosphere, 128, pp.284-292.
4. Ahmed, A.S.S., Hossain, M.B., Semme, S.A., Babu, S.M.O.F., Hossain, K. and Moniruzzaman, M., 2020. Accumulation of trace elements in selected fish and shellfish species from the largest natural carp fish breeding basin in Asia: a probabilistic human health risk implication. Environmental Science and Pollution Research, 27, pp.37852-37865.
5. Ahmed, A.S., Rahman, M., Sultana, S., Babu, S.O.F. and Sarker, M.S.I., 2019. Bioaccumulation and heavy metal concentration in tissues of some commercial fishes from the Meghna River Estuary in Bangladesh and human health implications. Marine pollution bulletin, 145, pp.436-447.
6. Ahmed, A.S., Sultana, S., Habib, A., Ullah, H., Musa, N., Hossain, M.B., Rahman, M.M. and Sarker, M.S.I., 2019. Bioaccumulation of heavy metals in some commercially important fishes from a tropical river estuary suggests higher potential health risk in children than adults. Plos one, 14(10), p.e0219336.
7. Ali, M.M., Ali, M.L., Proshad, R., Islam, S., Rahman, Z., Tusher, T.R., Kormoker, T. and Al, M.A., 2020. Heavy metal concentrations in commercially valuable fishes with health hazard inference from Karnaphuli river, Bangladesh. Human and ecological risk assessment: an international journal, 26(10), pp.2646-2662.
8. Ali, M.M., Ali, M.L., Proshad, R., Islam, S., Rahman, Z. and Kormoker, T., 2020. Assessment of trace elements in the demersal fishes of a coastal river in Bangladesh: a public health concern. Thalassas: An International Journal of Marine Sciences, 36, pp.641-655.
9. Baki, M.A., Hossain, M.M., Akter, J., Quraishi, S.B., Shojib, M.F.H., Ullah, A.A. and Khan, M.F., 2018. Concentration of heavy metals in seafood (fishes, shrimp, lobster and crabs) and human health assessment in Saint Martin Island, Bangladesh. Ecotoxicology and environmental safety, 159, pp.153-163.
10. Baki, M.A., Shojib, M.F.H., Sehrin, S., Chakraborty, S., Choudhury, T.R., Bristy, M.S., Ahmed, M.K., Yusoff, S.B. and Khan, M.F., 2020. Health risk assessment of heavy metal accumulation in the Buriganga and Turag River systems for Puntius ticto, Heteropneustes fossilis, and Channa punctatus. Environmental geochemistry and health, 42, pp.531-543.
11. Begum, A., Mustafa, A.I., Amin, M.N., Chowdhury, T.R., Quraishi, S.B. and Banu, N., 2013. Levels of heavy metals in tissues of shingi fish (Heteropneustes fossilis) from Buriganga River, Bangladesh. Environmental Monitoring and Assessment, 185, pp.5461-5469.
12. Bristy, M.S., Sarker, K.K., Baki, M.A., Quraishi, S.B., Hossain, M.M., Islam, A. and Khan, M.F., 2021. Health risk estimation of metals bioaccumulated in commercial fish from coastal areas and rivers in Bangladesh. Environmental toxicology and pharmacology, 86, p.103666.
13. Ghosh, P., Ahmed, Z., Alam, R., Begum, B.A., Akter, S. and Jolly, Y.N., 2021. Bioaccumulation of metals in selected cultured fish species and human health risk assessment: a study in Mymensingh Sadar Upazila, Bangladesh. Stochastic Environmental Research and Risk Assessment, 35(11), pp.2287-2301.
14. Hasan, M.K., Shahriar, A., Hossain, N., Shovon, I.K., Hossain, A., Jolly, Y.N. and Begum, B.A., 2021. Trace metals contamination in riverine captured fish and prawn of Bangladesh and associated health risk. Exposure and Health, 13, pp.237-251.
15. Hossain, M.B., Ahmed, A.S.S. and Sarker, M.S.I., 2018. Human health risks of Hg, As, Mn, and Cr through consumption of fish, Ticto barb (Puntius ticto) from a tropical river, Bangladesh. Environmental Science and Pollution Research, 25(31), pp.31727-31736.
16. Hossain, M.N., Rahaman, A., Hasan, M.J., Uddin, M.M., Khatun, N. and Shamsuddin, S.M., 2021. Comparative seasonal assessment of pollution and health risks associated with heavy metals in water, sediment and Fish of Buriganga and Turag River in Dhaka City, Bangladesh. SN Applied Sciences, 3, pp.1-16.
17. Hoque, M.S., Tamanna, F., Hasan, M.M., Al Banna, M.H., Mondal, P., Prodhan, M.D.H., Rahman, M.Z. and van Brakel, M.L., 2022. Probabilistic public health risks associated with pesticides and heavy metal exposure through consumption of common dried fish in coastal regions of Bangladesh. Environmental Science and Pollution Research, pp.1-16.
18. Islam, M.S., Ahmed, M.K. and Habibullah-Al-Mamun, M., 2015. Determination of heavy metals in fish and vegetables in Bangladesh and health implications. Human and Ecological Risk Assessment: An International Journal, 21(4), pp.986-1006.
19. Islam, M.S., Ahmed, M.K., Habibullah-Al-Mamun, M., Islam, K.N., Ibrahim, M. and Masunaga, S., 2014. Arsenic and lead in foods: a potential threat to human health in Bangladesh. Food Additives & Contaminants: Part A, 31(12), pp.1982-1992.
20. Islam, M.S., Ahmed, M.K. and Habibullah-Al-Mamun, M., 2017. Heavy metals in sediment and their accumulation in commonly consumed fish species in Bangladesh. Archives of environmental & occupational health, 72(1), pp.26-38.
21. Islam, M.S., Ahmed, M.K., Habibullah-Al-Mamun, M. and Masunaga, S., 2015. Assessment of trace metals in fish species of urban rivers in Bangladesh and health implications. Environmental toxicology and pharmacology, 39(1), pp.347-357.
22. Islam, M.S., Ahmed, M.K., Habibullah-Al-Mamun, M. and Raknuzzaman, M., 2015. The concentration, source and potential human health risk of heavy metals in the commonly consumed foods in Bangladesh. Ecotoxicology and environmental safety, 122, pp.462-469.
23. Islam, M.S., Ahmed, M.K., Raknuzzaman, M., Habibullah-Al-Mamun, M. and Masunaga, S., 2015. Metal speciation in sediment and their bioaccumulation in fish species of three urban rivers in Bangladesh. Archives of environmental contamination and toxicology, 68, pp.92-106.
24. Islam, M.S., Rahman, M.M., Kabir, M.H., Hoq, M.E., Meghla, N.T., Suravi, S., Al Mamun, S. and Sarker, M.E., 2021. Seasonal Dynamics of Heavy Metal Concentrations in Water, Fish and Sediments from Haor Region of Bangladesh. Pollution, 7(4), pp.843-857.
25. Islam, S., Bhowmik, S., Hossain, M.K., Nordin, N., Rahman, M., Ahmmed, M.K., Parvin, A. and Hossain, M.A., 2021. Tilapia from most of the sources in Bangladesh are safe for human consumption: A Hazard Index (HI) based study on heavy metals. Journal of Aquatic Food Product Technology, 30(8), pp.1017-1027.
26. Islam, M.M., Avha, N.J., Ahmed, S., Akbor, M.A., Islam, M.S., Mostafiz, F. and Habibullah-Al-Mamun, M., 2021. Trace metals and organochlorine pesticide residues in imported fishes in Bangladesh and human health risk implications. Environmental Science and Pollution Research, pp.1-14.
27. Islam, G.R., Khan, F.E., Hoque, M.M. and Jolly, Y.N., 2014. Consumption of unsafe food in the adjacent area of Hazaribag tannery campus and Buriganga River embankments of Bangladesh: heavy metal contamination. Environmental monitoring and assessment, 186, pp.7233-7244.
28. Jolly, Y.N., Rakib, M.R.J., Islam, M.S., Akter, S., Idris, A.M. and Phoungthong, K., 2022. Potential toxic elements in sediment and fishes of an important fish breeding river in Bangladesh: a preliminary study for ecological and health risks assessment. Toxin reviews, 41(3), pp.945-958.
29. Kawser Ahmed, M., Baki, M.A., Kundu, G.K., Islam, S., Islam, M. and Hossain, M., 2016. Human health risks from heavy metals in fish of Buriganga river, Bangladesh. SpringerPlus, 5(1), pp.1-12.
30. Khan, F.E., Jolly, Y.N., Islam, G.R., Akhter, S. and Kabir, J., 2014. Contamination status and health risk assessment of trace elements in foodstuffs collected from the Buriganga River embankments, Dhaka, Bangladesh. International Journal of Food Contamination, 1, pp.1-8.
31. Khatun, N., Nayeem, J., deb, N., Hossain, S. and Kibria, M.M., 2021. Heavy metals contamination: possible health risk assessment in highly consumed fish species and water of Karnafuli River Estuary, Bangladesh. Toxicology and Environmental Health Sciences, 13(4), pp.375-388.
32. Lipy, E.P., Hakim, M., Mohanta, L.C., Islam, D., Lyzu, C., Roy, D.C., Jahan, I., Akhter, S., Raknuzzaman, M. and Abu Sayed, M., 2021. Assessment of heavy metal concentration in water, sediment and common fish species of Dhaleshwari River in Bangladesh and their health implications. Biological Trace Element Research, 199, pp.4295-4307.
33. Maruf, M.A.H., Punom, N.J., Saha, B., Moniruzzaman, M., Suchi, P.D., Eshik, M.M.E. and Rahman, M.S., 2021. Assessment of human health risks associated with heavy metals accumulation in the freshwater fish Pangasianodon hypophthalmus in Bangladesh. Exposure and Health, 13(3), pp.337-359.
34. Marcussen, H., Alam, M.A., Rahman, M.M., Ali, M.L., Mahmud, S. and Jørgensen, N.O., 2014. Species-specific content of As, Pb, and other elements in pangas (Pangasianodon hypophthalmus) and tilapia (Oreochromis niloticus) from aquaculture ponds in southern Bangladesh. Aquaculture, 426, pp.85-87.
35. Musarrat, M., Ullah, A.A., Moushumi, N.S., Akon, S., Nahar, Q., Sultana, S.S.S. and Quraishi, S.B., 2021. Assessment of heavy metal (oid) s in selected small indigenous species of industrial area origin freshwater fish and potential human health risk implications in Bangladesh. LWT, 150, p.112041.
36. Nargis, A., Jhumur, A.K., Haque, M.E., Islam, M.N., Habib, A. and Cai, M., 2019. Human health risk assessment of toxic elements in fish species collected from the river Buriganga, Bangladesh. Human and Ecological Risk Assessment: An International Journal.
37. Rahman, M.S., Molla, A.H., Saha, N. and Rahman, A., 2012. Study on heavy metals levels and its risk assessment in some edible fishes from Bangshi River, Savar, Dhaka, Bangladesh. Food chemistry, 134(4), pp.1847-1854.
38. Rakib, M.R.J., Jolly, Y.N., Enyoh, C.E., Khandaker, M.U., Hossain, M.B., Akther, S., Alsubaie, A., Almalki, A.S. and Bradley, D.A., 2021. Levels and health risk assessment of heavy metals in dried fish consumed in Bangladesh. Scientific reports, 11(1), p.14642.
39. Saha, N. and Zaman, M.R., 2013. Evaluation of possible health risks of heavy metals by consumption of foodstuffs available in the central market of Rajshahi City, Bangladesh. Environmental monitoring and assessment, 185, pp.3867-3878.
40. Saha, N., Mollah, M.Z.I., Alam, M.F. and Rahman, M.S., 2016. Seasonal investigation of heavy metals in marine fishes captured from the Bay of Bengal and the implications for human health risk assessment. Food control, 70, pp.110-118.
41. Sarker, M.J., Islam, M.A., Rahman, F. and Anisuzzaman, M., 2021. Heavy Metals in the Fish Tenualosa ilisha Hamilton, 1822 in the Padma–Meghna River Confluence: Potential Risks to Public Health. Toxics, 9(12), p.341.
42. Sarker, M.J., Polash, A.U., Islam, M.A., Rima, N.N. and Farhana, T., 2020. Heavy metals concentration in native edible fish at upper Meghna River and its associated tributaries in Bangladesh: a prospective human health concern. SN Applied Sciences, 2, pp.1-13.
43. Shorna, S., Shawkat, S., Hossain, A., Quraishi, S.B., Ullah, A.A., Hosen, M.M., Hossain, M.K., Saha, B., Paul, B. and Habibullah-Al-Mamun, M., 2021. Accumulation of trace metals in indigenous fish species from the Old Brahmaputra River in Bangladesh and human health risk implications. Biological Trace Element Research, 199, pp.3478-3488.
44. Ullah, A.A., Maksud, M.A., Khan, S.R., Lutfa, L.N. and Quraishi, S.B., 2017. Dietary intake of heavy metals from eight highly consumed species of cultured fish and possible human health risk implications in Bangladesh. Toxicology Reports, 4, pp.574-579.
45. Wahiduzzaman, M., Islam, M.M., Sikder, A.H.F. and Parveen, Z., 2021. Bioaccumulation and heavy metal contamination in fish species of the Dhaleswari River of Bangladesh and related human health implications. Biological Trace Element Research, pp.1-13.
46. Zakir, H.M., Quadir, Q.F. and Mollah, M.Z.I., 2021. Human health risk assessment of heavy metals through the consumption of common foodstuffs collected from two divisional cities of Bangladesh. Exposure and Health, 13, pp.253-268.
