# Supplementary material for: Exploring Potential Human Health Risks Linked to Heavy Metal(Loid)s in Dietary Fishes: Utilizing Data-Driven and Computational Modelling Approaches
Source: Biol Trace Elem Res. 2024 Sep 11;203(5):2830–47. doi: 10.1007/s12011-024-04363-6 (PMC12125157; doi:10.1007/s12011-024-04363-6)

***Supplementary information for***

**Exploring potential human health risks linked to heavy metal(loid)s in dietary fishes: Utilizing data-driven and computational modelling approaches**

Pritom Bhowmik Akash^1^, Sazal Kumar^2^, Md. Saikoth Jahan^3^, Muhammad Shafiqur Rahman^4^, Md. Assraf Seddiky^5^, Anti Sorker^6^, and Rafiquel Islam^2,7*^

*^1^Department of Civil Engineering, Chittagong University of Engineering & Technology, Chatttogram, 4349, Bangladesh*

*^2^School of Environmental and Life Sciences, The University of Newcastle (UoN), Callaghan, NSW 2308, Australia*

*^3^Department of Geography and Environment, Islamic University, Kushtia 7003, Bangladesh*

*^4^Materials and Chemical Processing Laboratory, The University of Adelaide, Adelaide SA 5000, Australia*

*^5^Department of Public Administration, Shahjalal University of Science & Technology, Sylhet 3114, Bangladesh*

*^6^Department of Agricultural Economics, Faculty of Agribusiness Management, Sher-e-Bangla Agricultural University, Sher-e-Bangla Nagar, Dhaka 1207, Bangladesh*

*^7^Department of Applied Chemistry and Chemical Engineering, Islamic University, Kushtia 7003, Bangladesh*

*^*^****Corresponding Author:*** Dr. Rafiquel Islam; Email: [rafiquel.islam@newcastle.edu.au](mailto:rafiquel.islam@newcastle.edu.au)


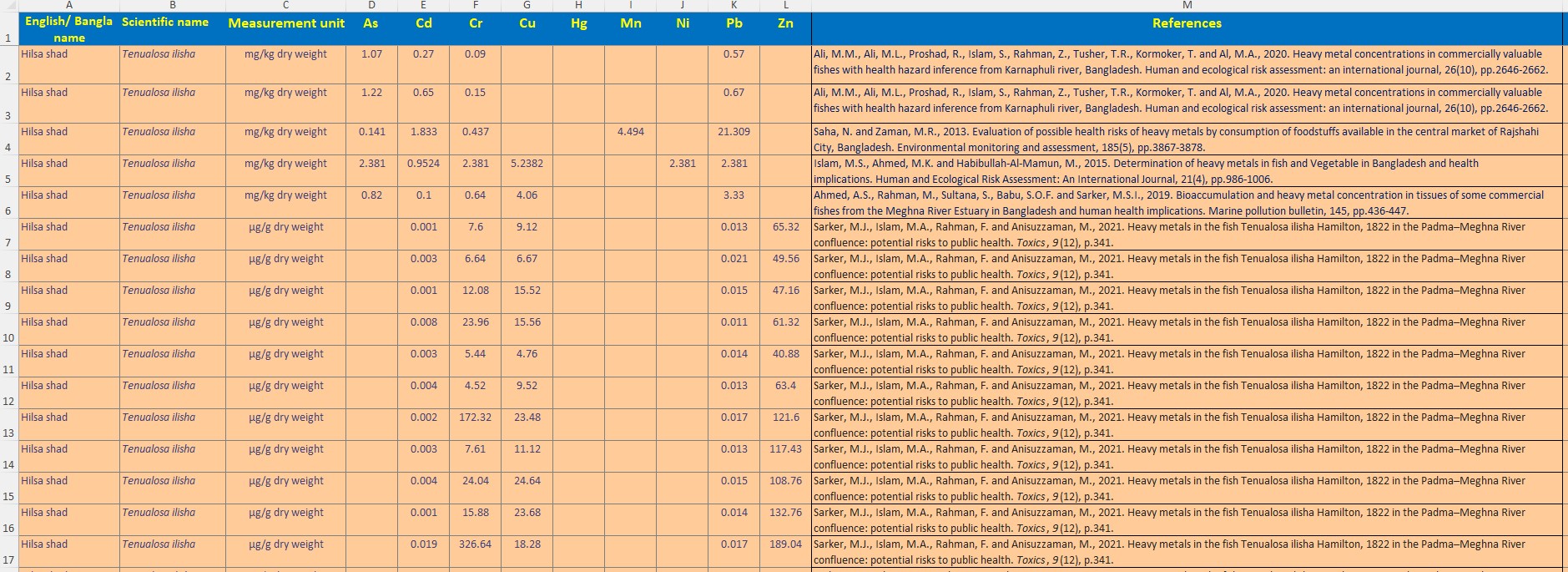


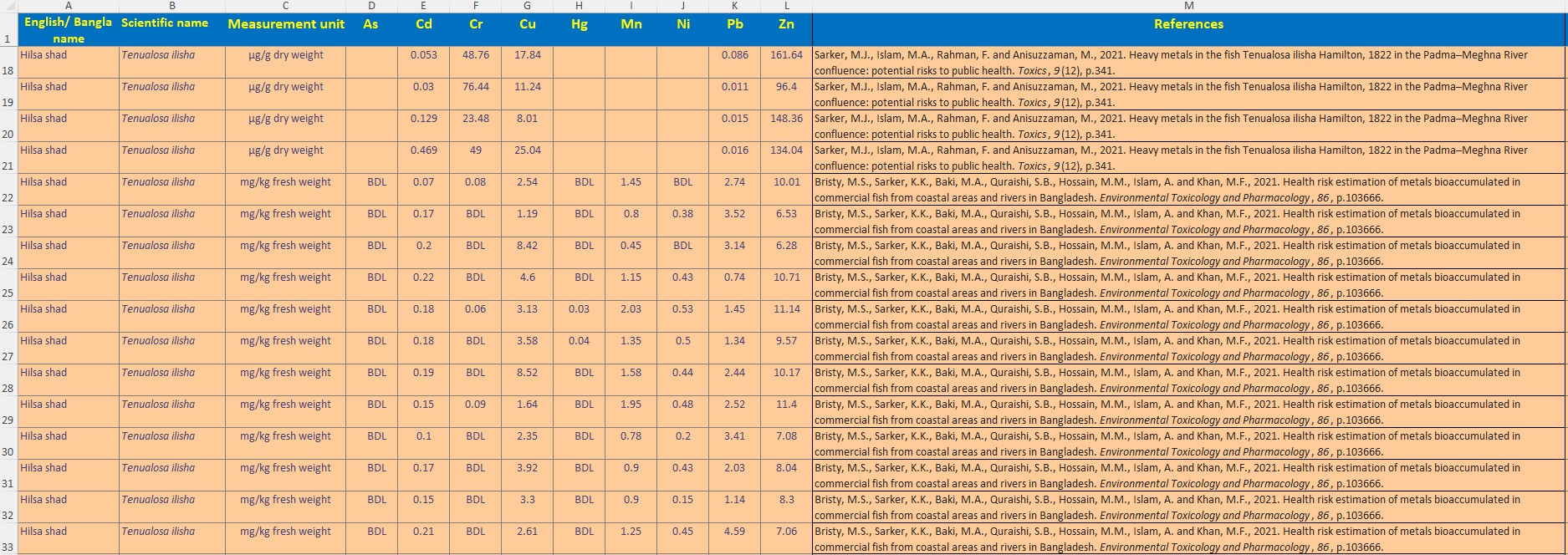


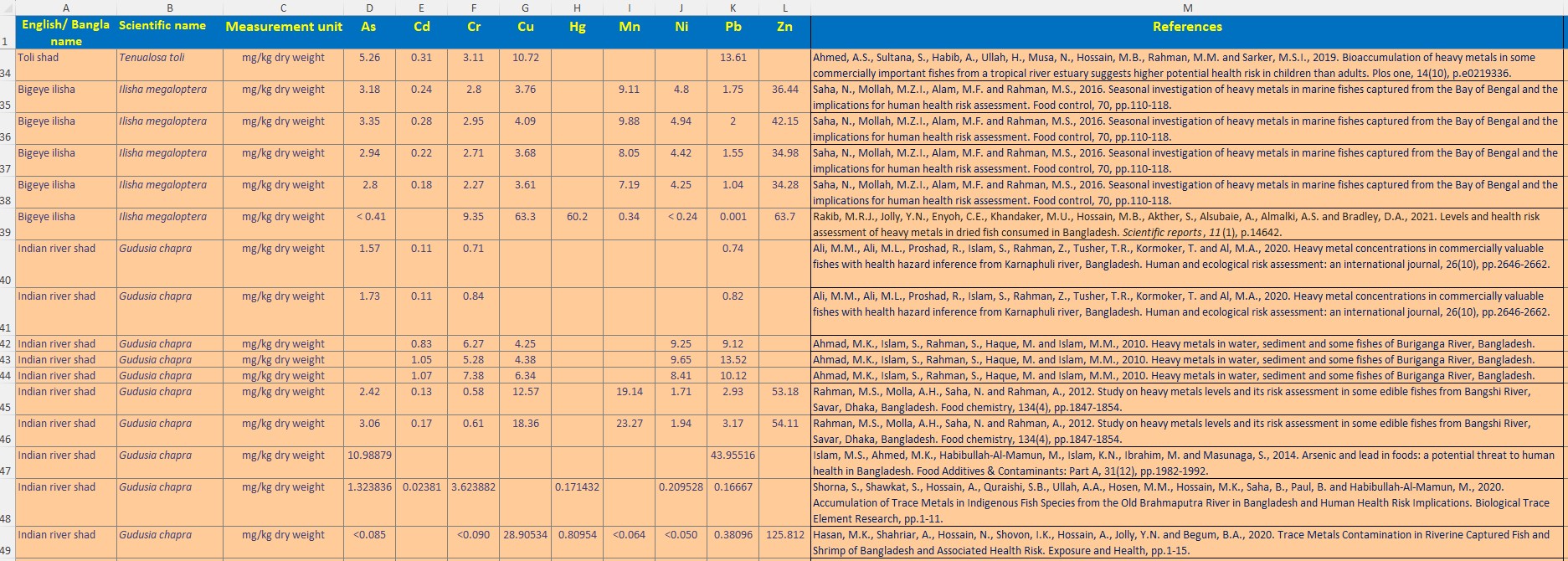


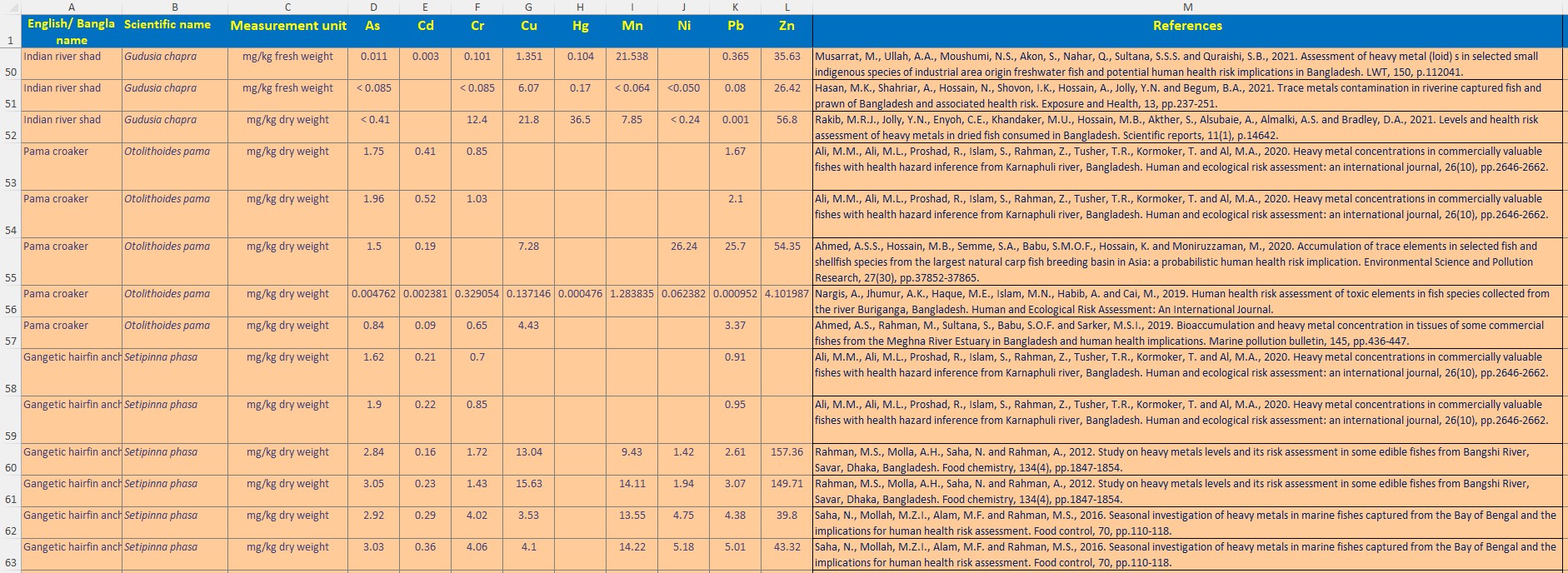


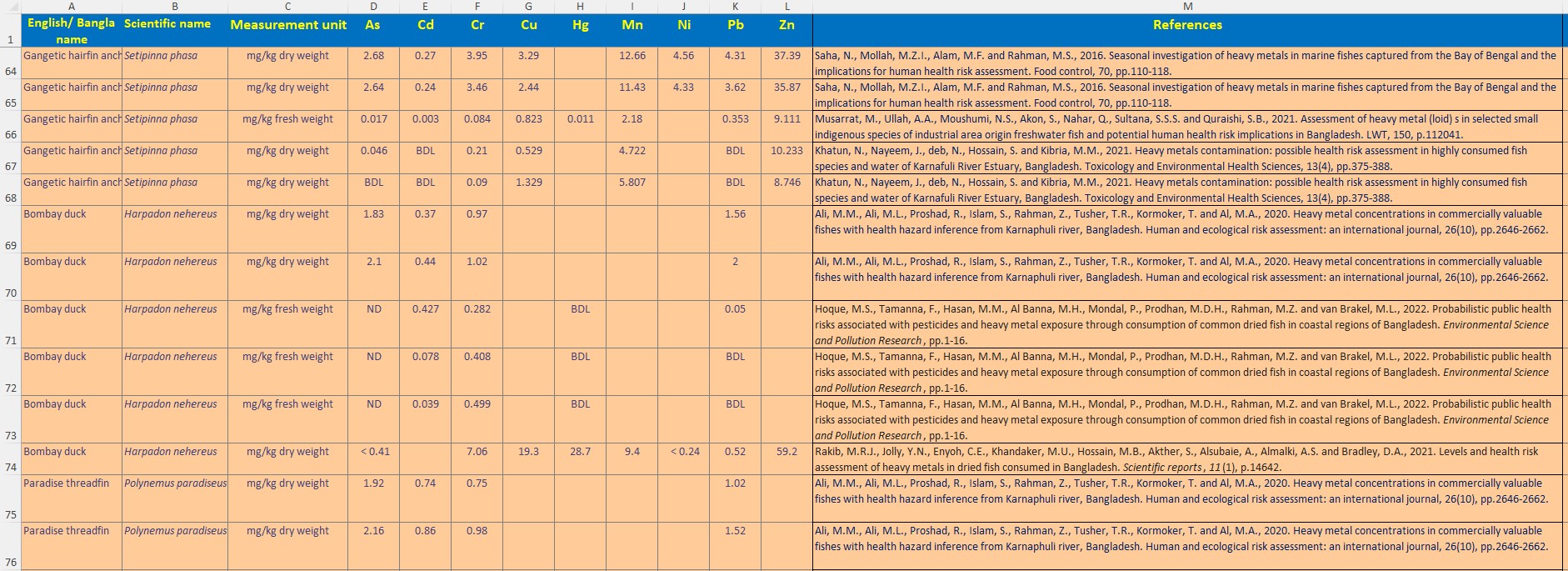


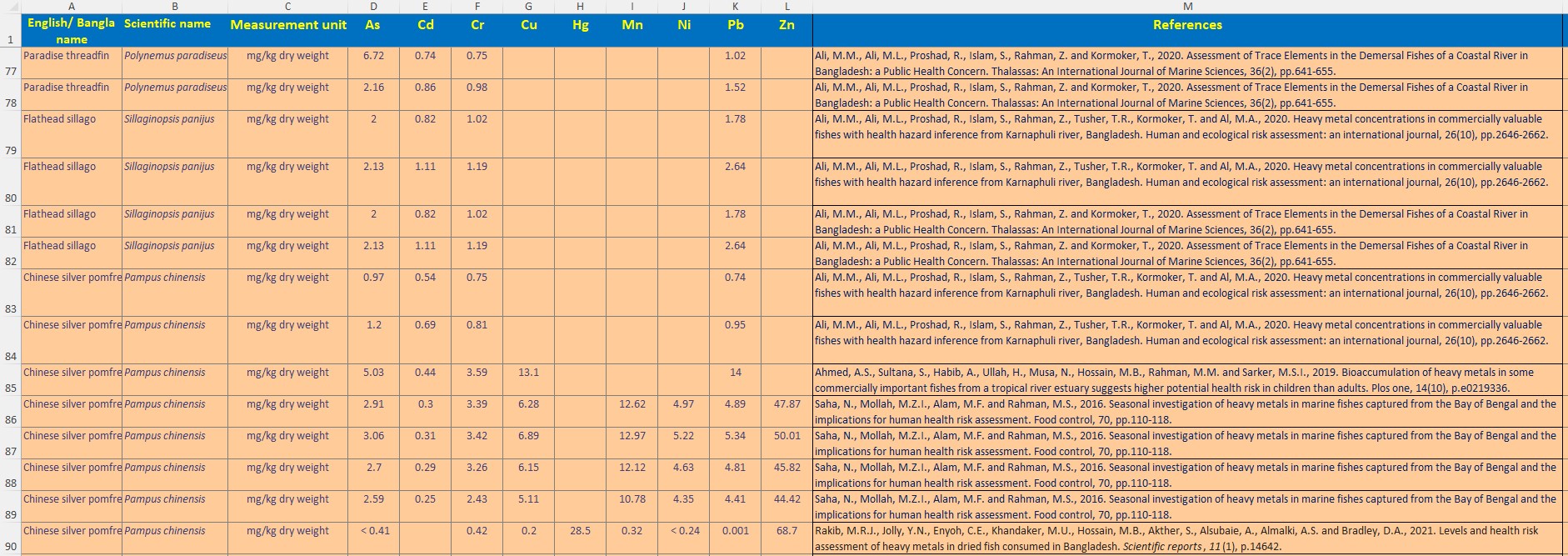


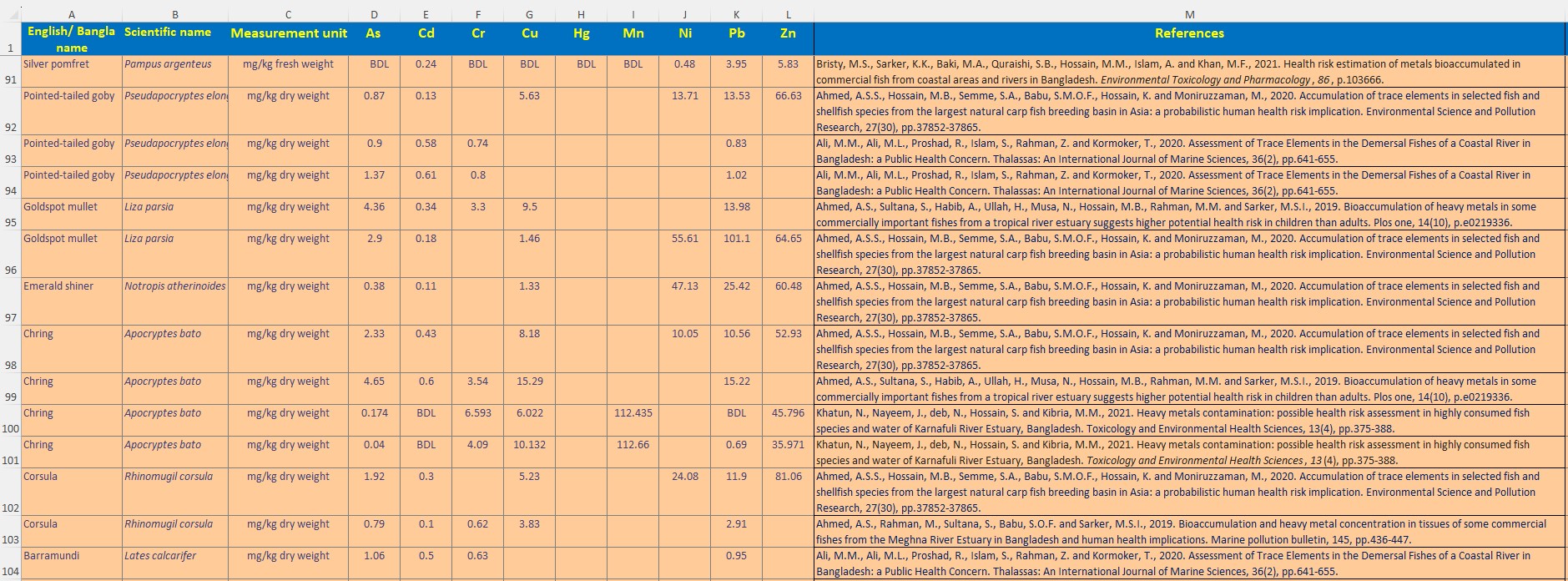


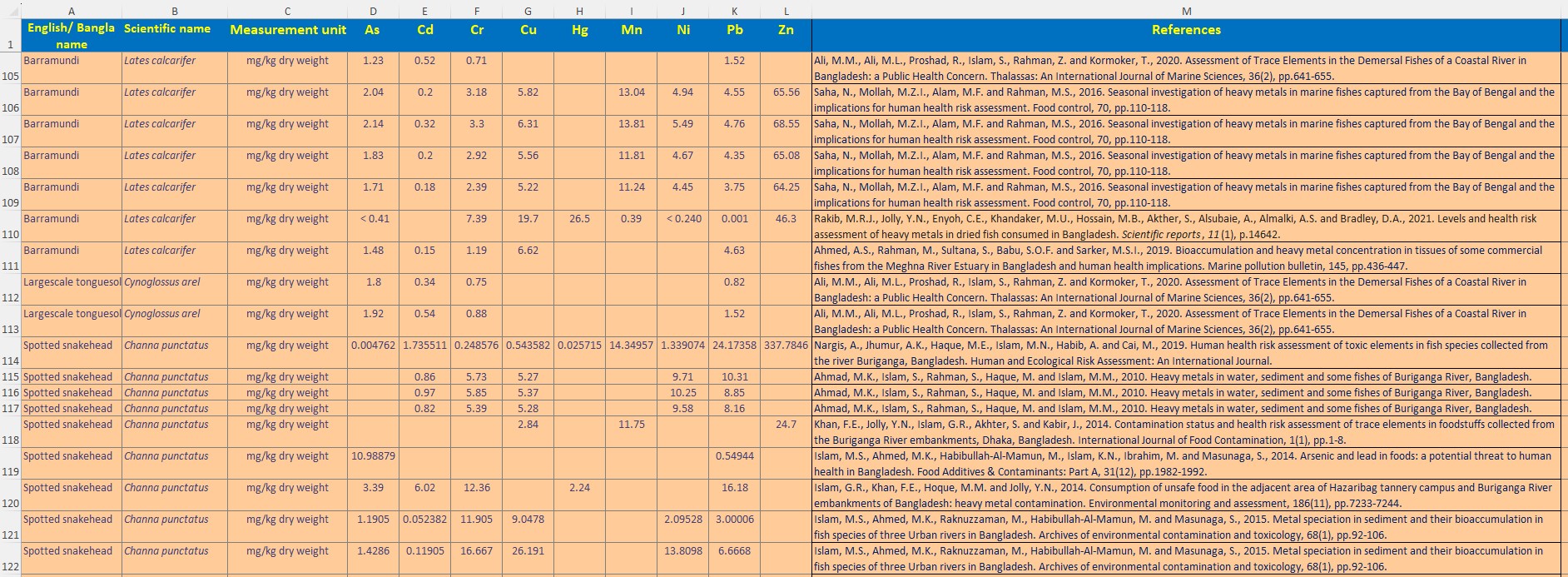


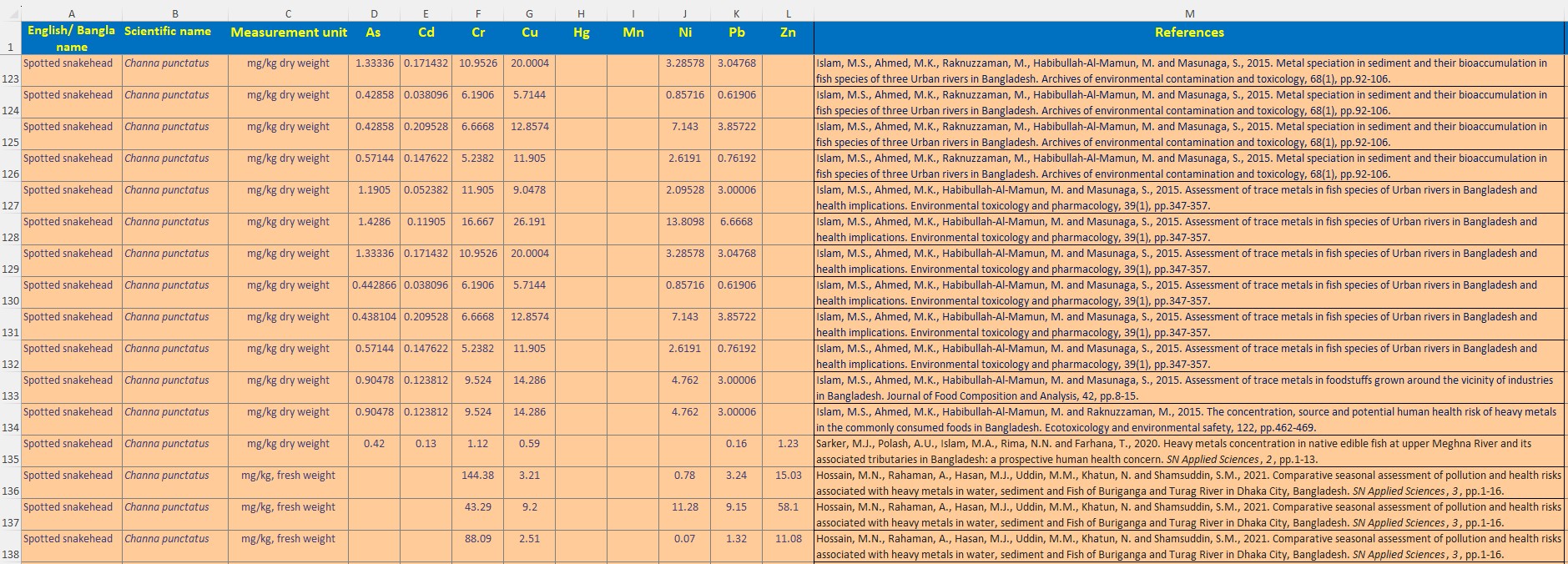


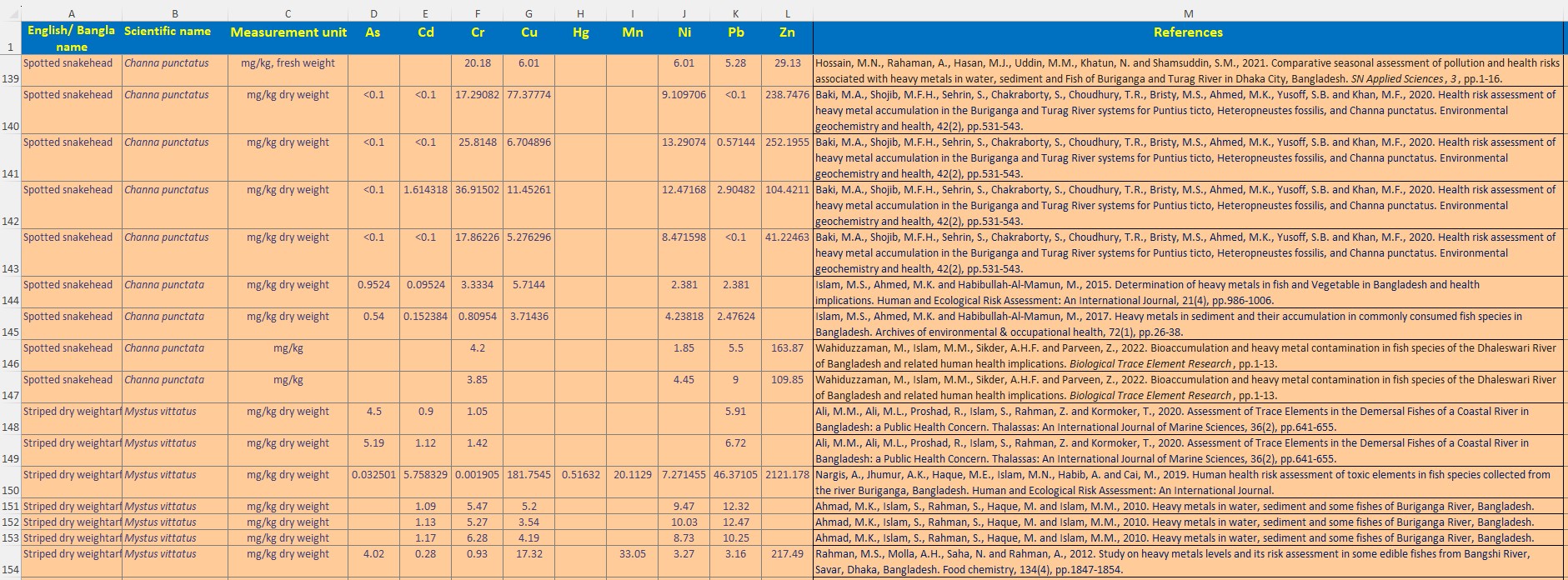


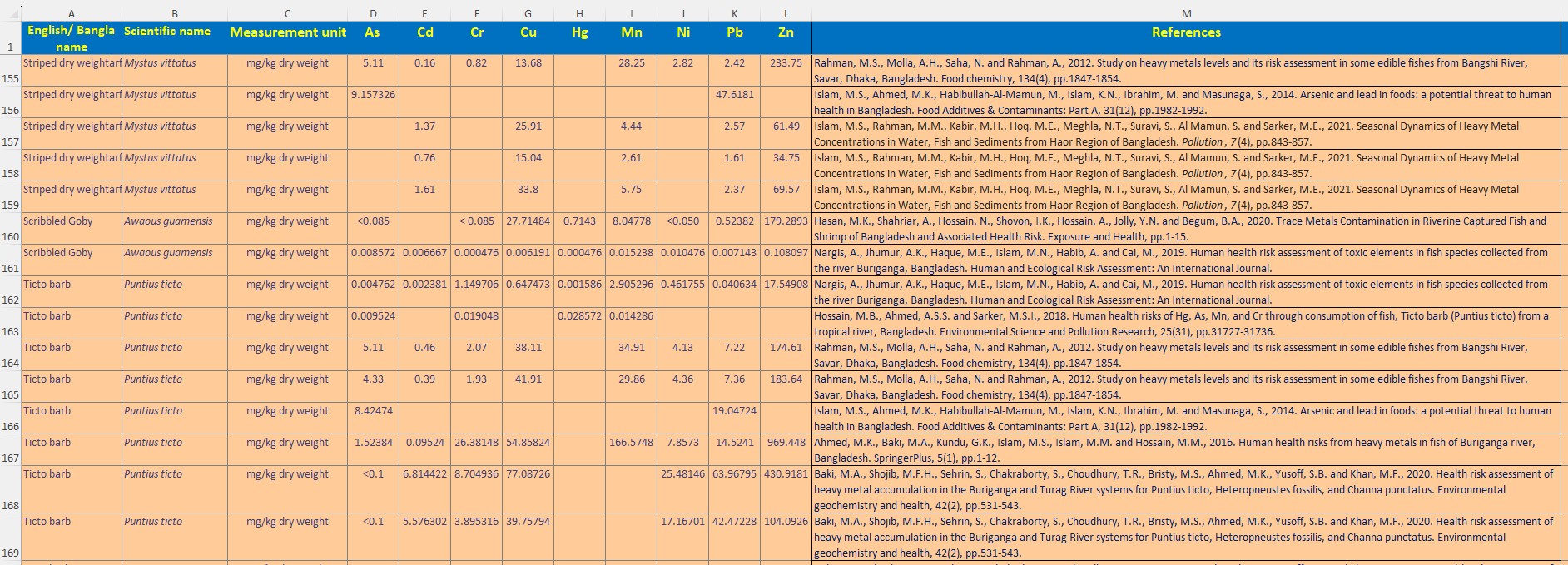


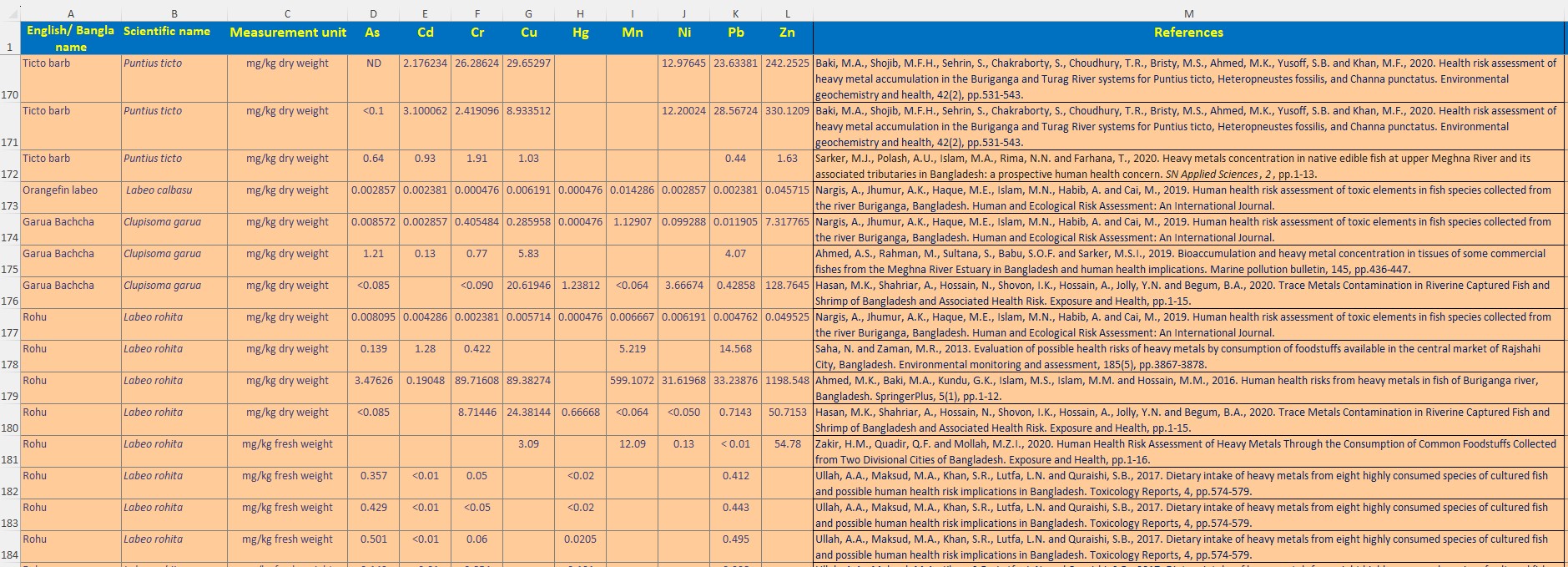


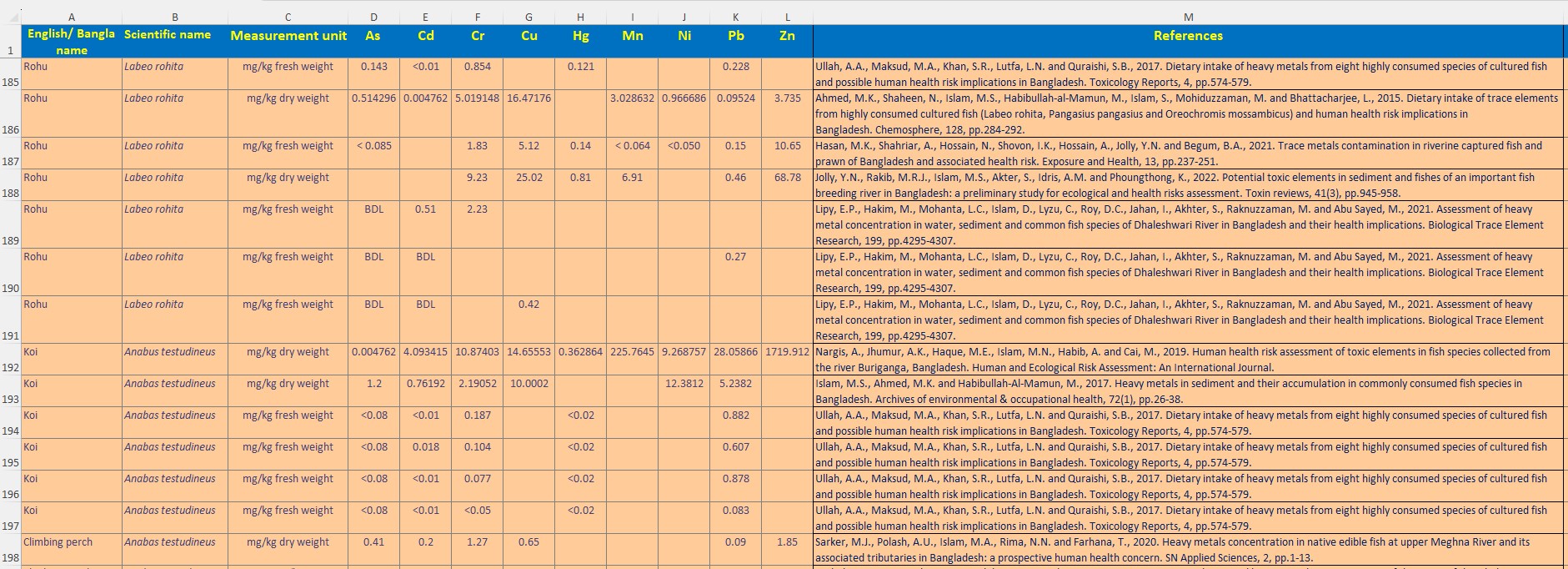


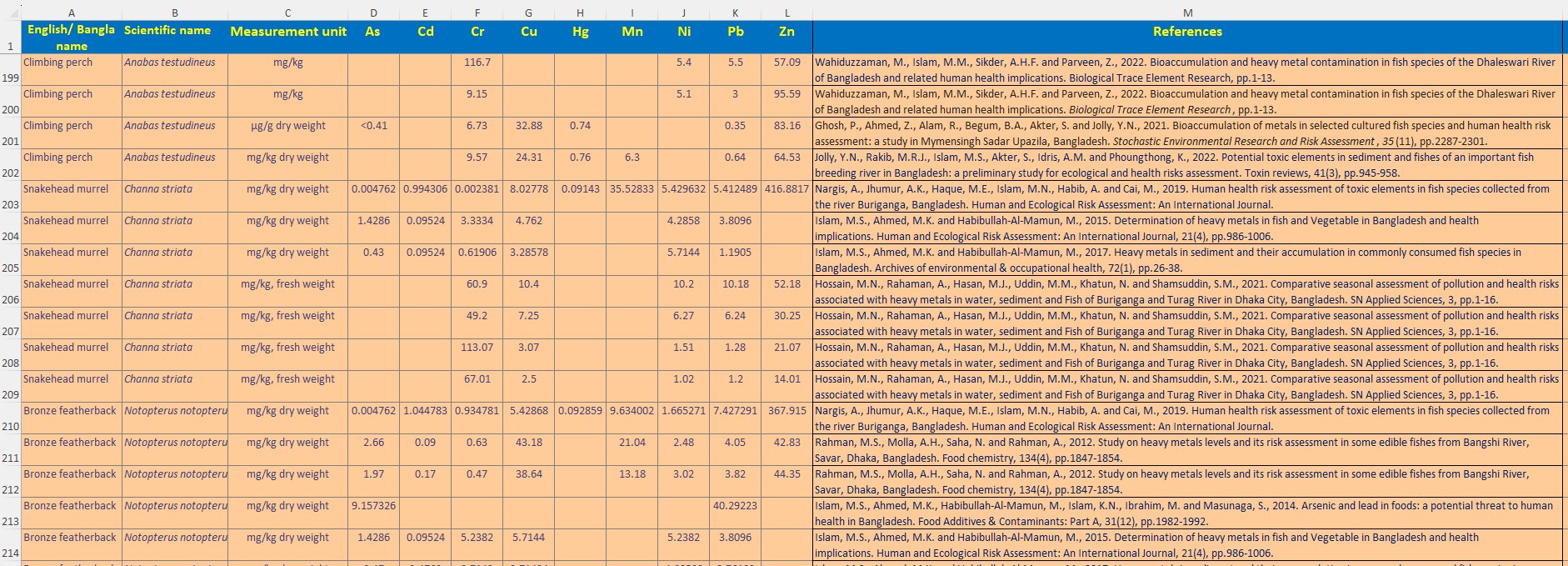


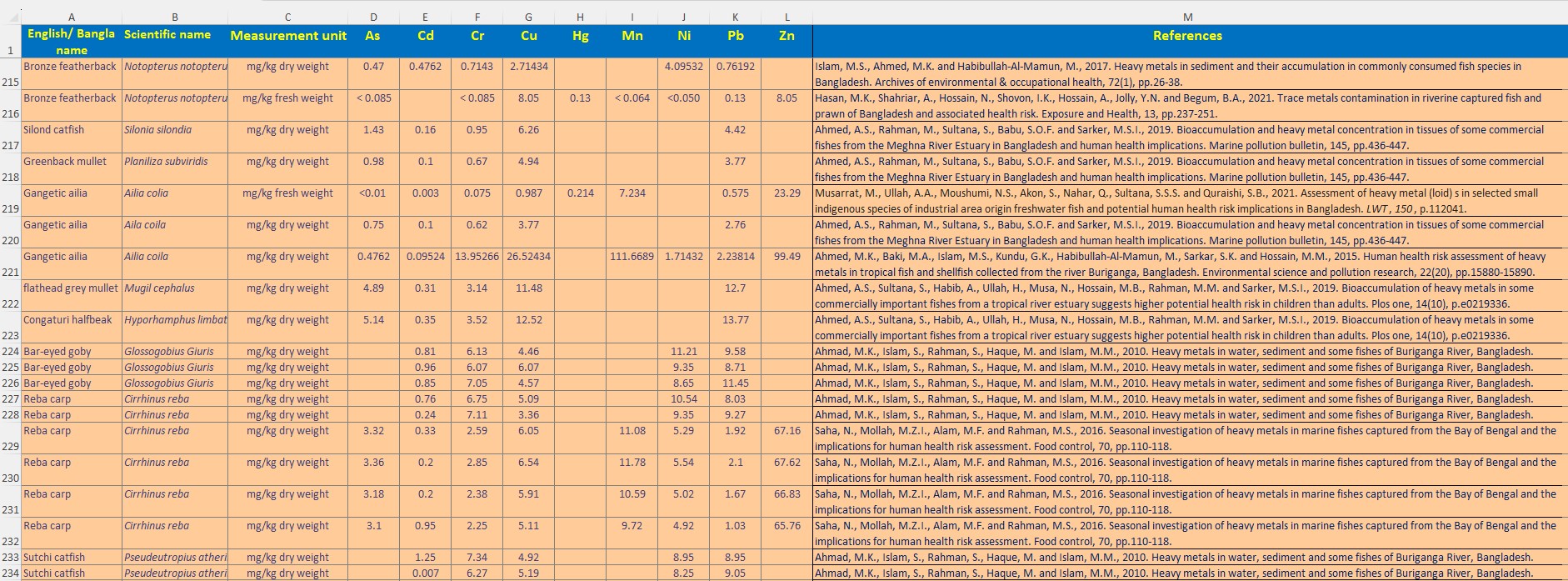


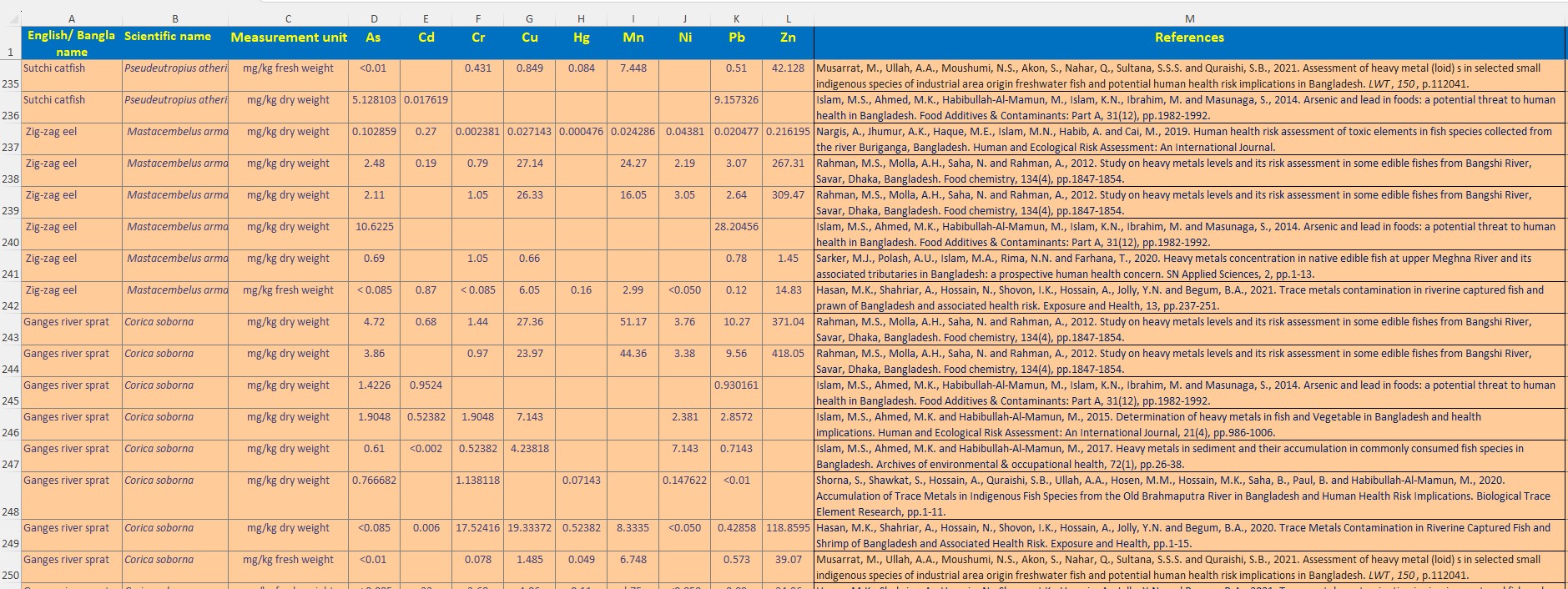


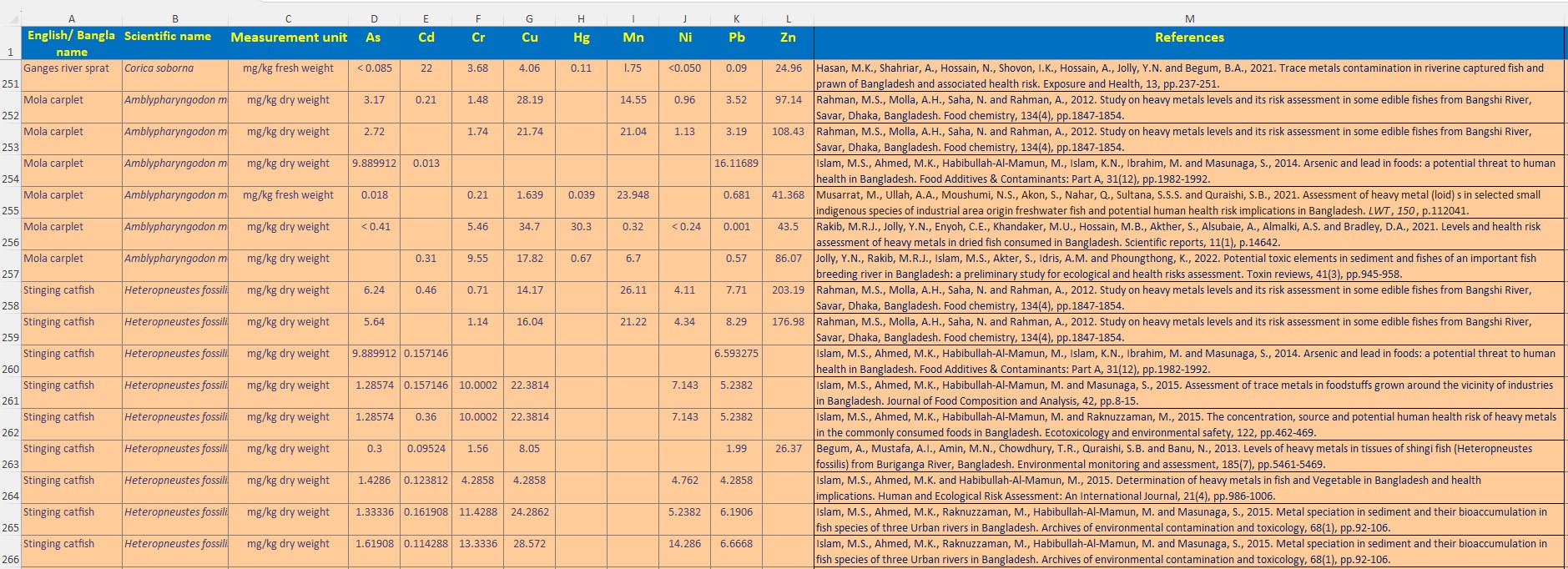


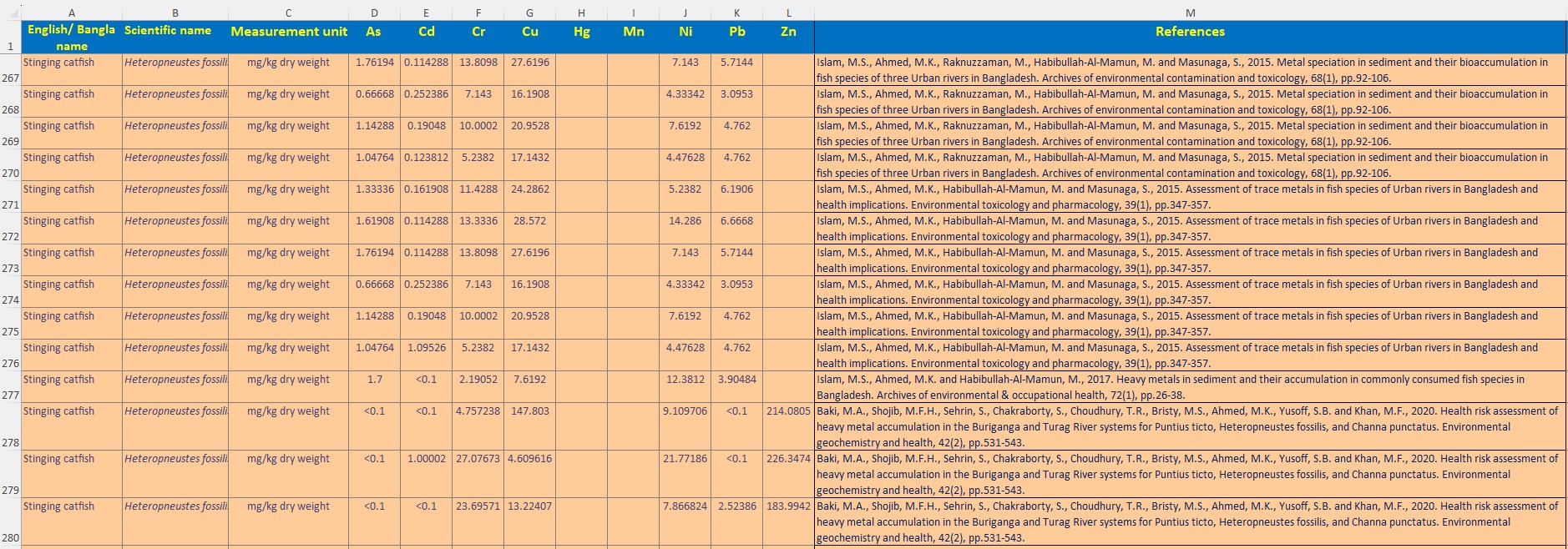


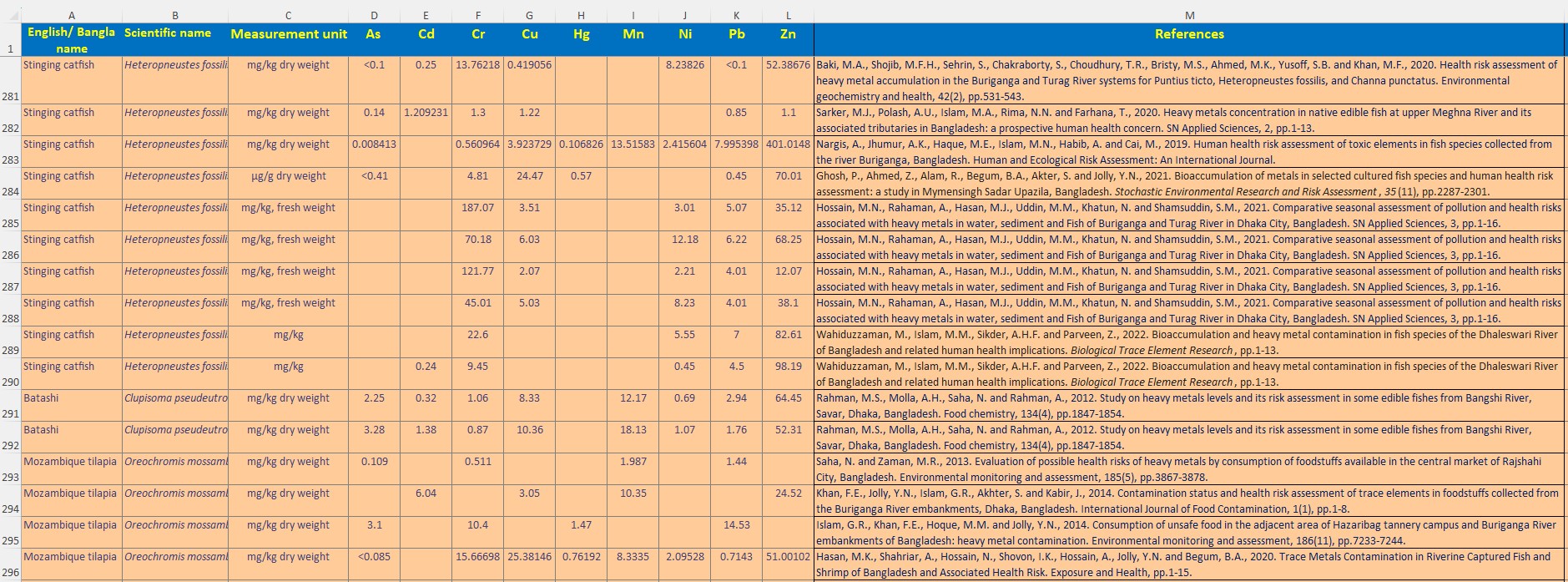


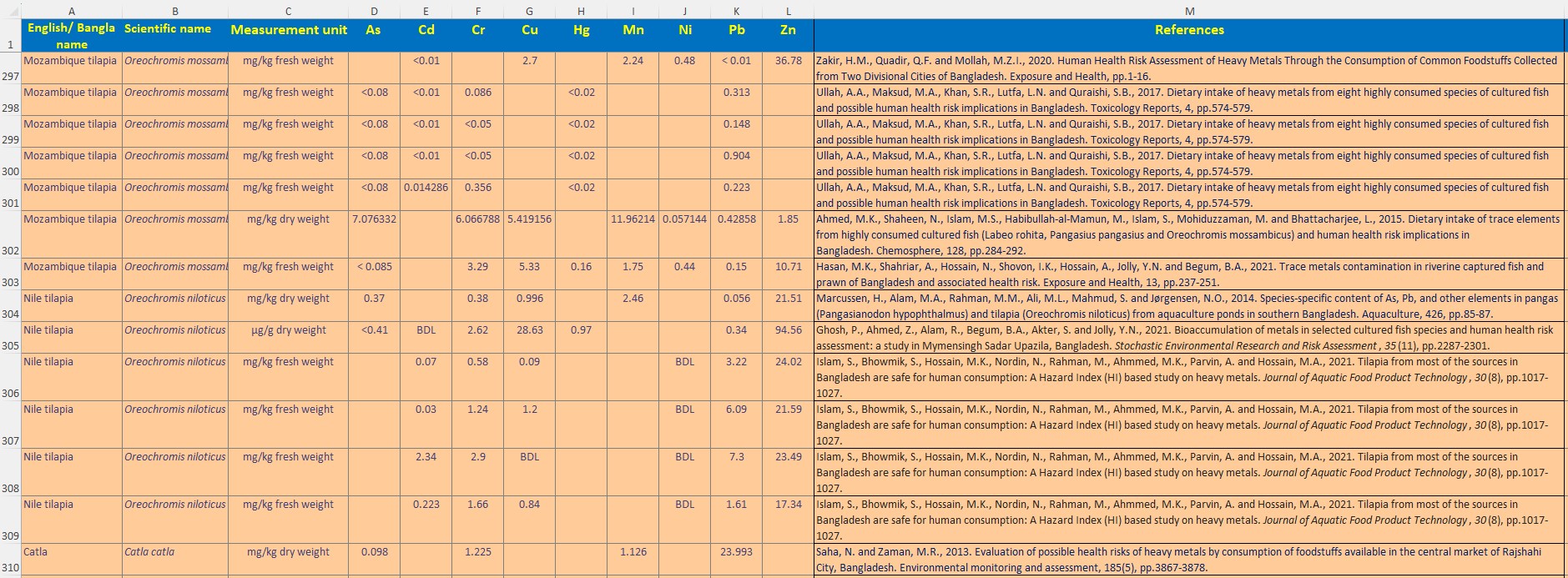


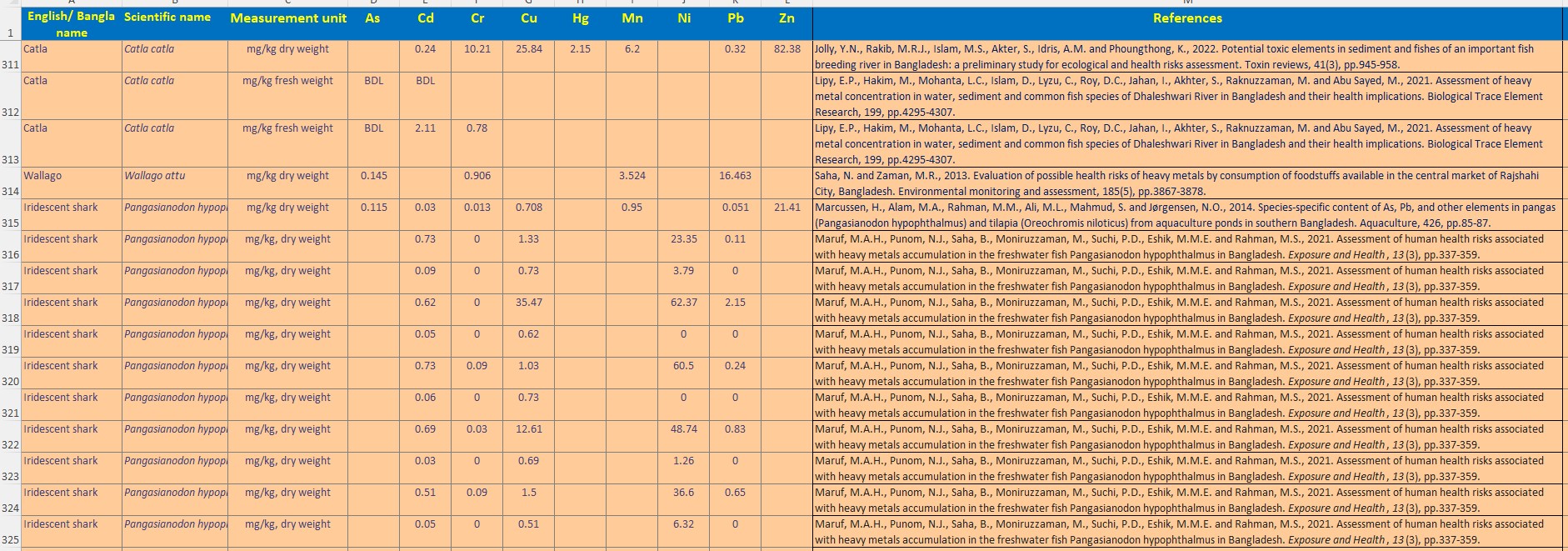


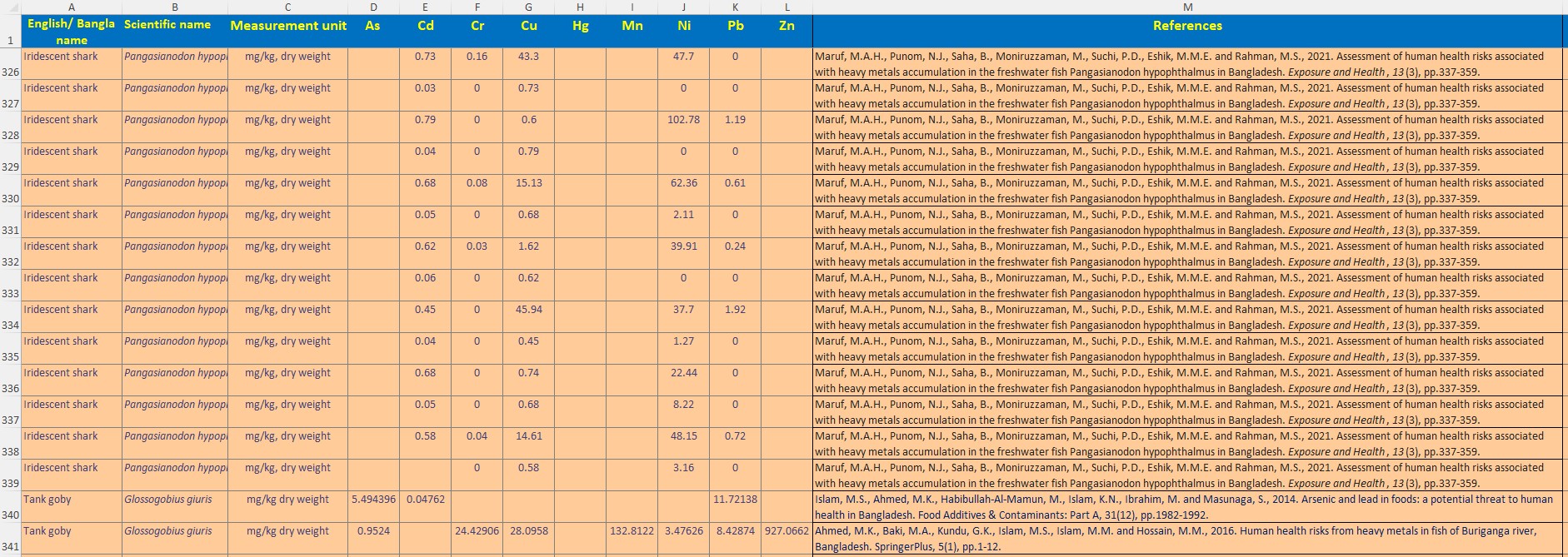


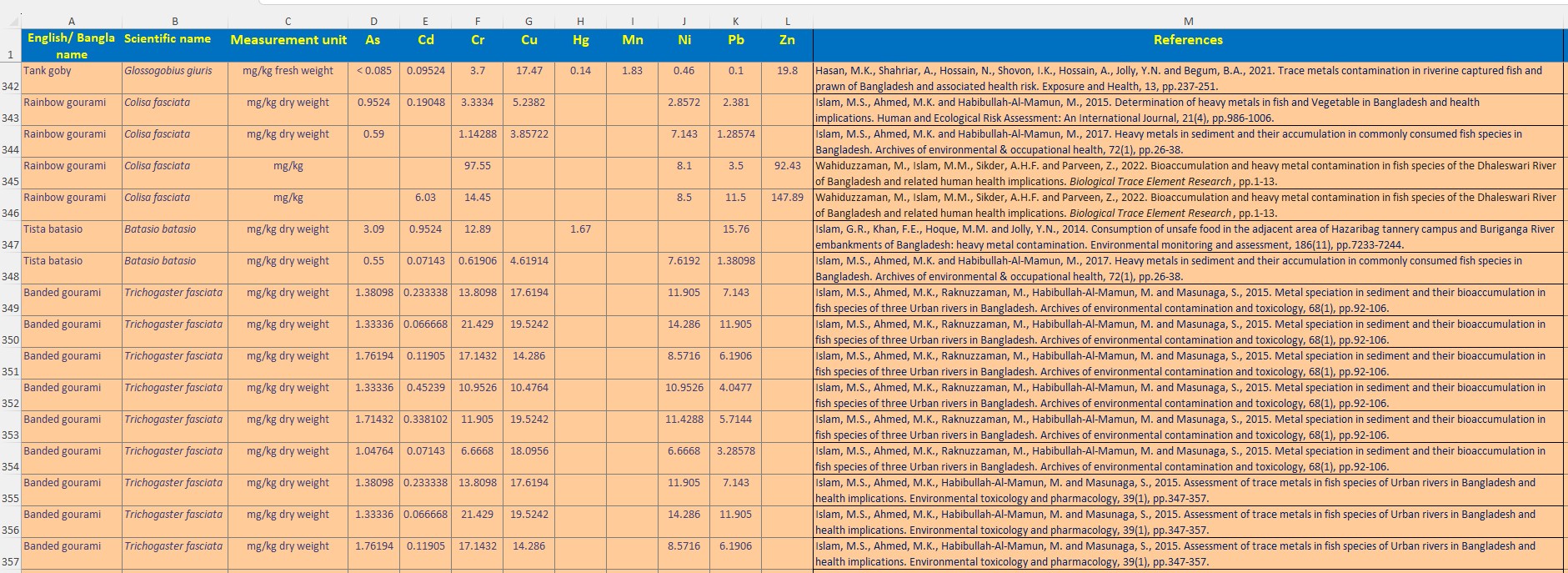


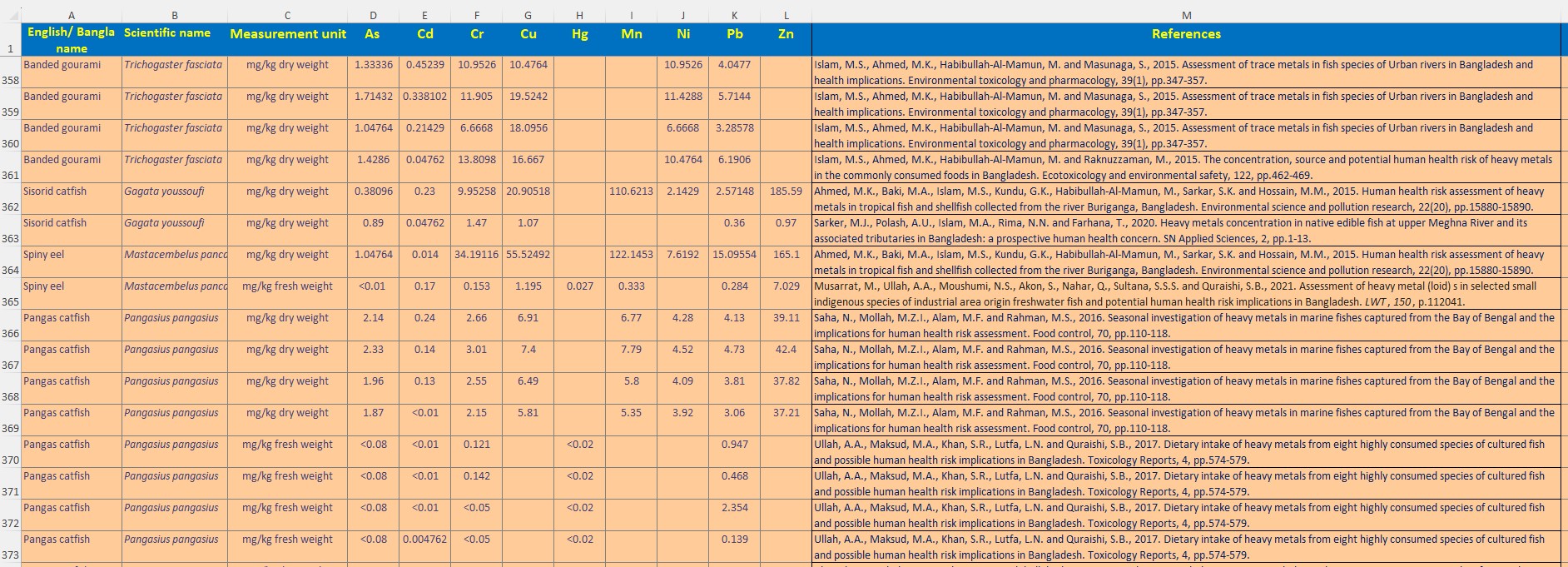


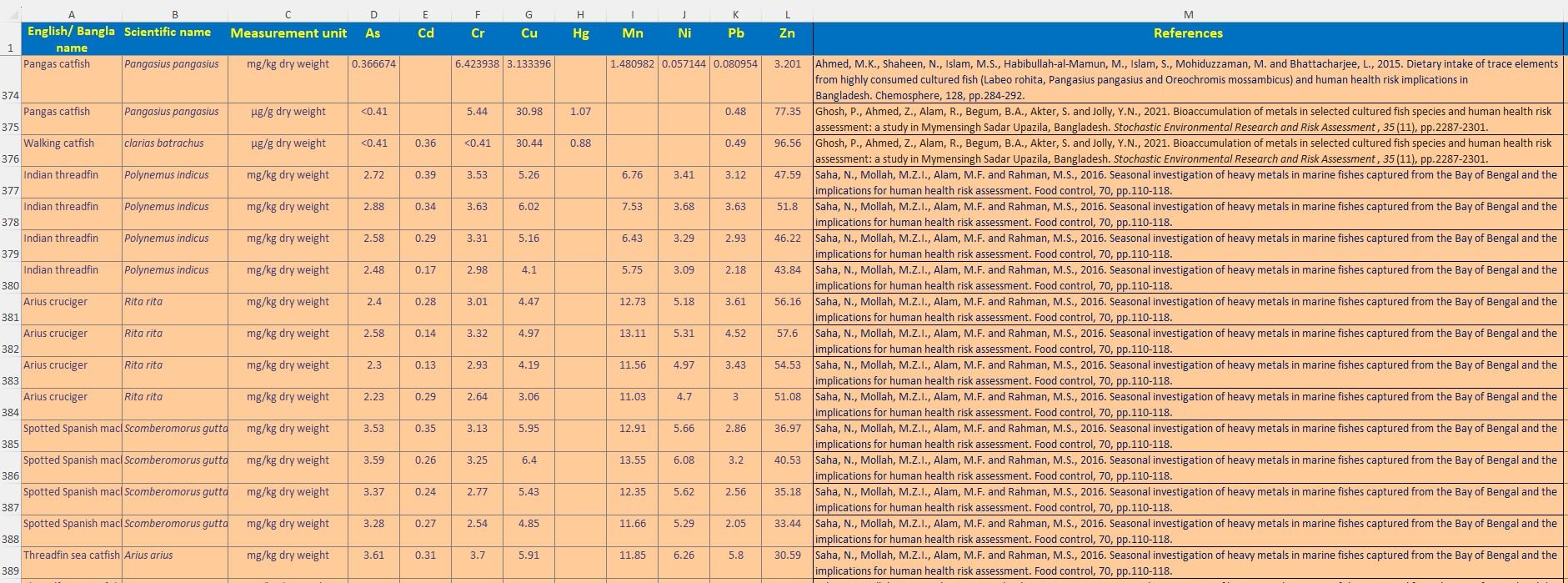


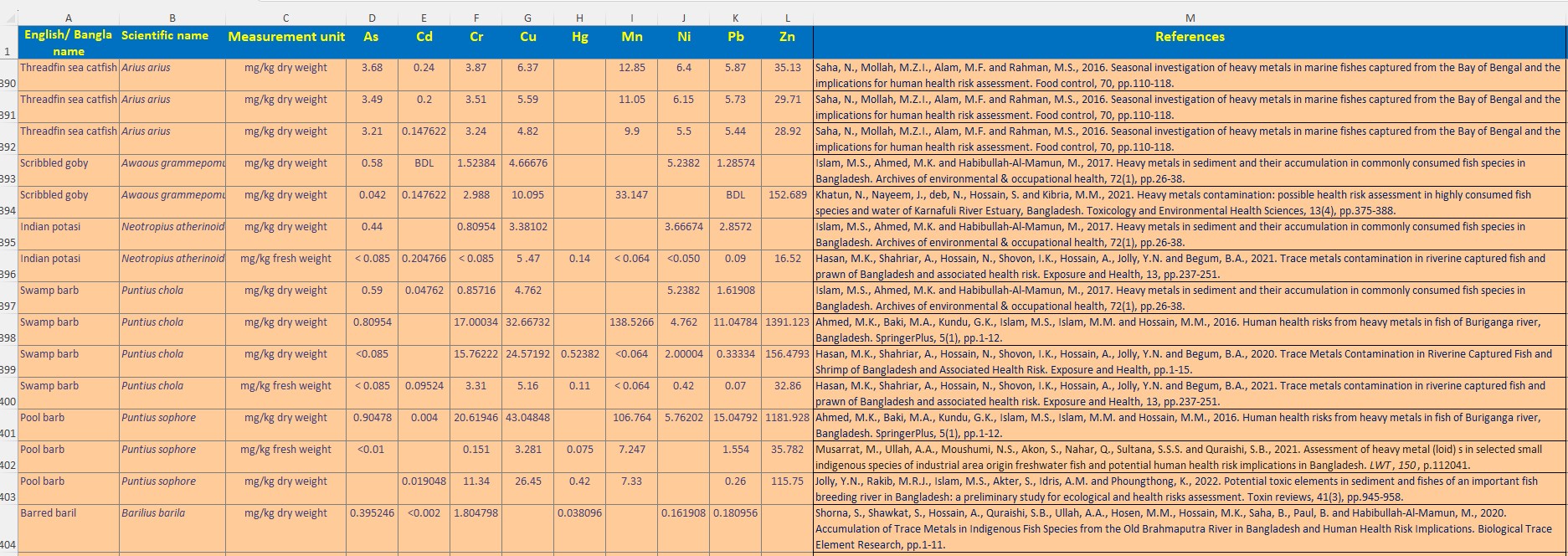


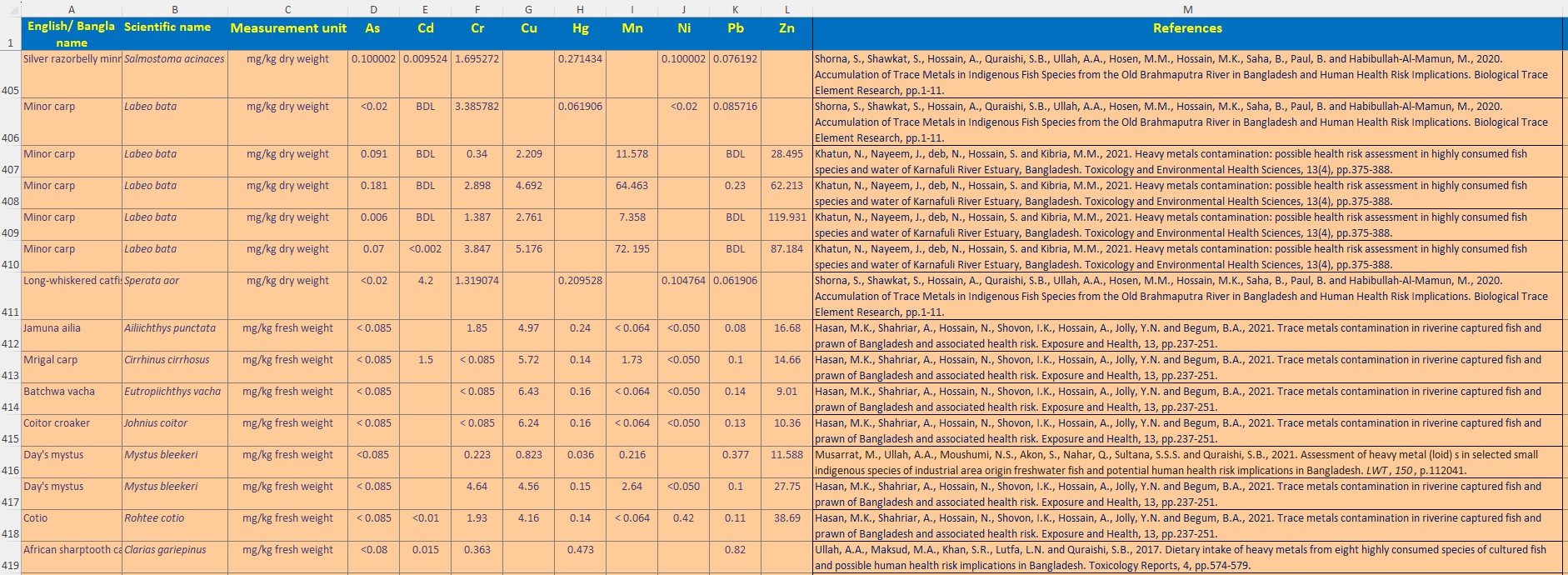


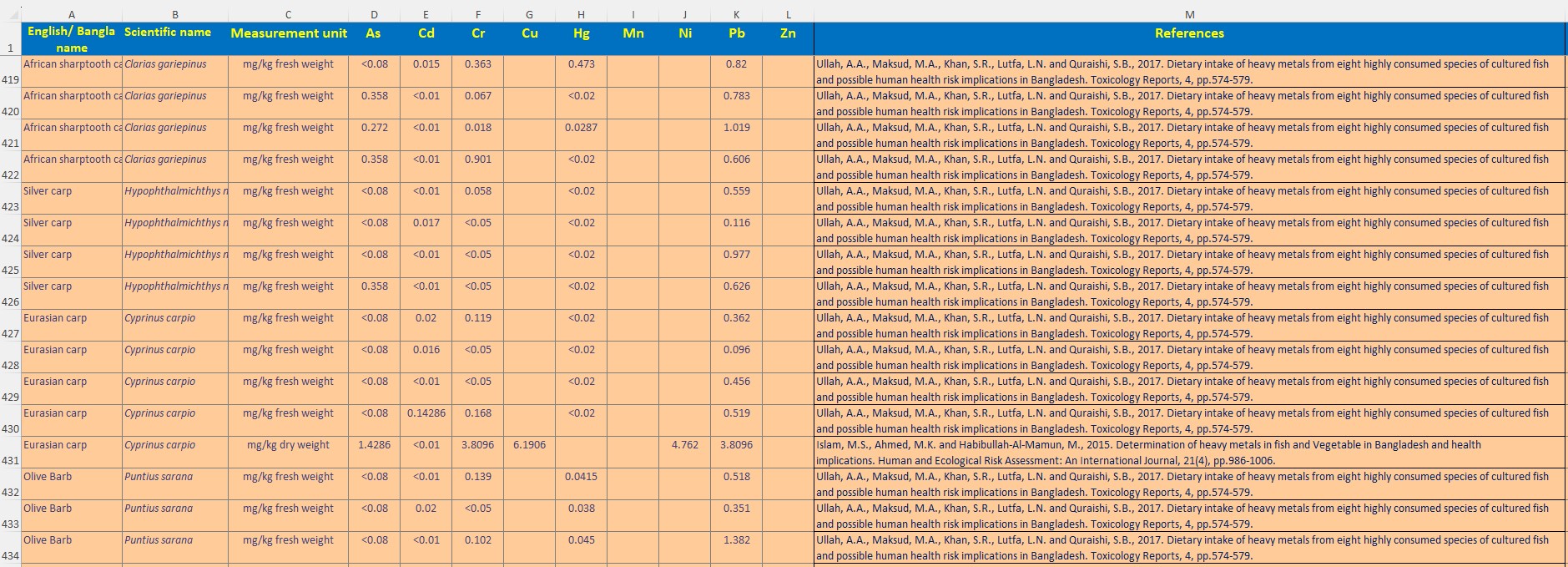


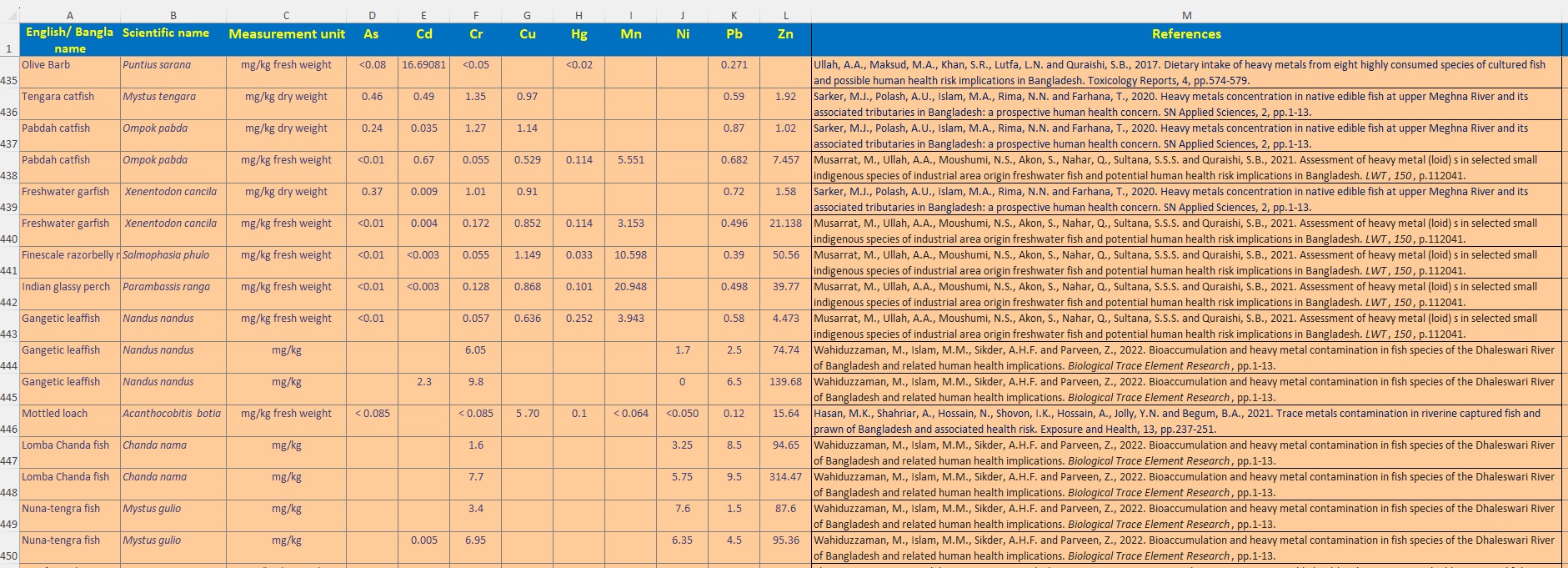


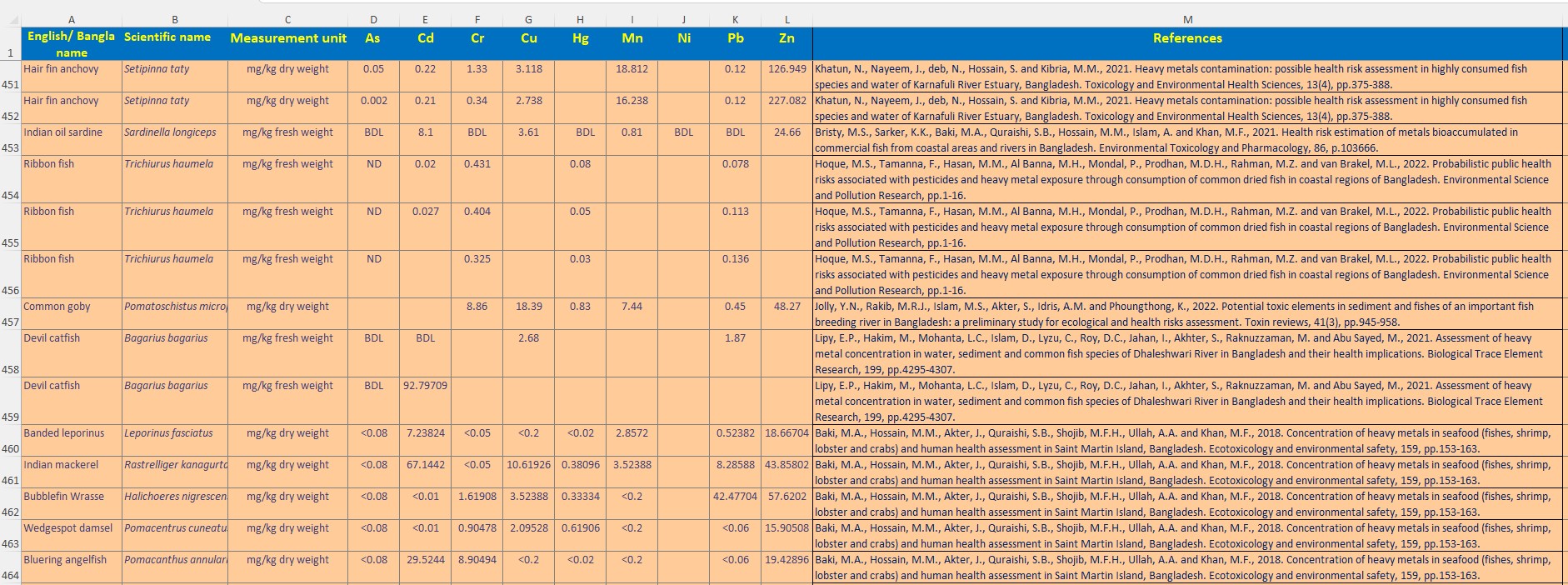


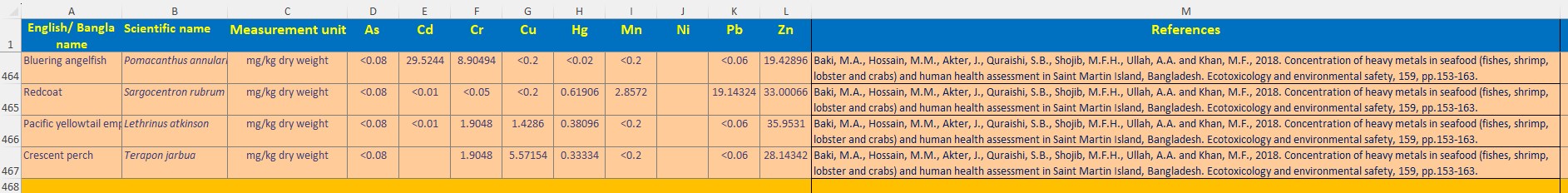

Supplement: Supplementary file 2 — Supplementary file2 (DOCX 15141 KB) [file 12011_2024_4363_MOESM2_ESM.docx]
